# Countrywide Spatial Variation of Potentially Toxic Element Contamination in Soils of Turkey and Assessment of Population Health Risks for Nondietary Ingestion

**DOI:** 10.1021/acsomega.2c04261

**Published:** 2022-10-04

**Authors:** Aysegul Yagmur Goren, Mesut Genisoglu, Yiğithan Kazancı, Sait C. Sofuoglu

**Affiliations:** Department of Environmental Engineering, Izmir Institute of Technology, Gulbahce, Urla 35430, Izmir, Turkey

## Abstract

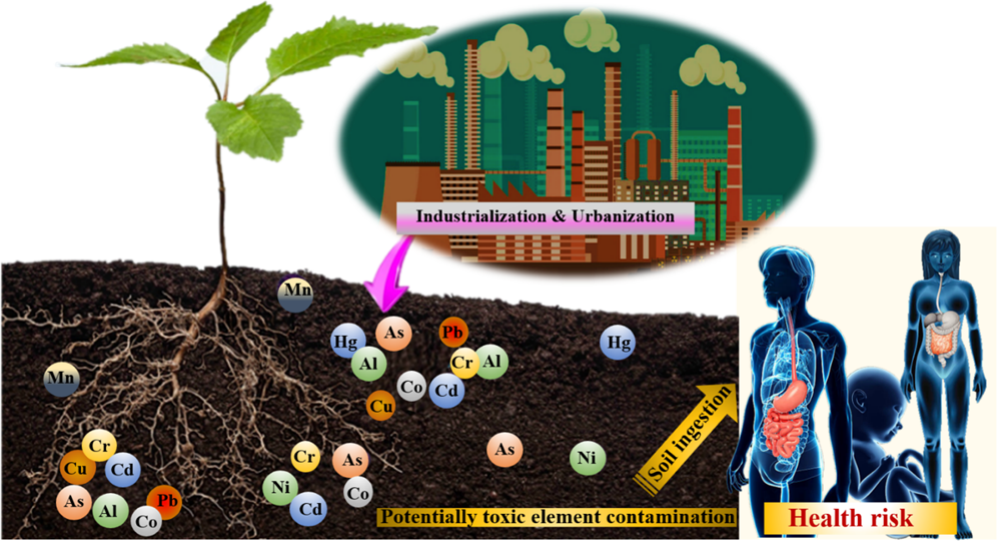

Countrywide surface soil concentrations of potentially toxic elements (PTEs) in Turkey were reviewed in the Web of Science database. A total of 93 papers were investigated to compose a PTE dataset for determining spatial variations and estimating exposure and health risks. Al, As, Cd, Co, Cr, Cu, Fe, Mn, Ni, Pb, and Zn were selected as PTEs in surface soil. A compiled PTE concentration dataset was used to estimate chronic toxic risks (CTRs) and carcinogenic risks (CRs) according to the deterministic and probabilistic approaches. While the CTR and CR levels of age and sex groups were estimated using a deterministic approach, population risks were estimated using a probabilistic approach. CTR and CR levels in lower age groups and female sex groups were estimated to be higher than those in higher age groups and associated male sex groups. The average CTR levels of the nondietary ingestion of As-containing soil in <11 year age groups were near/just above the threshold level, while As-associated average CR levels of adults and other age groups were estimated to be in the acceptable risk range (10^–6^ < CR < 10^–5^) and low priority risk range (10^–5^ < CR < 10^–4^), respectively. As-, Cr(VI)-, and Pb-associated upper-bound CR levels of the Turkish population were simulated to be 5.14 × 10^–4^, 6.23 × 10^–5^, and 2.34 × 10^–6^, respectively. Health risk models show the significance of As in both chronic toxic and carcinogenic effects.

## 1 Introduction

Potentially toxic element (PTE) contamination in soil has always been an important issue with continuing industrialization and urbanization. PTEs may originate from both anthropogenic and natural sources. Natural contamination arises from the weathering of parent rocks, volcanic eruptions, soil erosion, forest fires, and wind dusts, while anthropogenic sources include industrial manufacturing, agricultural practices, mining, traffic emissions, and fuel combustion.^1^ Currently, the impact of anthropogenic sources is estimated to exceed that of natural sources due to ever-increasing industrial activities and urbanization to meet the needs of the growing population.^2^ As a result, PTE contamination is of critical importance in industrial, urban, and suburban areas for human exposure.^3^ However, exposure in rural areas should not be disregarded because of wide mining activities, agricultural activities, and atmospheric transport from urban and industrial areas.^4−6^ Namely, rural areas are exposed increasingly to several PTEs, having contaminant sources such as biomass combustion, emissions from fertilized agricultural soils, and resuspended road dust.^7^ For instance, in agricultural fields, the application of mineral and animal waste fertilizers may cause PTE accumulation in soils. Particularly, commercial fertilizers contain PTEs such as Cu, Zn, and Pb, and uncontrolled fertilization can cause PTEs to accumulate in the soil.^8^ Moreover, the PTE-containing fine particles may be transported over long ranges, resulting in considerable increases in PTE concentrations in soil, sediment, and dust at great distances from the contaminant source.^9,10^

Commonly present PTEs in soils include aluminum (Al),^11^ arsenic (As),^12^ cadmium (Cd),^13^ chromium (Cr),^14^ cobalt (Co),^15^ copper (Cu),^11^ lead (Pb),^11^ manganese (Mn),^11^ mercury (Hg),^16^ nickel (Ni),^11^ and zinc (Zn).^11^ Among these PTEs, As, Cd, Hg, and Pb are also reported in the top 20 Hazardous Substances of the Agency for Toxic Substances and Disease Registry (ATSDR) and the United States Environmental Protection Agency (USEPA).^17,18^ PTE contamination could have a severe impact on human health and the soil ecosystem due to their toxicity, non-biodegradability, and bioaccumulation in the food chain when they are in bioavailable forms.^19,20^ Exposure to PTEs is possible through all three routes: dermal contact, direct/indirect ingestion, and inhalation of suspended dust.^21^ Overall, these PTEs may cause health effects such as cancer; chronic anemia; cardiovascular diseases; and damage to the brain, bones, skin, kidneys, and nervous system.^22^

There are several studies on exposure and risk assessment through accidental (nondietary) ingestion of soil conducted around the world.^23−25^ For instance, Izquierdo et al.^26^ studied metal contamination in urban gardens and associated human health risks. They reported that the children playing in the garden and humans who eat the vegetables produced in gardens have the highest risk associated with accidental ingestion of soil. The potential risks of heavy metals on human health through ingestion, dermal contact, and inhalation of soil were reported to be significant in urban and industrial areas of the Niger Delta.^27^ Huang et al.^28^ studied health risks associated with ingestion, inhalation, and dermal contact of soil heavy metals for different land uses, namely, residential land, forest land, and farm land, and reported that all examined areas were significantly affected by anthropogenic sources. Health risks associated with Pb, Zn, and As in soccer field soil induced by solid particle ingestion were assessed using measured metal(loid) gastric bioaccessibility values and found to be 40.6, 28.5, and 7.6%, respectively.^29^ Ljung et al.^30^ studied metal and arsenic distribution in soil particle sizes relevant to soil ingestion by children in urban areas and found that metal intake from deliberate soil ingestion was up to twice as high as involuntary soil ingestion of small particles. Berasaluce et al.^31^ determined a significant correlation between trace element (As, Cd, Cu, and Pb) concentration in hair and toenail and nondietary ingestion exposure. Hence, the literature shows that considerable exposures may occur in all types of settings, i.e., rural, suburban, and urban.

There are many studies on the assessment of the level of contamination in Turkish soils that span soils near mining sites of industrial and urban areas showing extensive variation in concentrations, exceeding the applicable Turkish standard at many locations by up to 25-fold, for which bibliographic information are provided in Supporting Information 2, Sheet 1. However, there is no review publication in the literature on soil PTE levels in Turkey, and associated health risks for accidental ingestion exposure have not been assessed. The main objectives of this study were (i) to review the PTEs, extraction and analysis methods, and concentrations of surface soil in Turkey, (ii) to investigate spatial variation and influential variables, and (iii) to estimate PTE exposure by accidental soil ingestion and associated health risks with deterministic and probabilistic approaches. This study has mediated an opportunity to show the effect of assuming point estimates recommended for other nations in the literature instead of using parameter values specific to the subject population.

## 2 Materials and Method

### 2.1 Literature Survey and Data Collection

Countrywide PTE soil concentrations in Turkey were reviewed in this study using the Web of Science (WOS) database. WOS is the oldest, most widely used, and authoritative database of research publications and citations.^32^ The period of 2008−2018 was considered in the current study, which focused only on PTE-contaminated soils in Turkey. First, the PTE, trace element, soil, and Turkey were searched in the WOS database. However, insufficient data were available using these limited and specified search criteria. Therefore, the heavy metal keyword was included in this review. The “heavy metal” and “Turkey” keywords were searched to obtain more data with consistent accuracy in the WOS database using advance search with the “((ALL = (Turkey)) AND ALL = (Heavy metal)) AND ALL = (soil)” field tag, resulting in 579 published articles which then reduced to 93 articles that report surface soil concentrations based on their abstracts. The papers were reviewed in a two-step method: first, the titles and abstracts were queried for relevance, and second, the full texts were surveyed that were considered potentially thematic. We particularly focused our search to include research papers that were original scientific papers that had abstracts and full texts. We also focused on surface soil contamination studies and did not include works that were mainly focused on subsurface soil contamination. For studies to be included, we needed to access the full texts, and the work had to report pollutant concentrations and soil sampling depth. Moreover, when a study was the subject of several articles, we utilized all related articles for a more realistic evaluation.

### 2.2 Exposure and Risk Assessment

Exposure assessment was conducted by calculating potential accidental soil ingestion dose. Accidental ingestion exposure levels were estimated deterministically and probabilistically. While a deterministic approach was used to point estimates of risks based on the created scenarios, a probabilistic approach was used to estimate frequencies of exposure and risks for the subject population. After estimating the exposures through accidental ingestion pathway, health risks were estimated using corresponding risk factors that were published in the IRIS by the USEPA and in the Risk Assessment Information System (RAIS) by Oak Ridge National Laboratory. The average daily dose (ADD) was estimated considering chronic toxic health effects using eq 1. Individual PTE concentrations were used in the deterministic approach. PTE concentrations were fitted with a probability distribution using Crystal Ball software (Oracle Inc.) for a probabilistic approach. The mean accidental soil ingestion rate value of 20 mg/day reported for adults in the Exposure Factors Handbook^33^ was considered. The exposure frequency was assumed to be 350 days/yr. The exposure duration of 75 years for adults was also taken from the Exposure Factors Handbook. Chronic toxic risk (CTR) was estimated based on the reference dose (RfD) of individual PTEs using eq 2. Individual female and male adult body weights were taken from the Exposure Factors Handbook by the USEPA for a deterministic approach.^33^ However, a combined probability distribution of female and male body weights previously constructed for Turkey was used for a probabilistic approach.^34^ Averaging time was assumed to be equal to exposure duration.
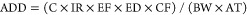
1
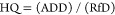
2where ADD: average daily dose (mg/(kg-day)); C: concentration (mg/kg); IR: ingestion rate (mg/day); EF: exposure frequency (day/yr); ED: exposure duration (yr); CF: conversion factor (0.000001); BW: body weight (kg); AT: averaging time (yr); RfD: reference dose (mg/(kg-day)); HQ: hazard quotient (unitless).

Lifetime ADD (LADD) levels were also estimated to determine the carcinogenic risk (CR) levels.^35^ Averaging time in ADD (eq 1) was replaced with lifetime (LT, 75 years) to obtain LADD (eq 3).^36^ Slope factor values were obtained from the IRIS database to estimate the carcinogenic risk (CR) (eq 4)^37^
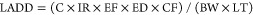
3
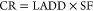
4where LADD: lifetime average daily dose (mg/(kg-day)); LT: lifetime (yr); SF: slope factor (per mg/kg-day); CR: carcinogenic risk (unitless).

Probabilistic accidental soil ingestion exposure and risk levels of PTEs were estimated using Monte Carlo simulation (*n* = 10,000 trials). PTE concentrations were fitted to a best-fitting probability distribution. The central tendency of the population soil ingestion rate was 50 mg/day, and the ingestion rate of soil generally fits the lognormal distribution.^33^ The upper percentile soil ingestion rate was reported to be 200 mg/day by Özkaynak et al.^38^ Those were used to generate probability distribution (lognormal) of the ingestion rate with an assumed location (minimum) of 0.00 mg/day. Also, the distribution of the body weight (Beta Dist: Min:0.00, Max:111.15, α:12.76, β:8.15) of the Turkish people was used in probabilistic exposure assessment to represent subject population with a specific distribution. ED, EF, and AT were assumed to be 75 years, 350 days, and 27,375 days in the probabilistic approach, respectively.

### 2.3 Statistical Analysis

Regional data were determined to be not distributed normally using Anderson Darling and Kolmogorov Smirnov tests. Therefore, exposure levels between urban, suburban, industrial, and agricultural sites were compared using the Mann–Whitney U test. Statistical tests for exposure levels are also representative for CTR and CR levels as the only independent variable is the concentration. Rural area and noncategorized location groups were not included in testing due to low sample sizes. Bootstrapping was performed to estimate variation between statistical simulations of population risks. Bootstrapping toolbox in Crystal Ball software was used to estimate uncertainties that occur due to the Monte Carlo simulation process.

## 3 Results and Discussion

### 3.1 PTEs, Extraction, and Analysis Methods

The sampling, extraction, and analysis methods; studied PTEs; and detection limits reported in the articles reviewed in this study are summarized in Supporting Information (S) 1, Table S1.1. Soil samples were mainly collected for depths of 0–20 or 0–30 cm. Several extraction methods were used to determine PTEs in soil: microwave, hot plate, DTPA (diethylene triamine pentaacetic acid), European Community Bureau of Reference (BCR) sequential extraction, and ambient temperature acid extraction methods. In these methods, the PTEs in soil phase are transferred to the liquid phase for analysis although PTEs in soil can be directly analyzed using X-ray fluorescence spectrometry (XRF), energy dispersive X-ray fluorescence spectrometry (EDXRF), and instrumental neutron activation (INN) analysis methods without involving an extraction procedure, which were not commonly employed.

The most conducted extraction methods were microwave and hot plate. In 93 articles that reported surface soil concentrations, microwave and hot plate extraction methods were used in 27% (*n* = 25) and 17% (*n* = 16), respectively. The percentages of the other extraction methods were as follows: 12% (*n* = 11) DTPA extraction, 11% (*n* = 10) ambient temperature acid extraction, 7.5% (*n* = 7) BCR sequential extraction followed by EDXRF analysis, 1.07% (*n* = 1) XRF and INN analyses without extraction.

The choices of acid mixtures were HClO_4/_HNO_3_/HCl (1:2:5 M), HCl/HNO_3_/H_2_O_2_ (3:1:1 M), HNO_3_/HCl/HF (1:3:2 M), and HF/HClO_4/_HCl (5:1:1 M) for extraction, while the most commonly used acid mixture was HNO_3_/HCl (1:3 M). An inductively coupled plasma–optical emission spectrometer (ICP-OES) or inductively coupled plasma–mass spectrometer (ICP-MS) and atomic adsorption spectrometer (AAS) were the widely used analytical instruments for analysis of extracted PTEs. On the other hand, XRF and EDXRF solid-phase PTE concentration analysis instruments have not been widely used.

The detection limit is the lowest amount of analytes in a sample that can be detected by an individual instrument. It is used to characterize the analytical method and instrument in terms of its ability to detect low levels of analytes and compare it to other methods, instruments, or standards. However, the detection limits of analyzed PTEs were not specified in most of the articles reviewed in this study. According to those reported detection limit values by 18 articles, it can be concluded that the detection limits of the ICP-MS analytical instrument were lower than those obtained by ICP-OES, ICP-AES, and AAS. For instance, the detection limits of AAS-cold vapor, ICP-AES, ICP-OES, and ICP-MS instruments for Pb were found to be 5, 82, 3, and <0.01 μg/L, respectively.

### 3.2 Concentrations

Descriptive statistics (mean, median, 25th–75th percentile, and 95th percentile) of the extracted concentrations of PTEs (Al, As, Cd, Co, Cr, Cu, Fe, Mn, Ni, Pb, Zn) compiled in this study are presented in Supporting Information 2-Sheet 1 grouped according to provinces, and in Table S1.2, they are grouped according to site characteristics. The locations of sampling sites are shown on a map for each PTE (Figures S1.1–S1.12). PTE concentrations except for Al, Fe, and Mn were compared to the limit levels found in Turkish soil pollution regulations. The mean concentrations of Al, As, Cd, Co, Cr, Cu, Fe, Mn, Ni, Pb, and Zn were found to be 21085, 188, 1.55, 13.9, 133, 72.9, 18918, 555, 89.2, 78.7, and 162 mg/kg in soil, respectively, for which the coefficient of variation (CV) values ranged from 0.83 to 3.85.

Al and Fe are two of the most abundant elements found in the Earth’s crust and major constituents of all soils. Therefore, the occurrence of Al and Fe in soil mainly related to natural factors except for places around hotspot anthropogenic sources. In this study, Al was the highest-concentration PTE with 25th, 75th, and 95th percentile values of 4075, 33232, and 58933 mg/kg, respectively. Soil Al concentrations in Turkey are in the range of those reported in the literature, which were conducted in Brazil,^39^ China,^40^ Japan,^41^ and Libya.^42^ Beattie et al.^43^ reported a topsoil average concentration of 1466 mg/kg in the town of Picher, Oklahoma, USA, which is in a mining district, while a topsoil mean concentration of 13800 mg/kg was reported for urban soil in metropolitan Bangkok.^44^ Fe followed Al with 1859, 27918, and 62458 mg/kg at 25th, 75th, and 95th percentiles, respectively. The Al and Fe concentrations were less variable, with CV values of 1.27 and 1.37, respectively, than the other PTEs except for Co with a CV of 0.83. The lower variation in Al and Fe concentrations could be explained by their crustal abundance. For instance, the Fe concentrations were also less variable within different types of sites with CV values of 1.15, 1.13, 0.51, 1.24, and 1.15 for urban, suburban, rural, industrial, and agricultural areas, respectively. Nevertheless, the Fe concentration variability was more obvious in industrial areas compared to rural probably due to its abundance in production and manufacture. Mn is another naturally occurring element that is found in soil and comprises about 0.1% of the Earth’s crust. The occurrence of Mn in soil is commonly related to natural activities, namely, forest fires, vegetation, volcanic activity, ocean spray, and weathering of Mn-containing minerals. The hotspot anthropogenic sources of Mn include mining and mineral processing; emissions from iron, steel, and alloy production; sewage sludge; and municipal wastewater discharges. In this study, Mn concentrations in Turkey were found to be 107, 744, and 1788 mg/kg at 25th, 75th, and 95th percentiles, respectively. Evident with the CV value of 1.07, it can also be concluded that the Mn concentration in soil mainly related to natural activities. There was also no significant variability in the Mn concentration (see CV values in Table S1.2) within site types except at agricultural areas with a CV of 1.38, which may indicate the effect of fertilizers along with the main natural sources.

On the other hand, the Cd, Cu, and Zn contents of soil mainly affected by anthropogenic sources, especially agricultural activities for the latter two (e.g., use of fertilizers and pesticides),^2,28^ probably resulting in spatial variation, are evident with CV values of 2.03, 2.61, and 3.37, respectively. Variation is more noticeable in the industrial and urban categories than that in rural (see CV values in Table S1.2). The Cd, Cu, and Zn 25th–95th percentile concentration ranges were found to be 0.14–6.97, 15.2–233, and 29.9–633 mg/kg, respectively. The mean concentrations of these three PTEs were lower than the regulation limit values in Turkey (1, 50, and 150 mg/kg, respectively), regardless of the location characteristic specified in Table S1.2. The mean concentrations of Cd, Cu, and Zn in this study are in the range of those reported in the literature.^45,46^ Lv and Liu^47^ identified sources and hazardous areas of heavy metals in the industrial city of Boshan, China. The mean soil concentrations of Cd, Cu, and Zn were reported to be 0.21, 33.4, and 87.3 mg/kg.^47^ The mean concentrations of Cd, Cu, and Zn in agricultural and forest topsoils were found to be 0.40, 16.5, and 69.8 mg/kg and 0.50, 18.8, and 83.3 mg/kg, respectively.^48^

However at lower levels than those presented above, the mean As, Ni, Pb, and Cr concentrations exceeded the Turkish regulation limits (20, 30, 50, and 100 mg/kg, respectively) by 9.41, 2.97, 1.58, and 1.34 times, respectively. The mean concentration of As was 2.50 mg/kg in rural areas, 6.98 mg/kg in urban areas, and 500 mg/kg in industrial areas, indicating that As mainly originated from anthropogenic sources. The variations of As concentrations were remarkable in industrial and suburban areas with the CV values of 2.08 and 1.52, which show the relevance of geogenic arsenic (see CV values in Table S1.2). Nevertheless, the mean As concentration of 2.5 mg/kg in rural areas is 2.8 times lower than that of urban areas (6.98 mg/kg). These results indicate that urbanization and industrialization have a significant effect on As contamination in Turkey. Abanuz (2011) studied heavy metal contamination of surface soil around Gebze industrial area, Turkey, and found that the As concentration was in the range of 1.5–65.6 mg/kg.^49^ A similar trend was observed for Ni, Pb, and Cr mean concentrations and concentration variations at different site characteristics. The mean concentrations of Ni, Pb, and Cr were higher in industrial and urban areas than in rural areas. In addition, the mean concentration of Co in industrial areas slightly exceeded the Turkish regulation limit of 20 mg/kg (by 1.06 times), while its mean concentrations were lower than the regulation limit value in rural, suburban, urban, and agricultural areas.

The mean concentrations of Al, As, Cd, Co, Cu, Pb, Zn, Fe, Mn, Cr, and Ni were calculated to be 29160, 6.98, 0.92, 12.4, 66.3, 35.0, 128, 20772, 341, 88.7, and 95.8 mg/kg in urban areas of Turkey (Table S1.2), which are lower than the maximum contaminant levels (MCLs) listed in Regulation on Control of Soil Pollution.^50^ Meanwhile, the mean concentrations of Al (35588), As (501), Cd (4.25), Co (21.1), Cu (588), Pb (248), Zn (249), Fe (35580), Mn (992), Cr (334), and Ni (126) mg/kg in industrial areas were considerably higher than those of urban, suburban, rural, and agricultural areas and exceeded their MCLs. The most prominent PTE is As with a mean concentration of 501 mg/kg, which is 25-fold the MCL of 20 mg/kg. The effect of industrial activities on soil PTE contamination is apparent with higher CV values (0.5–3.4) compared to those in urban, suburban, and rural sites (0.65–1.37, 0.62–1.52, and 0.45–1.08, respectively). Ranges of CV values for agricultural and noncategorized sites were 0.93–2.33 and 0.13–1.37, respectively, indicating that agriculture has also a remarkable effect on soil PTE contamination following the industry.

### 3.3 Exposure and Risk Assessment

In this study, exposure to PTEs in soil was solely assessed for the accidental ingestion of soil. PTE-associated chronic toxic risk (CTR) and carcinogenic risk (CR) levels were estimated based on deterministic and probabilistic approaches. PTE concentrations were taken from reviewed published articles which are presented in S2-Sheet 1. Individual PTE concentrations were used for the deterministic approach. However, PTE concentrations were fitted to a best-fitting probability distribution for the probabilistic approach (Table S1.3). PTE exposure and risk levels through accidental soil ingestion pathway were estimated for various age groups of females and males, and adults. Accidental ingestion rates, slope factors of the carcinogenic dose–response curves of PTEs for ingestion route, and reference dose levels are listed in Table S1.4. CTR was estimated for Al, As, Cd, Cr (III), Cr (VI), Cu, Co, Fe, Mn, Ni, and Zn, while the CR was estimated for As, Cr (VI), and Pb determined by the availability of chemical-specific reference dose and oral slope factor values. CR levels were evaluated in four categories:^51,52^ CR ≤ 10^–6^ considered as there is no risk (safe zone), 10^–6^ < CR < 10^–5^ considered as acceptable risk zone, 10^–5^ < CR < 10^–4^ considered as low priority risk zone, and CR ≥ 10^–4^ considered as unacceptable risk and high priority risk zone. Estimated PTE exposure levels via accidental ingestion of soil are presented in S2 – Sheets 2 and 3.

Spatial variation in exposure levels was analyzed by comparing locations categorized as urban, suburban, industrial, and agricultural using the Mann–Whitney U test at the significance level of 0.05. While Al exposure levels in urban, suburban, and industrial sites were similar, agricultural sites were significantly lower. Arsenic exposure levels were estimated to be higher in industrial sites, followed by suburban, urban, and agricultural sites. The Cd exposure levels were higher in industrial sites, similar in urban and suburban sites, and lower in agricultural sites. Cr(III) and Cr(VI) exposure levels in urban, suburban, and agricultural sites were not significantly different, while they were higher in industrial sites. Exposure levels of Co in urban and agricultural sites were similar, and those in industrial and suburban sites were similar, while the levels in the latter groups were higher than the former groups. Exposure levels of Cu in urban and industrial sites were similar and higher than those in suburban and agricultural sites. The exposure levels of the co-occurring elements Fe and Mn were not significantly different in urban, suburban, industrial, and agricultural sites. Ni exposure levels in industrial and suburban areas were not significantly different, while they were lower in urban and agricultural sites. While the Pb and Zn exposure levels were significantly higher in industrial areas, they were similar in urban and suburban areas and the lowest in agricultural sites. In summary, the Mann–Whitney U test results show that As, Cd, Cr(III), Cr(VI), Co, Cu, Ni, Pb, and Ni exposures through nondietary intake of surface soil are significantly affected by industrialization/urbanization, while Fe and Mn exposures were not affected.

#### 3.3.1 Chronic Toxic Risk

HQ is the unitless quantitative indicator of CTR. While the HQ values exceeding the threshold level (“1”) represent the exposures posing risk, values between 0.1 and 1 indicate a need for further investigation, and HQ<0.1 is conceived as insignificant.^53^ The lowest CTR levels of Al were estimated for adults due to the higher body weight and lower accidental soil intake rate. Average CTR levels of Al for female adults, male adults, and (combined) adults were estimated to be 5.40 × 10^–3^, 4.61 × 10^–3^, and 4.99 × 10^–3^, respectively. CTR levels of Al for females were estimated to be higher than that for males due to the lower body weight. Spatial variations of overall CTR levels (combined adults) of the accidental ingestion for all PTEs are presented in S-1, Figures S1.13 to S1.24. Descriptive statistics of CTR levels are shown in S-1, Table S1.5. Al was suspected for Alzheimer’s disease.^54^ The highest CTR levels of Al were determined for newborns (6 week–1 year) probably due to the relatively higher ingestion rate-to-body weight ratio than the other age groups. Average CTR of newborn girls, boys, and combined group were estimated 8.45 × 10^–2^, 7.32 × 10^–2^, and 7.46 × 10^–2^, respectively. The highest CTR level of Al was estimated to be in Giresun at a nonindustrial site. Higher Al concentrations due to the geological formation and agricultural activities might be the reason for the higher CTR levels. Namely, the main causes of soil Al contamination could be the origin of soils, showing that they evolved from volcanic activities and climatic conditions since rainfall produces leaching of Al from agricultural soil layers. Therefore, the Al contamination commonly found in the topsoil at a depth of up to 20 cm.^55^ Moreover, Al contamination was widely observed in arable land, which consists of acidic soils worldwide.^56^ The main reasons of soil acidification on agricultural fields are precipitation of H^+^ ions, input of acidifying gases from atmosphere, usage of ammonia and sulfur-based fertilizers, and mineralization of organic substances.^57^ Therefore, high Al concentrations in the topsoil of agricultural fields could be observed due to the acidic conditions with excess use of fertilizers. However, estimated CTR levels of Al were much lower than the threshold level of “1” for all age and sex groups and regions.

Arsenic is one of the most potent elements for human health. Both of the CTR and CR of arsenic were higher in males than those in females. Males being more susceptible to kidney damage, renal oxidative stress, and skin lesions might be due to the higher arsenic methylation rate and excretion of arsenicals in females.^58,59^ Natural occurrences of As in the earth crust show a variation depending on environmental geochemistry. Additional anthropogenic sources may increase the ambient background concentrations.^60^ Average CTR levels of As for adults are shown in Figure S1.14. CTR levels of As for female, male, and combined adults were estimated to be 1.61 × 10^–1^, 1.37 × 10^–1^, and 1.49 × 10^–1^, respectively, whereas the upper-bound (95th percentile) CTR levels for adults were estimated to be (0.85–0.99) near the threshold level of “1”. The highest CTR levels were for newborns with average values of 2.51, 2.18, and 2.22 for females, males, and combined group, respectively, exceeding the threshold. The average CTR levels of As for the 1–6 years olds was also >1, while they drop slightly below the threshold for 6–11 year olds. In summary, the average CTR levels for all age groups, except 0–1, were in the range of 0.1 to 1.0, indicating a need for further investigation.

While Cd rarely occurs in the earth crust, industrial and agricultural activities increase its soil levels.^61,62^ Overall CTR levels of Cd for combined-sex adult group are shown in Figure S1.15. Average CTR levels of Cd for adults were on the order of 10^–3^, while they were on the order of 10^–2^ for all other age groups except for newborns with the average of 1.26 × 10^–1^ for girls and 1.09 × 10^–1^ for boys. The orders of CTRs associated with Co were similar to those of Cd. As the humans are potentially exposed to Co with dietary supplements, Co alloys, and industrial activities,^63^ ingestion of the Co-ingredient soils could increase the exposure levels. Hokin et al.^64^ reported that the primary Co exposure pathway was dietary ingestion with 11–45 μg/day. Recently, a linear relationship between urinary Co and diabetes markers (FPG, HbA1c, insulin, and HOMA-IR) was observed in males while not in females.^65^

Copper smelters, agricultural use of Bordeaux mixture, and natural occurrence in the earth crust are the main sources of Cu in soils.^66,67^ Accidental ingestion of soils and house dust might be important contributors to Cu exposure. Fe is one of the most frequently found elements in the earth crust.^67^ Besides the natural occurrence, industrial production and smelter industries increase the Fe concentration of soil in the close areas. So, expected Fe exposure levels are generally higher than those for others. The average CTR values estimated for Cu and Fe ranged between the orders of 10^–4^ and 10^–1^ for the studied age groups. Extreme concentrations near the industrial sites, Corlu Organized Industrial Site in Tekirdag and Organized Industrial Site in Eskisehir, resulted in higher CTRs compared to other sites, rising maximum value to the threshold level, while the highest 95th percentile value was 3.66 × 10^–1^. In addition to industrial effects, relatively higher Fe concentrations in Igdir indicated that the geological effects might be important on Fe exposure at nonindustrial sites. Overall, CTRs of Mn were determined to be lower than the threshold level, even the maximum value. Cr concentrations in soil are generally fractioned to Cr(III) and Cr(VI) with about 80 and 20% of the total Cr, respectively.^68^ Even for the highest risk group, female newborns, the maximum CTR of Cr(III) was estimated to be lower than the threshold level (8.33 × 10^–3^), while that of Cr(VI) was at the threshold level (1.04). Ni exposure significantly decreases estrogen levels and causes sexual maturity in females.^69^ The average CTRs of Ni were also estimated to be lower than the threshold level even at the maximum (5.88 × 10^–1^).

#### 3.3.2 Carcinogenic Risk

**Figure 1:**
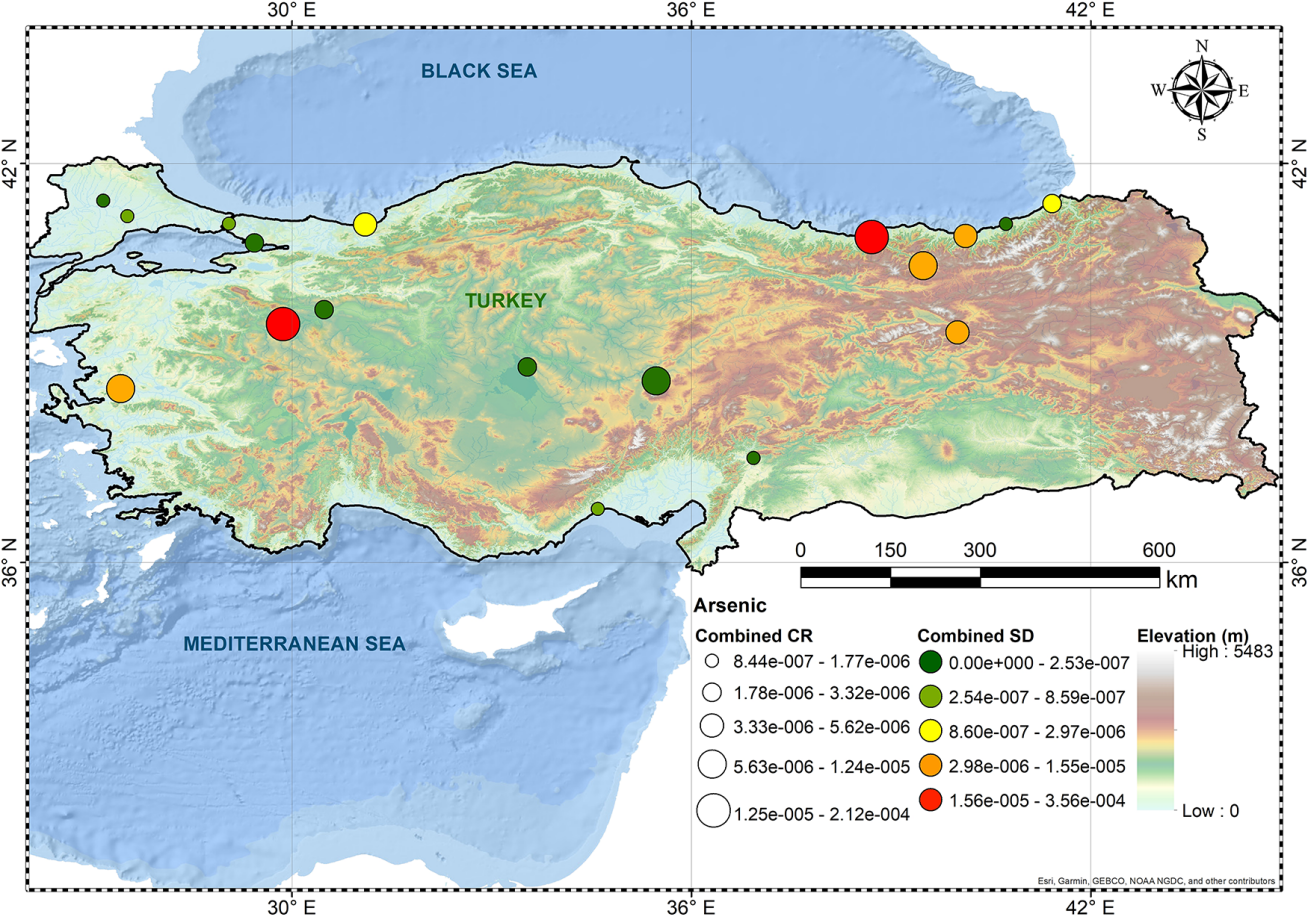
Overall CR levels of As.

**Figure 2:**
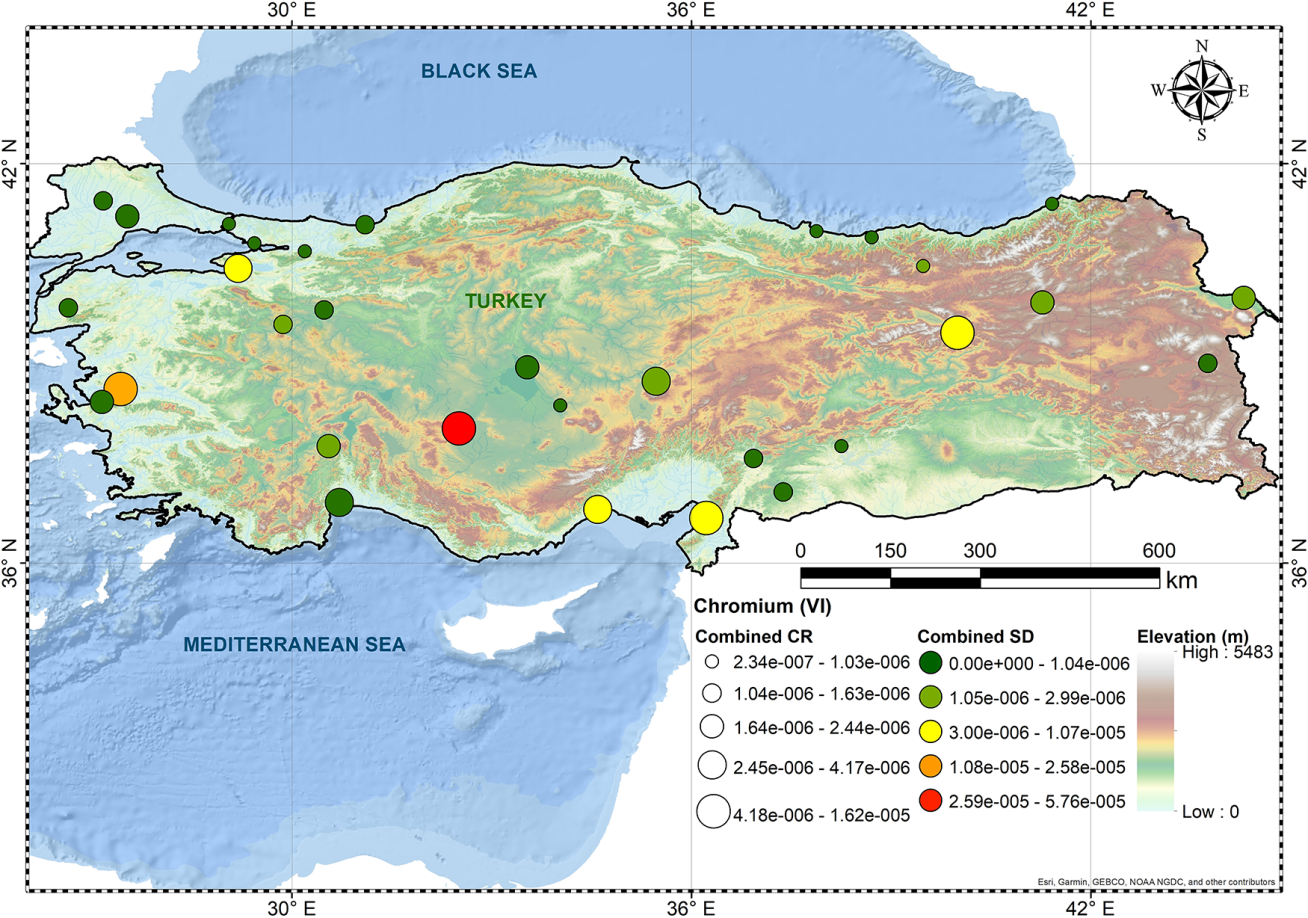
Overall CR levels of Cr(VI).

**Figure 3:**
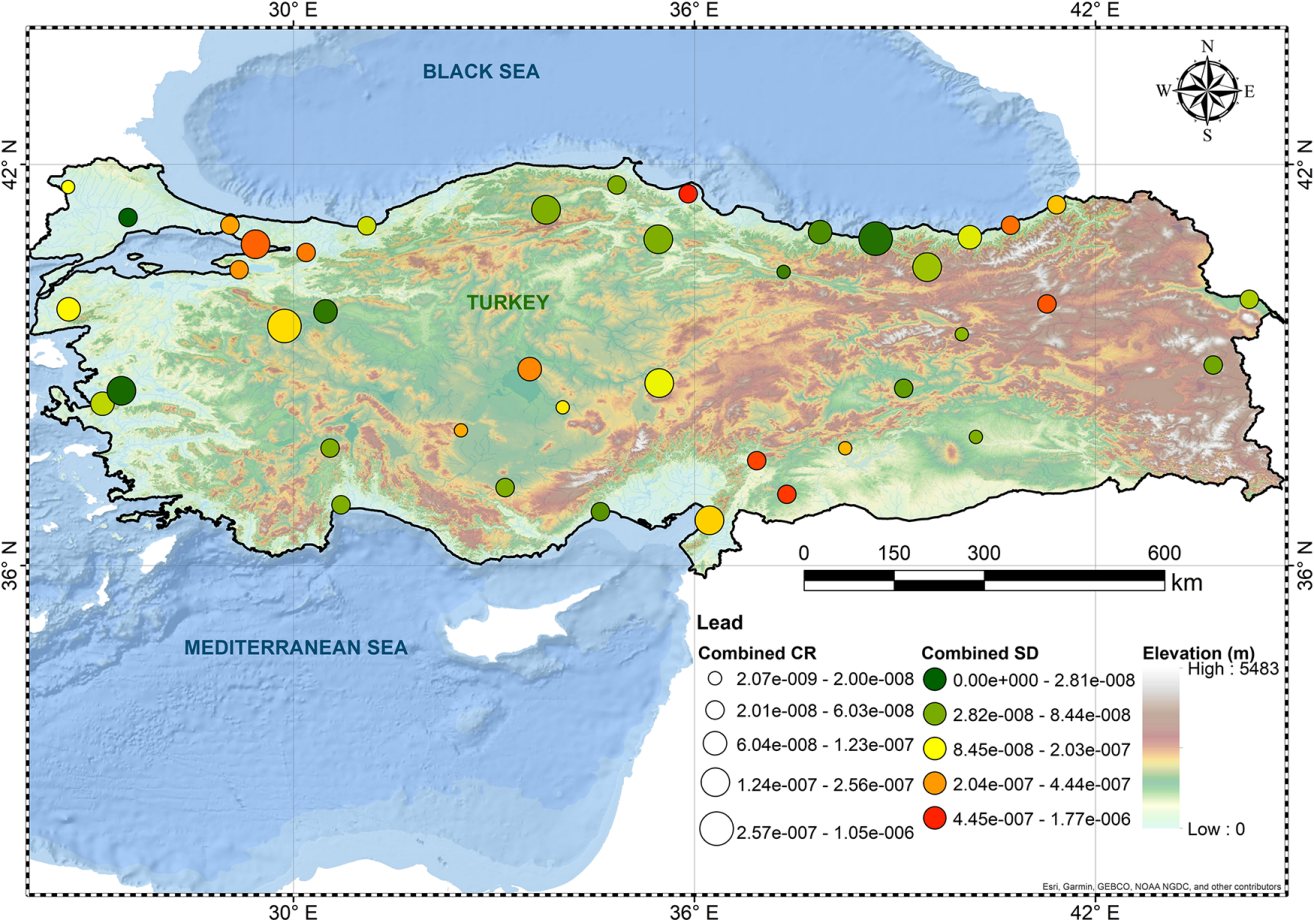
Overall CR levels of Pb.

Exposure to Cr(VI) contaminated drinking water causes hypomethylation of blood DNA, which increased the plasma oxidative biomarkers in male rats.^70^ Not only significant association between lung cancer and blood Cd levels were reported for male and females but also classified as kidney and breast carcinogen.^71,72^ While Pb exposure caused neurological and nervous system disorders in female zebrafish, genetic alterations (associated to cancer and tumor) occurred in males.^73^ Kidney cancer is one of the most important health effects of As.^58,59^ CR levels of As, Cr(VI), and Pb through accidental soil ingestion were estimated. The average CR levels of As for female, male, and combined-sex group adults were estimated to be 5.21 × 10^–5^, 4.45 × 10^–5^, and 4.81 × 10^–5^, respectively. All sex and age group average CR levels for As were in the low priority zone, except for 1–6 year olds with those in the high priority zone. There was a difference in the average and median levels, the former being in the low priority zone while the latter being in the safe zone. These findings indicated that there are small population groups with high As-associated CR while large population groups are in the safe CR areas. The highest CR levels of As were estimated to be in Kutahya, followed by Giresun and Gumushane. Figures 1–3 show spatial variation for As, Cr(VI), and Pb, respectively. CR levels of Cr(VI) up to the upper-bound estimates were in the acceptable risk zone. Pb-associated CR levels were the lowest in this study with both average and upper-bound risks being in the order of 10^–7^.

### 3.4 Probabilistic Risk Assessment

**Figure 4:**
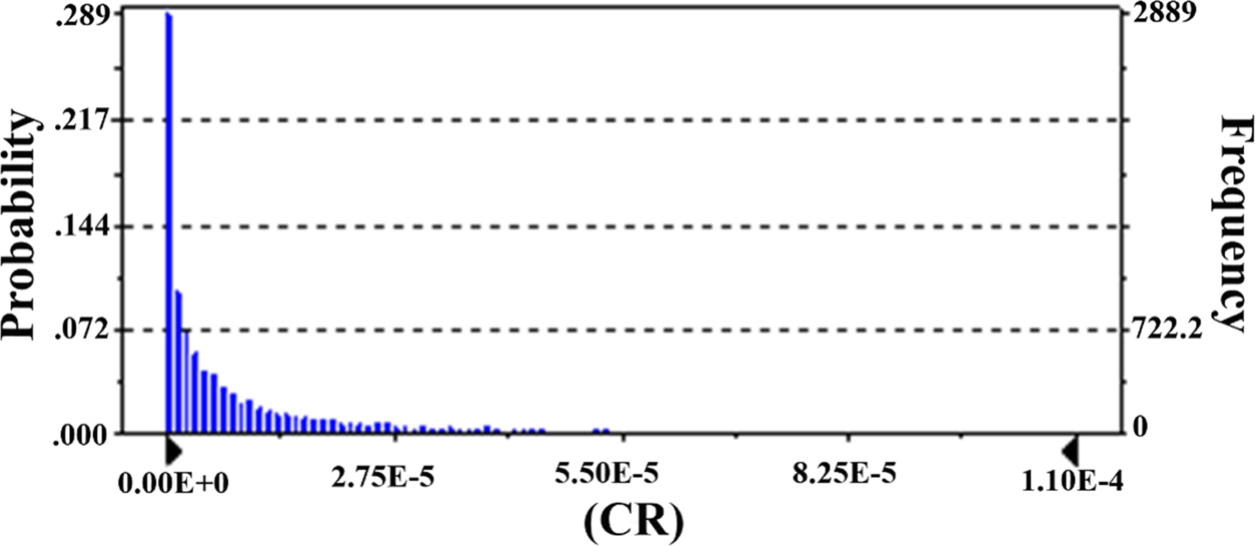
Cr(VI)-associated CR levels of Turkish population.

**Figure 5:**
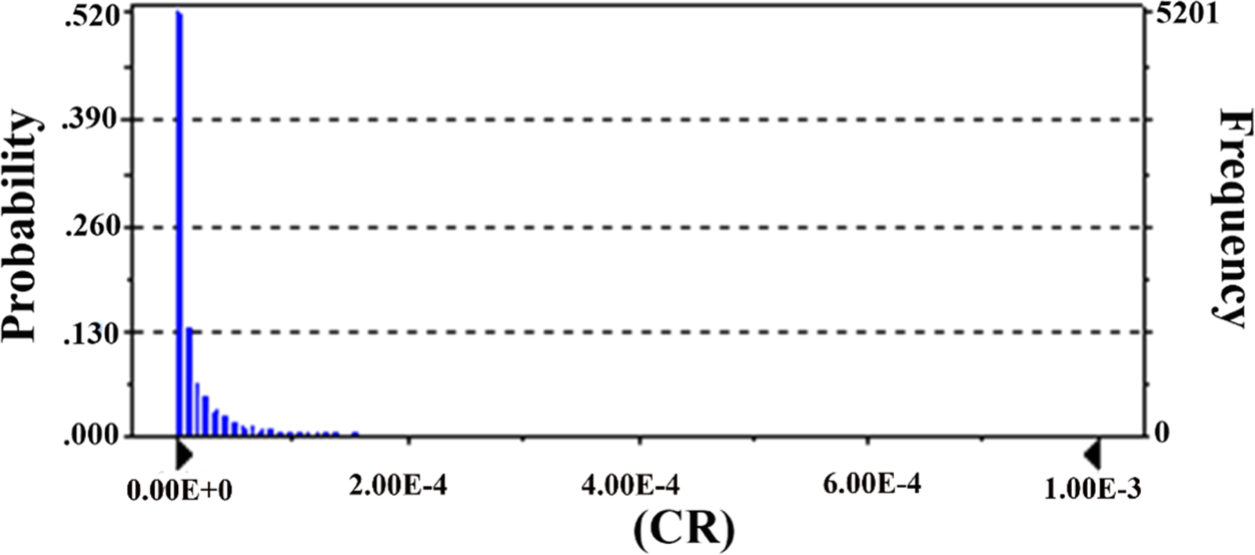
As-associated CR levels of Turkish population.

**Figure 6:**
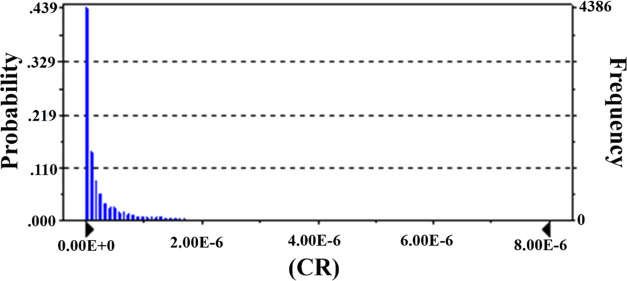
Pb-associated CR levels of Turkish population.

Soil concentrations of each PTE assessed in this study were fitted to a probability distribution. Parameters of the fitted probability distributions are presented in Table S1.3. CTR and CR were simulated with the Monte Carlo technique (10000 trials) to estimate parameter values of probability distributions for Turkish population (Figure S1.25–S1.59). The averages of simulated CTR levels of As, Cd, Co, Cr(III), Cr(VI), Cu, Fe, Mn, Ni, and Zn were 2.98 × 10^–1^, 6.95 × 10^–3^, 5.24 × 10^–2^, 7.64 × 10^–5^, 9.75 × 10^–3^, 1.94 × 10^–3^,5.32 × 10^–2^, 4.05 × 10^–3^,4.77 × 10^–3^, and 8.21 × 10^–4^, respectively. While the upper-bound CTR levels of PTEs were estimated to be lower than the threshold level, the maximum CTR level of As was higher than the threshold level. Interquartile ranges of the CTR levels of As, Cd, Co, Cr(III), Cr(VI), Cu, Fe, Mn, Ni, and Zn were estimated to be 1.02 × 10^–2^–1.56 × 10^–1^, 1.02 × 10^–4^–1.88 × 10^–3^, 1.28 × 10^–2^–6.26 × 10^–2^, 4.06 × 10^–6^–7.22 × 10^–5^, 5.22 × 10^–4^–9.35 × 10^–3^, 1.51 × 10^–4^–1.52 × 10^–3^, 8.69 × 10^–4^–4.38 × 10^–2^, 4.89 × 10^–4^–4.58 × 10^–3^, 3.10 × 10^–4^–4.85 × 10^–3^, and 3.49 × 10^–5^ −4.65 × 10^–4^, respectively. The CV was <0.1 for the studied PTEs except for Cd (0.37). While the average CR levels of As, Cr(VI), and Pb were estimated to be 1.81 × 10^–4^, 1.46 × 10^–5^, and 6.28 × 10^–7^, respectively, the upper-bound CR levels of these PTEs were 5.14 × 10^–4^, 6.23 × 10^–5^, and 2.34 × 10^–6^, respectively (Figures 4–6). CR levels of As, Cr(VI), and Pb were estimated to be in low priority zone, acceptable risk zone, and no risk zone, respectively. The CV of the estimated CR levels of As, Cr(VI), and Pb were 5.2, 2.3, and 4.5, respectively.

Uncertainties in the Monte Carlo simulation were determined using the bootstrapping method. The Monte Carlo simulation (*n* = 1000 trial) was repeated 200 times to estimate variation originating from the random value selection process. The mean and median levels of CTR and CR estimations were considered for uncertainty. While the standard errors (SEs) of the mean CTR levels of Al, As, Cd, Co, Cr(III), Cr(VI), Cu, Mn, Ni, and Zn were estimated to be 1.03 × 10^–4^, 4.54 × 10^–3^, 7.43 × 10^–5^, 1.61 × 10^–4^, 3.92 × 10^–7^, 5.06 × 10^–5^, 1.77 × 10^–5^, 1.76 × 10^–5^, 2.36 × 10^–5^, and 8.44 × 10^–6^ respectively; the SEs of the mean CR levels of As, Cr(VI), and Pb were 1.92 × 10^–6^, 7.05 × 10^–8^, 3.39 × 10^–9^, respectively. Uncertainties of the Monte Carlo simulation were also determined for median values of the CTR and CR levels. Interquartile ranges of the median CTR levels of Al, As, Cd, Co, Cr(III), Cr(VI), Cu, Mn, Ni, and Zn were estimated to be −7.30 × 10^–4^, 4.50 × 10^–3^, 5.10 × 10^–5^, 1.60 × 10^–3^, 2.30 × 10^–6^, 2.80 × 10^–4^, 4.40 × 10^–5^, 1.50 × 10^–4^, 1.70 × 10^–4^, and 1.10 × 10^–5^, respectively, while those of the median CR levels of As, Cr(VI), and Pb were 1.80 × 10^–6^, 5.00 × 10^–7^, and 1.40 × 10^–8^, respectively. Those ranges were 1 order of magnitude lower than the upper and lower levels of interquartile ranges. Bootstrapping shows the uncertainty of the CR and CTR models because random selection processes were low and, therefore, could not significantly affect the estimated risk levels.

Deterministic risk estimations of this study were based on point estimates of exposure variables (i.e., body weight and ingestion rate) for American people taken from the USEPA Exposure Factors Handbook. Probabilistic risk estimations, however, were based on a body weight probability distribution specific to Turkish people and ingestion rate probability distribution constructed from the values reported in the literature. As a result, considerable discrepancies (ranging from 6.57% for Cd (CTR) to 130% for Pb (CR)) occurred between the two types of estimations, indicating that assessments based on point estimates for other populations (nations) than those of the subject population may result in considerably strayed exposure–risk estimations.

## 4 Conclusions

Surface soil PTE concentrations in Turkey were reviewed and accidental ingestion route CTRs and CRs were estimated using deterministic and probabilistic approaches. Aluminum and iron were the most abundant PTEs in surface soil due to their abundance in the Earth’s crust. PTE concentrations at industrial sites were higher than those at other sites, which might be due to deposition of atmospheric particles with high PTE content emitted by industrial activities. Geogenic variation was also an important factor on the soil PTE concentrations such as 4-fold higher arsenic levels in Giresun and Kütahya than those in other locations in Turkey, resulting in considerable CTR and CR levels. While 1–6 year old children have higher CTR and CR risks, the two sexes have different levels in all age groups because of the lower female body weights than those of males. The estimated Turkish population upper-bound CTR levels were lower than the threshold level of unity, except for lower age groups (0–1 and 1–6) in some cases, indicating that care should be taken for subpopulations in public health mitigation efforts. Because indoor settled dusts are significantly correlated to the atmospheric particles and outdoor soils, relatively higher risk levels for children implicate that inclusion of PTE contamination in indoor dust, especially in homes, schools, kindergartens, and entertainment centers of children, would bring the risks to even higher levels, deeming accidental ingestion exposure an important pathway, and making cleaning in these built environments critical for the well-being of children.

## Supporting Information Available

Sampling, extraction, and analysis methods; studied PTEs; detection limits; descriptive statistics of the extracted concentrations of PTEs; CV values; PTE-associated chronic toxic risk (CTR) and carcinogenic risk (CR) levels; carcinogenic dose–response curves of PTEs for ingestion route and reference dose levels; CTR and CR simulations with the Monte Carlo technique (PDF)
PTE concentrations, average daily dose (ADD), and lifetime ADD dataset(XLSX)
