# Supplementary material for: Countrywide Spatial Variation of Potentially Toxic Element Contamination in Soils of Turkey and Assessment of Population Health Risks for Nondietary Ingestion
Source: ACS Omega. 2022 Oct 4;7(41):36457–67. doi: 10.1021/acsomega.2c04261 (PMC9583639; doi:10.1021/acsomega.2c04261)
Supplement: Supplementary file 1 — ao2c04261_si_001.pdf [file ao2c04261_si_001.pdf]

Supplementary Information 1

Country-wide spatial variation of potentially toxic element contamination in soils of Turkey and assessment of population health risks for non-dietary ingestion

Aysegul Yagmur Goren[1], Mesut Genisoglu[1], Yiğithan Kazancı[1], Sait C. Sofuoglu[1]*
[1]Izmir Institute of Technology, Dept. of Environmental Engineering,
Gulbahce, Urla 35430 Izmir, Turkey

*Corresponding author.

e-mail: cemilsofuoglu@iyte.edu.tr; saitcemil@iit.edu

Phone: +90-232-750 6648

Table S1.1. Extraction methods and detection limits of PTEs.

Sample	Analyzed PTEs	Extraction method, chemical, analytical instrument	Detection limit (µg/L)	Reference
Top soil, 0-5 cm	Pb, Cu, Zn, Fe, Co, Al, Cd	Ambient temperature extraction, 1:3 M HNO_3: HCl, ICP-MS	N.A.	[1]
Top soil, 0-30 cm	Fe, Mn, Zn, Cu, Co, Ni, Cr, Pb, Cd	DTPA extraction, 0.005 M DTPA, AAS	N.A.	[2]
Top soil, 0-30 cm	Fe, Cu	DTPA extraction, DTPA, AAS	N.A.	[3]
Top soil, 0-5 cm	Zn, Pb, Fe, Cu, Cr, Cd, As, Co, Mn	Microwave, 1:3 M HNO_3: HCl, ICP-OES	N.A.	[4]
Top soil, 0-30 cm	Fe, Cd, Cr, Cu, Zn, Pb, Ni	Microwave, 1:2:5 M $HClO_4$: HNO_3: HCl, AAS	N.A.	[5]
Top soil, 0-20 cm	Pb, Zn, Cd, Cu, Ni, Mn, Cr and Fe	Hot plate, H_2O_2+H_2SO_4+HF+HNO_3+$HClO_4$, FAAS and GFAAS	N.A.	[6]
Top soil, 10-25 cm	As, Cd, Co, Cu, Mn, Ni, Pb, Fe and Zn	Microwave, 1:3 M HNO_3: HCl, ICP-AES	As: 7.8; Cd: 3.3; Co: 5.2; Cu: 1.6; Mn: 0.5; Ni: 14.6; Pb: 81.8; Fe: 3.1; Zn: 1.7	[7]
Top soil, ~ 50 cm	Al, As, Cr, Zn, Mn, Cd, Co; Cu, Pb and Fe;	HNO_3, ICP-OES and ICP-MS	Al: 1; As: 0.5; Cr: 0.5; Zn: 0.5; Mn: 0.05; Cd: 0.05; Co: 0.02; Cu: 0.1; Pb: 0.1; Fe: 10; Ni: 0.2	[8]

Sample	Analyzed PTEs	Extraction method, chemical, analytical instrument	Detection limit (µg/L)	Reference
Top soil, 5-20 cm	Cd, Co, Cr, Cu, Pb, Zn, Mn, Fe, and Ni	HNO_3: H_2O_2, HCl, ICP-AES	N.A.	[9]
Top soil	Cd, Cr, Cu, Fe, Mn, Ni, Pb, and Zn	Microwave, 10 mL HNO3 (65%), ICP-OES.	Cd: 0.4; Cr: 0.5; Cu: 0.3; Fe: 0.4; Mn: 0.05; Ni: 1.3; Pb: 3; Zn: 0.3	[10]
Top soil, 0-15 cm	Cu, Pb, Zn, Ni, Ag, Co, Mn, Fe, As, Cd, Sb, Cr, and Al	ICP-MS	N.A.	[11]
Top soil, 0-10 cm	Al, Fe, Cr, As, Mn, Co, Sr, Cd, B, Cu, Pb, Zn, Ni and Hg	Ambient temperature extraction, 1:3 M HNO_3: HCl, ICP-OES	N.A.	[12]
Top soil, 0-10 cm	Fe, Mn, Mo, Ni, Co, Zn, Cd, Cu, Pb, Cr, and As	Ambient temperature extraction, 1:3 M HNO_3: HCl, ICP-MS	Zn: < 0.1 mg/L; Cd: < 0.01; Co: <0.1; Cu: <0.01; Pb: <0.01; Mo: <0.01; Ni: <0.1; Cr: <0.5	[13]
Top soil, 0-10 cm	Fe, Mn, Zn, Cu, Ni, Pb, Cr, Al and Cd	DTPA extraction and microwave, DTPA and 3:1:1 HCl:HNO_3:H_2O_2, ICP-OES	N.A.	[14]
Top soil, 0-3 cm	V, Cr, Ni, Cu, Zn, Pb	EDXRF	V: 2.9; Cr: 2.0; Ni: 1.2; Cu: 1.0; Zn: 0.7; Pb: 0.9 mg/kg	[15]
Top soil, 0-10 cm	Pb, Zn, Cd, Fe, and Cu	Hot plate, 1:3 M HNO_3: HCl, AAS	N.A.	[16]
Top soil	Cu, Zn, Fe, Cd, Cr, Pb, and Ni	SPME-GC/MS	N.A.	[17]
Top soil, 0-20 cm	Mn, Ni, Cr, Zn, Cu, Pb, Co and Cd	Modified BCR sequential extraction method, FAAS	N.A.	[18]
Top soil, 0-20 cm	Cd, Cr, Cu, Ni, Pb and Zn	Modified BCR sequential extraction method, FAAS	N.A.	[19]
Top soil, 0-10 cm	Ba, Cd, Cr, Cu, Li, Ni, Pb, Se, Sr, and Zn	Microwave, 1:3 M HNO_3: HCl , ICP-AES	N.A.	[20]
Top soil, 0-10 cm	Pb, Cd, Cu and Zn	Hot plate, 1:3 M HNO_3: HCl, AAS	N.A.	[21]

Sample	Analyzed PTEs	Extraction method, chemical, analytical instrument	Detection limit (µg/L)	Reference
Top soil, 0-30 cm	Cr, Fe, Cu, Zn, and Mn	DTPA extraction, DTPA, ICP-OES	N.A.	[22]
Top soil, upper 15 cm	V, Cr, Mn, Fe, Co, Ni, Cu, Zn, As, Cd, Sn, Sb, Ba and Pb	Microwave, 6:2:1:1 M HCl: HNO_3: H_2O_2: HF, ICP-MS	N.A.	[23]
Top soil, 0-20 cm	Cu, Ni, Zn and Pb	Modified BCR sequential extraction method, AAS	N.A.	[24]
Top soil, 20-30 cm	Se, Pb, As, Mo, Bi, Cd, Cu, V, Ba, Zr, Zn, U, Th, Ni, Co, and Sb	Hot plate, 2:2:2 HCl: HNO_3: H_2O , ICP-AES and ICP-MS	N.A.	[25]
Top soil, 0-20 cm	As, Cd, Co, Cr, Cu, Mn, Mo, Ni, Pb, V and Zn	BCR Sequential extraction method, ICP-MS	N.A.	[26]
Top soil, 5-10 cm	Ti, V, Cr, Mn, Ni, Cu, Zn and Pb	EDXRF	N.A.	[27]
Surface soil	Ba, Cd, Co, Cr, Cu, Fe, Mn, Ni, Pb, Sr, V, and Zn	BCR Sequential extraction method, ICP-OES	N.A.	[28]
Surface soil	Pb, Cu, Zn, Co, Cr, V, Cd and Ni	Microwave, ICP-AES	N.A.	[29]
Surface soil	Cd, Co, Cr, Cu, Fe, Mn, Pb, and Zn	Ambient temperature extraction, 1:3 M HNO_3: HCl, AAS	N.A.	[30]

Sample	Analyzed PTEs	Extraction method, chemical, analytical instrument	Detection limit (µg/L)	Reference
Top soil, 5-10 cm	La, Ce, Pr, Nd, Sm, Eu, Gd, B, Rb, V, Yb, Li, S, P, Si, Na, K, Ti, Cr, Mn, Ni, Cu, Zn, As, Zr, Y, Sn, Sr, Te, Sc, Al, Mg, Ca and Fe	ICP-AES	N.A.	[31]
Top soil, 0-5 cm	Pb, Cd and Cu	Hot plate, 2:1 HNO_3:H_2O_2 + 2 ml of 0.75 mol l^{-1} HNO_3, STAT-FAAS and FAAS	N.A.	[32]
Top soil, ~ 10 cm	Al, B, Ca, Cd, Co, Cr, Cu, Fe, K, Mg, Na, Ni, Pb and Zn	Microwave, 1:3:2 M HNO_3: HCl: HF, ICP-OES	N.A.	[33]
Top soil, 0-25 cm; 25-50 cm; 50-75 cm	Zn, Pb, Cd, Cu, Cr, Hg and Ni	Microwave, 19:1 HNO_3: HCl, FAAS and AAS-cold vapor system	Pb: 5; Cr: 5; Cd: 1; Cu: 20; Ni: 20; Zn: 0.25; Hg: 2.7	[34]
Top soil, 0-30; 30-60; 60-90; and 90 -120 cm	Cu, Zn, Mn, and Fe	N.A.-	N.A.	[35]
Top soil, 0-30 cm; 30-60 cm	Fe, Mn, Zn, Cu, Pb, Ni and Cd	DTPA extraction methods, AAS	N.A.	[36]
Surface soil	Co, Cd, Cu, and Fe	Microwave, 3:1:1: HCl: HNO_3: HF, ICP-OES	N.A.	[37]
Surface soil	V, Cr, Mn, Fe, Ni, Cu, Zn, Hg, and Pb	EDXRF	V: 2.9 mg/kg; Cr: 2.0; Mn: 2.5; Fe: 1.1; Ni:1.2; Cu: 1.0; Zn: 0.7; Hg: 1.6; Pb: 0.9	[38]
Top soil, ~ 50 cm	Ag, As, Ba, Cd, Ce, Co, Cr, Cs, Cu, Ga, Hg, La, Mo, Ni, Pb, Rb, Sb, Se, Sn, Sr, Th, U, V, W, Y, Zn, and Zr	HNO_3, ICP-OES and ICP-MS	N.A.	[39]

Sample	Analyzed PTEs	Extraction method, chemical, analytical instrument	Detection limit (µg/L)	Reference
Top soil, 0-35 cm	Fe, Zn, Cu, Al, Mn, Cd, Co, Ni, Pb, and Cr	Microwave, 3:1 HCl: HNO_3, ICP-AES	Fe: 1.6; Zn: 1.2; Cu: 1.0; Al: 5.9; Mn: 1.9; Cd: 1.1; Co: 2.1; Ni: 1.7; Pb: 4.2; Cr: 2.6	[40]
Top soil, 0-10 cm	Cu, Pb, Ni, Al, Cr, and Cd	Microwave, 3:1 HCl: $HNO_{3,}$ ICP-OES	N.A.	[41]
Top soil, 0-25 cm	Fe, Zn, Cu, Mn, and Cd	DTPA extraction, ICP-AES	N.A.	[42]
Top soil, 0-2 cm	As, Cu, Ni, and Cr	Microwave, 3:1 HNO3: HF, ICP-MS	N.A.	[43]
-	Pb, Cr, Cu, Mn, Ni, Zn and Cd	Hot plate, 3:1 HCl: HNO_3, ICP-OES	N.A.	[44]
Top soil, 0-25 cm	Cd, Co, Cr, Cu, Fe, Mn, Mo, Ni, Pb, and Zn	Ambient temperature extraction,4:1 HNO3: HCl, ICP-AES	N.A.	[45]
Top soil	V, Cr, Mn, Fe, Ni, Cu, Zn, As, and Pb, Sn, Ce, Hg	EDXRF	V: 2.9; Cr: 2.0; Mn: 2.5; Fe: 1.1; Ni: 1.2; Cu: 1.0; Zn: 0.7; As: 0.4; Pb: 0.9; Sn: 6.1; Ce: 7.8; Hg: 1.6 mg/kg	[46]
Top soil	V, Cr, Mn, Fe, Ni, Cu, Zn, Hg and Pb	EDXRF	V: 2.9; Cr: 2.0; Mn: 2.5; Fe: 1.1; Ni: 1.2; Cu: 1.0; Zn: 0.7; Pb: 0.9; Sn: 6.1; Hg: 1.6 mg/kg	[47]
Top soil, 5 cm	Cd, Cr, Cu, Ni, Pb, Zn, Al and Fe	Microwave, 10 mL HNO3 (65%), ICP-AES	N.A.	[48]
Top soil, 0-5 cm	Ba, Ni, Mo, Cu, Pb, Zn, Co, Mn, As, U, Sr, Cd, Sb, Bi, Cr, B, W, Hg, Sn, Li	Microwave, 10 ml $HClO_4$: HNO_3: HCl: HF, ICP-MS	N.A.	[49]
Top soil, 0-20 cm	As, Cd, Cr, Cu, Hg, Ni, Pb, and Zn	Microwave, 3:1 HNO_3: HCl, ICP-MS	N.A.	[50]
Top soil, 25 cm	Cu, Zn, Cd, and Mn	Hot plate, 2:2:2 HCl: HNO_3: H_2O solution, ICP-MS	N.A.	[51]

Sample	Analyzed PTEs	Extraction method, chemical, analytical instrument	Detection limit (µg/L)	Reference
Top soil, 5-10 cm	As, Cd, Cr, Cu, Hg, Mn, Pb, and Zn	Hot plate, 2:2:2 HCl: HNO_3: H_2O solution, ICP-MS	N.A.	[52]
Top soil, 10 cm	Cd, Cr, Cu, Mn, Ni, Pb and Zn	Microwave, 10 ml HNO_3 (65%), ICP-OES	N.A.	[53]
Surface soil	Ag, As, Pb, Zn, Sb, Cu, Sr, Cd, and Hg	ICP-MS	0.05 mg/L	[54]
Top soil, 0-10 cm	Co, Cr, Zn, Pb, Cd, Mn, Cu and Ni	Microwave, 10:5:5:3 HCl: HNO_3: HF: $HClO_4$, ICP-OES	N.A.	[55]
Top soil, 0-10 cm	Mg, Fe, Al, K, Cr, Cl, Cd, As, W, Ni, Pb, Co, Ag, Mo, Cu, Hg, Zn, Sb, V, Mn, Ti and Sn	EDXRF	N.A.	[56]
Top soil, 0-20 cm	Cr, Ni, Cd, Cu, Pb, and Zn	BCR sequential extraction method and hot plate, 3:1 HNO_3: HCl, FAAS	Cr: 0.02; Ni: 0.03; Cd: 0.002; Cu: 0.03; Pb: 0.02; Zn: 0.03 mg/kg	[57]
Top soil, 0-25 cm	Co, Cr, Cu, Fe, Mn, Mo, Ni, and Zn	Hot plate, 3:1 HNO_3: $HClO_4$, ICP-AES	N.A.	[58]
Top soil, 0-2 cm; 20 cm	Pb, Zn, and Cu	Microwave, 3:1 HCl: HNO_3, FAAS	1.5	[59]
Top soil, 5 -15 cm	As, Br, Ca, Cl, Cr, Fe, Hf, K, Sb, Sc, Sr, and Th.	INAA	N.A.	[60]
Top soil, 0 -5 cm	Al, Ba, Ca, Cd, Co, Cr, Cu, Fe, K, Mg, Mn, Na, Ni, Pb, Sr, V, and Zn	Hot plate, 5 mL 3:1 HCl: HNO_3 + 1 mL HF, ICP-OES	N.A.	[61]
Top soil, 0 -20 cm	Cd, Cu, Mn, Ni, Pb and Zn	Modified BCR sequential extraction method and hot plate, 3:1 HNO_3: HCl, FAAS	N.A.	[62]
-	Cr, Cu, Ni, Co, Cd, and Zn	Ambient temperature extraction, 10 mL HNO_3 (65%), ICP-AES	N.A.	[63]

Sample	Analyzed PTEs	Extraction method, chemical, analytical instrument	Detection limit (µg/L)	Reference
-	B, Cr, Cu, Fe, Pb, Li, Al, Ni, Co, Ba, Mn, Sr, Cd, Na, Zn and Tl	Ambient temperature extraction, 3:1 HCl: HNO_3, ICP-OES	N.A.	[64]
Top soil, 0 -5 cm	Cu, Cr and As	XRF	N.A.	[65]
Top soil, 0 -5 cm	Cu, Pb, Zn, Ni, Cr, Cd and Co	Hot plate, HNO_3: HF: $HClO_4$, AAS	N.A.	[66]
Top soil, 0-30 cm	Fe, Cu, Mn, and Zn	DTPA extraction methods, AAS	N.A.	[67]
-	Al, B, Cd, Co, Cr, Cu, Fe, Mn, Ni, Pb, Sn and Zn	Hot plate, 4:1:2 HNO_3: $HClO_4$: HCl, ICP-OES	N.A.	[68]
Top soil, 15 cm	As, Cr, Cd, Co, Cu, Mn, Ni, Pb, Zn, Fe, Mg	Microwave, HNO_3 or HNO_3 and HCl, ICP-OES	As: 0.06; Cr: 0.5; Cd: 0.4; Co: 0.3; Cu: 0.3; Mn: 0.05; Ni: 1.3; Pb: 3; Zn: 0.3; Fe: 0.4; Mg: 0.02	[69]
Top soil, 5 -10 cm	Ag, Cd, Mo, Fe, Al, Hg, Sb, Cr, W, Sn, Cu, As, Pb, Cl, Co and Zn	EDXRF	N.A.	[70]
Top soil, 5 cm	Al, Ca, Cr, Cu, Fe, Mg, Mn, Zn, Na, K, Pb, Ni and Cd	Hot plate, 4:1.5 HNO_3: HF, AAS	N.A.	[71]
Surface soil	As, Ag, and Pb	Hot plate, 1:1:1 HNO_3: HCl: H_2O, ICP-MS	N.A.	[72]
Top soil, 30 cm	Fe, Cu, Zn, and Mn	DTPA extraction, ICP-OES	N.A.	[22]
Top soil, 10-20 cm	Pb, Cd, Ni and Zn	DTPA extraction, ICP-OES	N.A.	[73]
Surface soil	As, Ag, Hg, Co, Cd, Cr, Pb, Ni, Ba, Zn, Cu, Se, Sb, Zr and U	ICP-MS	N.A.	[74]

Sample	Analyzed PTEs	Extraction method, chemical, analytical instrument	Detection limit (µg/L)	Reference
Surface soil	Ba, Co, Rb, Sr, Sc, Hf , Nb, Th, U, V, Zr, Y, La, Ce, Pr, Nd, Sm, Eu, Gd, Tb, Dy, Ho, Er, Tm, Yb, Lu, Ni , Mo; Cu, Pb, Zn, As; Cd, Sb, Bi, Ag , Au, Hg, Se	ICP-OES and ICP-MS	Ba: 1 mg/L; Co: 0.1; Rb: 0.1; Sr: 0.2; Sc: 0.5; Hf: 0.1; Nb: 0.1; Th: 0.2; U: 0.1; V: 8; Zr: 0.1; Y: 0.1; La: 0.1; Ce: 0.1; Pr: 0.02; Nd: 0.3; Sm: 0.05; Eu: 0.02; Gd: 0.05; Tb: 0.01; Dy: 0.05; Ho: 0.02; Er: 0.03; Tm: 0.01; Yb: 0.05; Lu: 0.01; Ni: 0.1; Mo: 0.1; Cu: 0.1; Pb: 0.1; Zn: 1; As: 0.1; Cd: 1; Sb: 0.1; Bi: 0.1; Ag: 0.1; Au: 0.5; Hg: 0.1; Se: 0.5	[75]
Top soil, 0-30 cm; 30-60 cm; 60-90 cm	Cd, Co, Cr, Cu, Mn, Ni, Pb, Zn	Hot plate, 5:1:1 1 HNO_3: H_2SO_4: $HClO_4$, ICP-OES	N.A.	[76]
Surface soil	Al, Ca, Mg, K, Zn, Pb, Cu, Ni, Co, Cr, V, Cd, Na	Microwave, 9:3:2 HNO_3: HCl: HF, ICP-OES	N.A.	[77]
Surface soil	Fe, Mn, Cu, As, Pb, Zn, Cr, Co, and Ba	ICP-MS	N.A.	[78]
Surface soil	Na, Mg, Al, Si, P, S, K, Ca, Ti, Fe, Cu, Zn, Sr, Cr, Mn, Rb and Pb	Skyray EDX 3600B spectrometer equipped with an Oxford Rh anode X-ray tube.	N.A.	[79]
Top soil, 0-5 cm; 5-20 cm	Cd, Cr, Co, Cu, Fe, Mn, Ni, Pb, and Zn	DTPA extraction, 0.005 M DTPA, ICP-AES	N.A.	[80]
Top soil, 0-20 cm; 20-40 cm	Cd, Co, Ni, and Pb	Ambient temperature extraction, HNO_3: HCl: H_2O_2, ICP-AES	N.A.	[81]
Top soil, 0-20 cm	Cd, Co, Cu, Ni, Pb, Zn,	Hot plate, 14:12 HCl: HNO_3, AAS	N.A.	[82]
Top soil, 0–5; 5–15; 15–30 cm	Pb	DTPA extraction, 0.005 M DTPA + 0.01M CaCl2 + 0.1M TEA), ICP	N.A.	[83]

Sample	Analyzed PTEs	Extraction method, chemical, analytical instrument	Detection limit (µg/L)	Reference
Surface soil	Fe, Cu, Zn, Mn, Ni, Pb, Cr, and Cd	AAS	N.A.	[84]
Top soil, 0-20 cm	Cr	Liberty Varien Model ICP-OES	N.A.	[85]
Top soil, 0-5 cm	Cd, Cr, Ni, Pb, Fe, Cu, Mn and Zn	Ambient temperature extraction, 2:1 HNO_3: $HClO_4$, GAAS	N.A.	[86]
Top soil, 10 cm	Cd, Cr, Cu, Ni, and Pb	Ambient temperature extraction, 3:1 HCl: HNO_3, ICP-MS	Cd: 0.01 mg/L; Cr: 0.5; Cu: 0.01; Ni: 0.1; Pb: 0.01	[87]
Top soil, 0-5 cm	Cd, Cr, Cu, Fe, Ni, Pb and Zn	Microwave, 10 ml HNO_3 (65%), ICP-OES	Cd: 0.0003 mg/kg; Cr: 0.0003; Cu: 0.0005; Fe: 0.0002; Ni: 0.0008; Pb: 0.002; Zn: 0.0002	[88]

* DTPA: Diethylene-Triamin-Pentaacetic Acid; ICP-MS: Inductively Coupled Plasma- Mass Spectrometer; AAS: Atomic Adsorption Spectrometer; ICP-OES: Inductively Coupled Plasma- Optical Emission Spectrometer; FAAS: Flame Atomic Adsorption Spectrometer; GAAS: Graphite Atomic Adsorption Spectrometer; EDXRF: Energy Dispersive X-ray Fluorescence Spectrometry; SPME-GC/MS: Solid Phase Microextraction-Gas Chromatography/Mass Spectrometry; STAT: Slotted Tube Atom Trap MP-AES: Microwave Plasma-Atomic Emission Spectrometer; INAA: Instrumental Neutron Activation Analysis; XRF: X-ray Fluorescence Spectrometry; N.A.: Not available.

Table S1.2. Site characteristic depended PTE concentrations of soil (mg/kg; mean±SD (CV)).

PTE	Urban	Sub-urban	Rural	Industrial	Agricultural	Non-categorized
Aluminum	29160 ± 20533 (0.70)	16414 ± 2193 (0.13)	63700	35588 ± 38998 (1.09)	5007 ± 11689 (2.33)	-
Cadmium	0.92 ± 1.12 (1.22)	0.78 ± 0.87 (1.12)	0.61 ± 0.64 (1.05)	4.25 ± 5.45 (1.28)	0.39 ± 0.51 (1.31)	8.26 ± 6.06
Cobalt	12.38 ± 9.76 (0.79)	19.6 ± 14.7 (0.75)	8.07 ± 0.68 (0.08)	21.1 ± 10.6 (0.5)	10.7 ± 10.0 (0.93)	34.1
Copper	66.3 ± 90.7 (1.37)	30.2 ± 24.0 (0.79)	16.6 ± 7.53 (0.45)	588 ± 2001 (3.40)	24.5 ± 25.6 (1.04)	31.5 ± 10.1
Lead	35.0 ± 47.6 (1.36)	28.4 ± 35.7 (1.26)	17.3 ± 15.5 (0.90)	248 ± 594 (2.40)	13.4 ± 21.3 (1.59)	14.4 ± 12.6
Zinc	128 ± 145 (1.13)	76.5 ± 47.5 (0.62)	67.8 ± 73.5 (1.08)	248 ± 328 (1.32)	58.2 ± 109 (1.87)	111 ± 151
Iron	20772 ± 23821 (1.15)	14043 ± 15911 (1.13)	15901 ± 8065 (0.51)	35579 ± 44233 (1.24)	9288 ± 10722 (1.15)	29734 ± 3850
Manganese	341± 245 (0.72)	534 ± 533 (1.00)	382 ± 393 (1.03)	992 ± 743 (0.75)	487 ± 673 (1.38)	754 ± 182
Chromium	88.6 ± 98.8 (1.12)	86.1 ± 83.6 (0.97)	41.2 ± 17.6 (0.43)	333 ± 811(2.46)	83.9 ± 163 (1.94)	42.5 ± 19.4
Nickel	95.8 ± 62.6 (0.65)	80.3 ± 74.6 (0.93)	48.0 ± 18.4 (0.38)	125 ± 238 (1.90)	65.8 ± 151(2.29)	153 ± 150
Arsenic	6.98 ± 4.76 (0.68)	33.6 ± 51.2 (1.52)	2.5	500 ± 1042 (2.08)	26.7 ± 30.4 (1.13)	-

Table S1.3. PTE concentration distributions of surface soil in countrywide Turkey.

PTE	Distribution	Parameters
Aluminum	Gamma	Location: 420.9, Scale: 61.75, Shape: 0.26
Cadmium	Log-normal	Location: 0.01, Mean: 2.65, SD: 13.68
Cobalt	Gamma	Location: 0.06, Scale: 22.01, Shape: 0.6
Copper	Log-normal	Location: 0.00, Mean: 44.2, SD:77.53
Lead	Log-normal	Location: 0.01, Mean: 42.68, SD: 135.91
Zinc	Gamma	Location: 0.08, Scale: 154.98, Shape: 0.56
Iron	Gamma	Location: 0.06, Scale: 35007.33, Shape: 0.27
Manganese	Gamma	Location: 1.72, Scale: 670.63, Shape: 0.52
Chromium (Total)	Log-normal	Location: -4.08, Mean: 82.71, SD: 147.93
Chromium (VI)	Gamma	Location: 0.00, Scale: 62.95, Shape: 0.43
Chromium (III)	Log-normal	Location: -3.16, Mean: 97.6, SD: 193.5
Nickel	Gamma	Location: 0.03, Scale: 142.44, Shape: 0.49
Arsenic (Total)	Log-normal	Location: 2.49, Mean: 53.39, SD: 646.17

Table S1.4. Model input parameters of exposure and risk assessment.

Parameter					Reference
C_{PTE}		Table SM 1.1. and SM 2			
IR (mg/day)	**Deterministic**				[89]
	6 weeks to <1 year	30			
	1 to <6 year	50			
	6 to <11 year	50			
	11 to <16 year	50			
	16 to <21 year	50			
	21 to <75 year	20			
	**Probabilistic**				
	Lognormal distribution	Loc. 0, Median:50, Upper percentile:200			
BW (kg)	**Deterministic**	**Female**	**Male**	**Combined**	[89]
	6 weeks to <1 year	7.18	8.29	8.13	
	1 to <6 year	15.4	15.8	15.6	
	6 to <11 year	31.7	31.9	31.8	
	11 to <16 year	55.9	57.6	56.8	
	16 to <21 year	65.9	77.3	71.6	
	21 to <75 year	74.9	87.6	81.0	
	**Probabilistic**				
	Beta distribution	Min. 0, Max. 11.15, Alpha 12.76, Beta 8.15			[90]
ED (year)	**Deterministic**				Assumed
	6 weeks to <1 year	0.875			
	1 to <6 year	5			
	6 to <11 year	5			
	11 to <16 year	5			
	16 to <21 year	5			
	21 to <75 year	59			
	**Probabilistic**				
	Lifespan	75			
EF (day/year)	**Both**	350			Assumed
RfD (mg/kg-day)	Aluminum*	1.00×10^{0}			[91]
	Arsenic	3.00×10^{-4}			[92]*
	Cadmium	1.00×10^{-3}			
	Chromium (III)	1.50×10^{0}			
	Chromium (VI)	3.00×10^{-3}			
	Cobalt	3.00×10^{-4}			
	Copper	4.00×10^{-2}			
	Iron	7.00×10^{-1}			
	Manganese	1.40×10^{-1}			
	Nickel	2.00×10^{-2}			
	Zinc	3.00×10^{-1}			
SF (mg/kg-day $^{-1}$)	Arsenic	1.50×10^{0}			[91]
	Chromium (VI)	5.00×10^{-1}			
	Lead	8.50×10^{-3}			

Table S1.5. Descriptive statistics of CTR and CR levels of Turkish population.

**Statistics:**	**As(CTR)**	**Cd (CTR)**	**Co CTR)**	**(CrIII) (CTR)**	**Cr(VI) (CTR)**	**Cu (CTR)**	**Fe (CTR)**	**Mn (CTR)**	**Ni (CTR)**	**Zn (CTR)**	**Cr(VI) (CR)**	**As (CR)**	**Pb (Cr)**
Trials	10000	10000	10000	10000	10000	10000	10000	10000	10000	10000	10000	10000	10000
Mean	2.98×10^{-1}	6.95×10^{-3}	5.24×10^{-2}	7.64×10^{-5}	9.75×10^{-3}	1.94×10^{-3}	5.32×10^{-2}	4.05×10^{-3}	4.77×10^{-3}	8.21×10^{-4}	1.46×10^{-5}	1.34×10^{-4}	6.28×10^{-7}
Median	4.02×10^{-2}	4.51×10^{-4}	2.92×10^{-2}	2.06×10^{-5}	2.72×10^{-3}	4.78×10^{-4}	8.85×10^{-3}	1.64×10^{-3}	1.42×10^{-3}	1.25×10^{-4}	4.08×10^{-6}	1.81×10^{-5}	1.07×10^{-7}
Standard Deviation	1.54×10^{-0}	2.59×10^{-1}	7.59×10^{-2}	1.78×10^{-4}	2.24×10^{-2}	6.11×10^{-3}	1.42×10^{-1}	7.03×10^{-3}	1.04×10^{-2}	5.48×10^{-3}	3.36×10^{-5}	6.91×10^{-4}	2.80×10^{-6}
Variance	2.36×10^{-0}	6.70×10^{-2}	5.77×10^{-3}	3.15×10^{-8}	5.03×10^{-4}	3.73×10^{-5}	2.01×10^{-2}	4.94×10^{-5}	1.09×10^{-4}	3.00×10^{-5}	1.13×10^{-9}	4.77×10^{-7}	7.84×10^{-12}
Skewness	27.3	98.2	5.33	7.49	7.60	12.6	7.64	4.77	6.92	45.1	7.60	27.3	18.3
Kurtosis	1204	9761	53.8	95.6	104.6	255.6	94.5	38.0	80.8	2633	105	1204	483
Coeff. of Variability	5.15	37.23	1.45	2.32	2.30	3.15	2.66	1.74	2.19	6.67	2.30	5.15	4.46
Range Minimum	7.71×10^{-6}	5.56×10^{-8}	5.81×10^{-6}	1.59×10^{-9}	1.11×10^{-7}	4.87×10^{-7}	1.25×10^{-8}	1.11×10^{-9}	8.17×10^{-8}	7.65×10^{-8}	1.67×10^{-10}	3.47×10^{-9}	1.72×10^{-10}
Range Maximum	8.66×10^{-1}	2.57×10^{-1}	1.43×10^{-0}	3.93×10^{-3}	5.51×10^{-1}	1.98×10^{-1}	2.95×10^{-0}	1.01×10^{-1}	2.10×10^{-1}	3.53×10^{-1}	8.26×10^{-4}	3.90×10^{-2}	1.06×10^{-4}
Range Width	8.66×10^{-1}	2.57×10^{-1}	1.43×10^{-0}	3.93×10^{-3}	5.51×10^{-1}	1.98×10^{-1}	2.95×10^{-0}	1.01×10^{-1}	2.10×10^{-1}	3.53×10^{-1}	8.26×10^{-4}	3.90×10^{-2}	1.06×10^{-4}
Mean Std. Error	1.54×10^{-2}	2.59×10^{-3}	7.59×10^{-4}	1.78×10^{-6}	2.24×10^{-4}	6.11×10^{-5}	1.42×10^{-3}	7.03×10^{-5}	1.04×10^{-4}	5.48×10^{-5}	3.36×10^{-7}	6.91×10^{-6}	2.80×10^{-8}

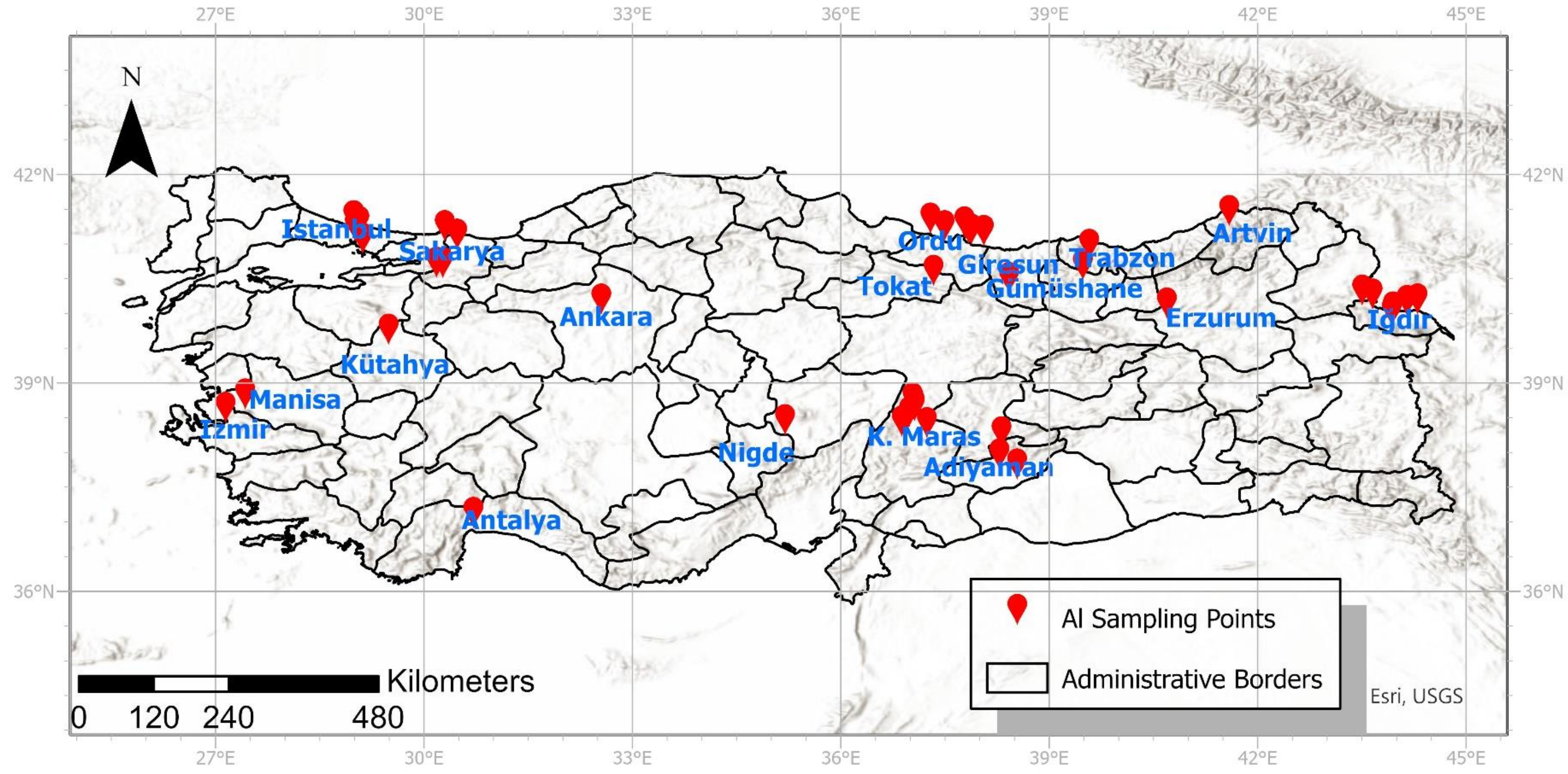

Figure S1.1. Sampling points of Al.

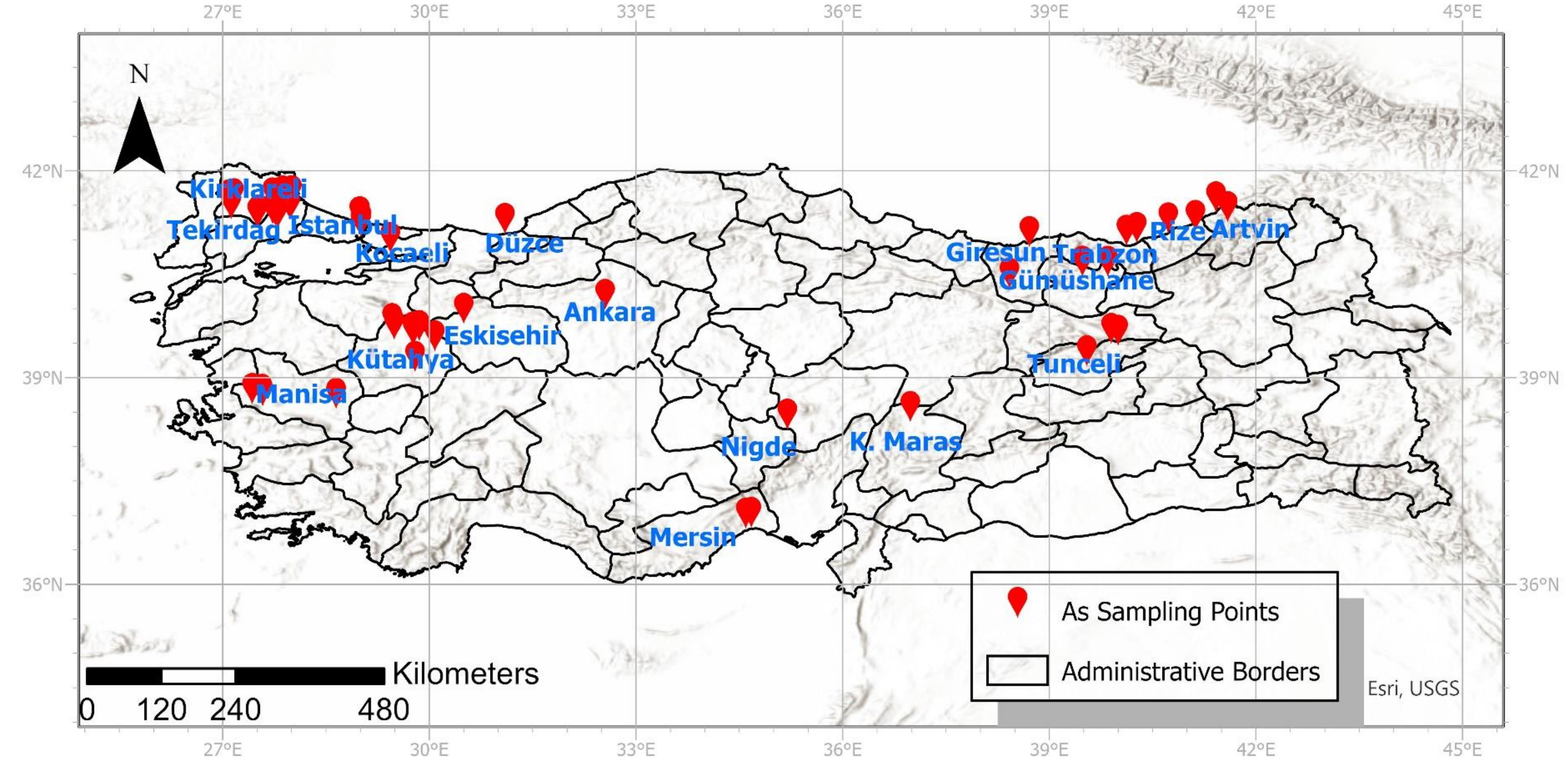

Figure S1.2. Sampling points of As.

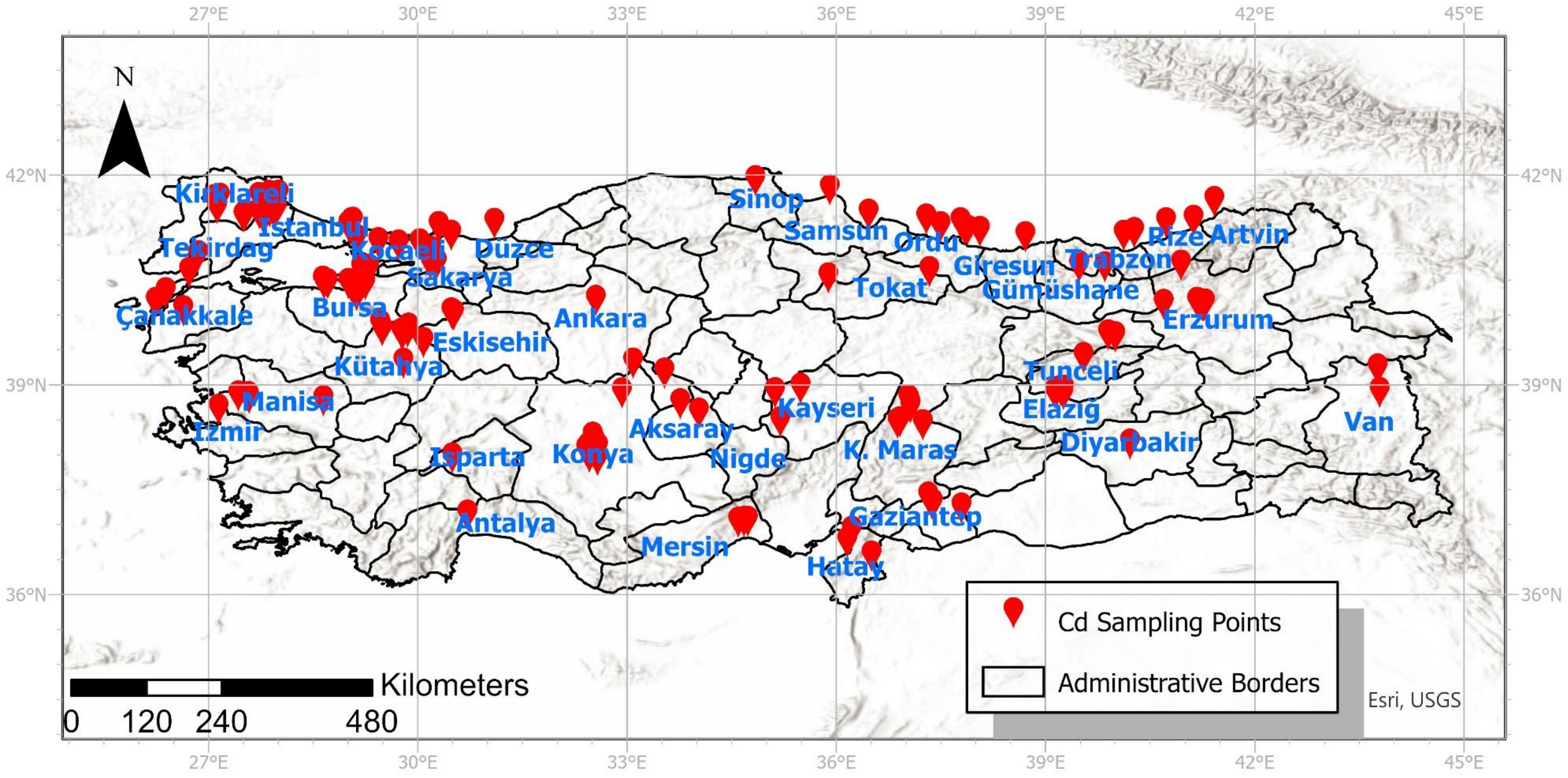

Figure S1.3. Sampling points of Cd.

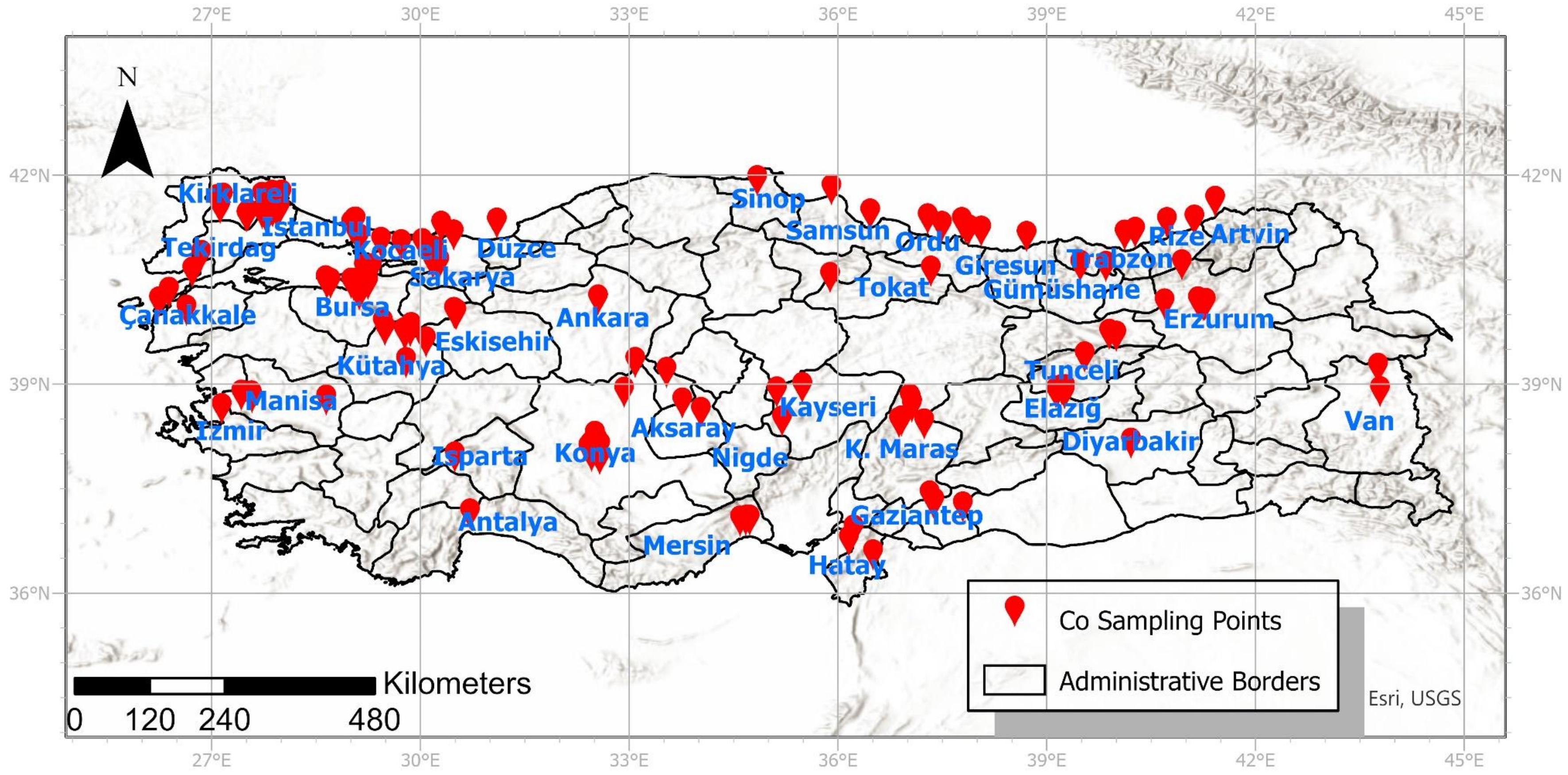

Figure S1.4. Sampling points of Co.

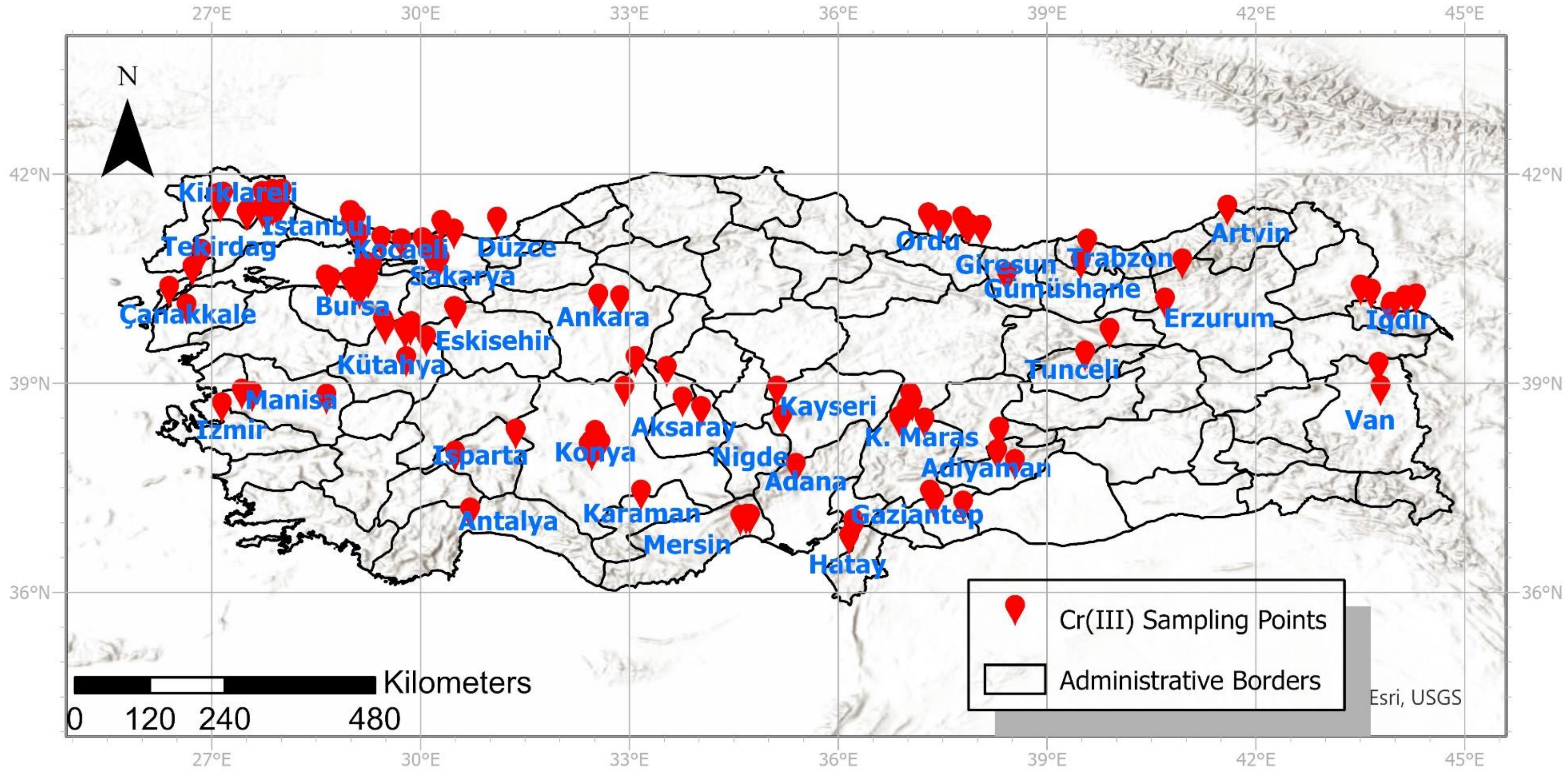

Figure S1.5. Sampling points of Cr (III).

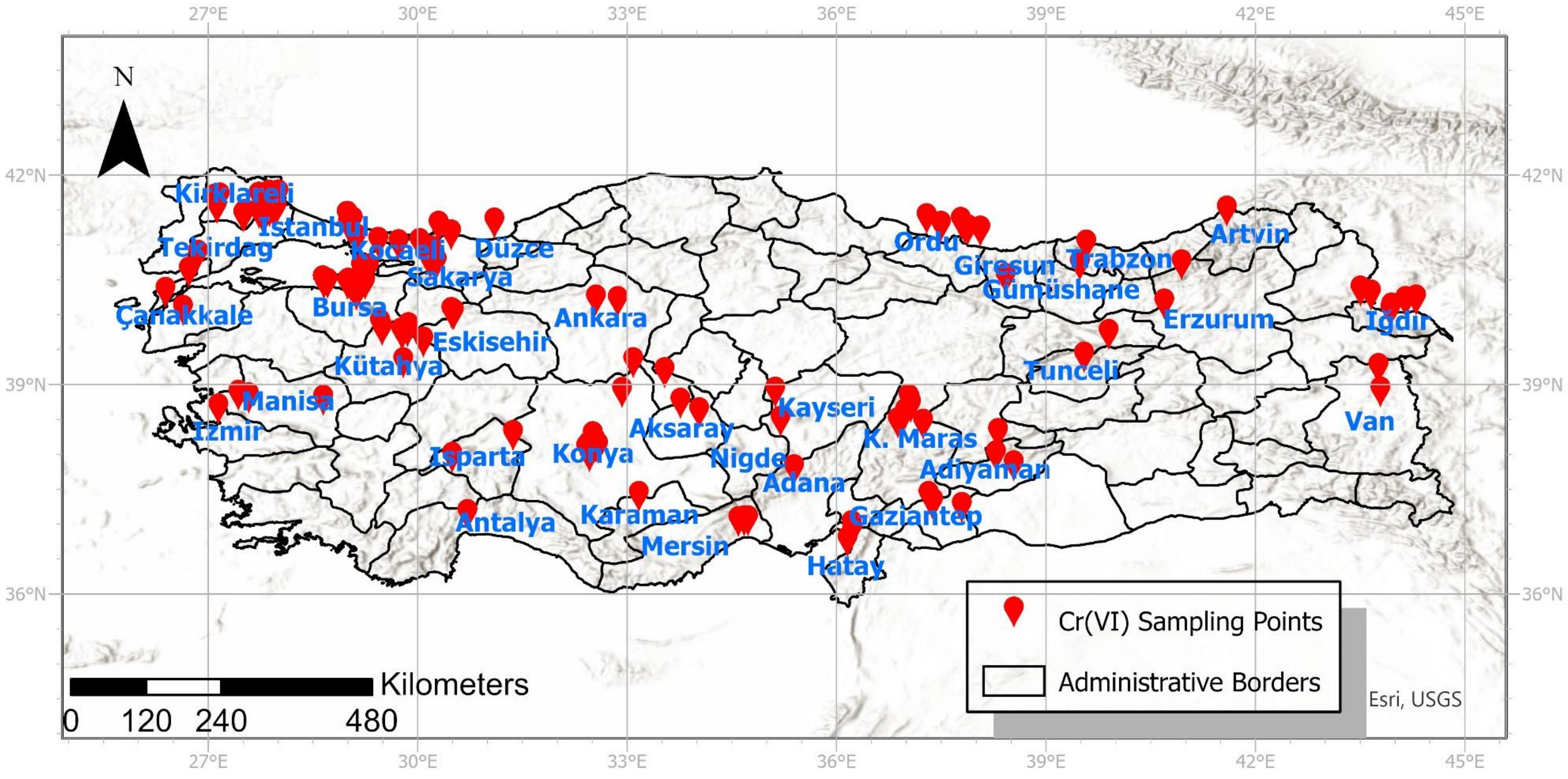

Figure S1.6. Sampling points of Cr (VI).

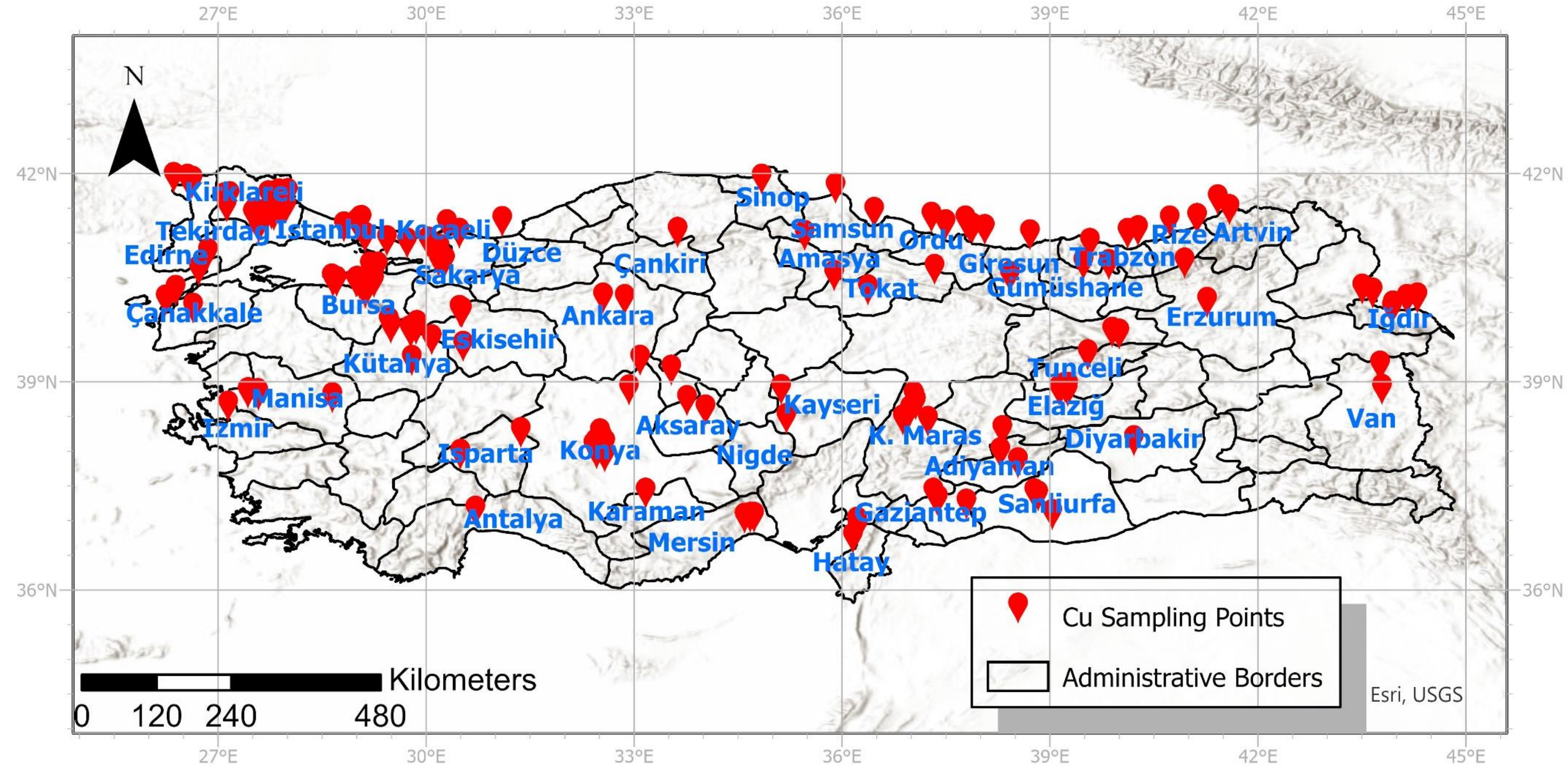

Figure S1.7. Sampling points of Cu.

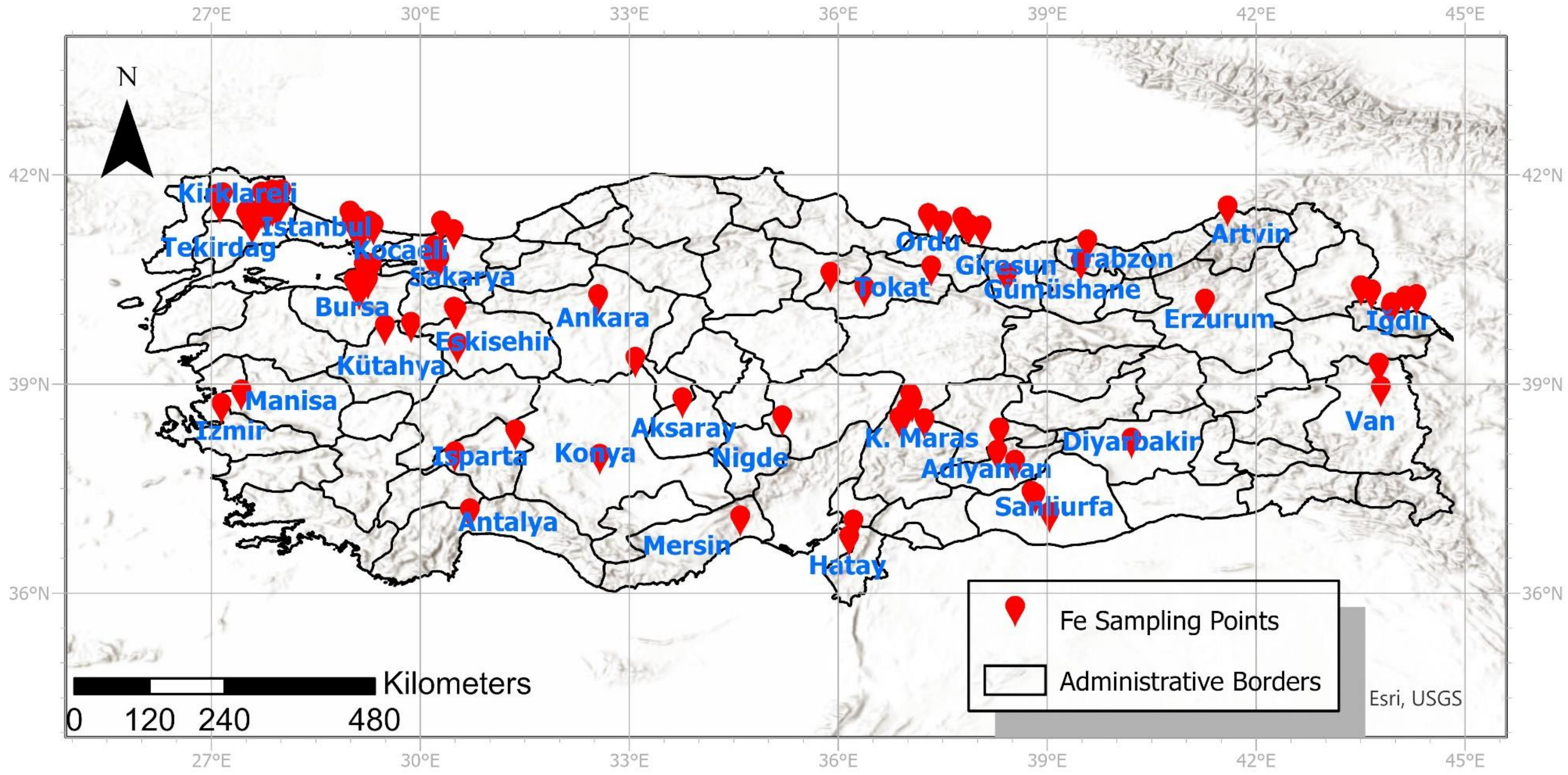

Figure S1.8. Sampling points of Fe.

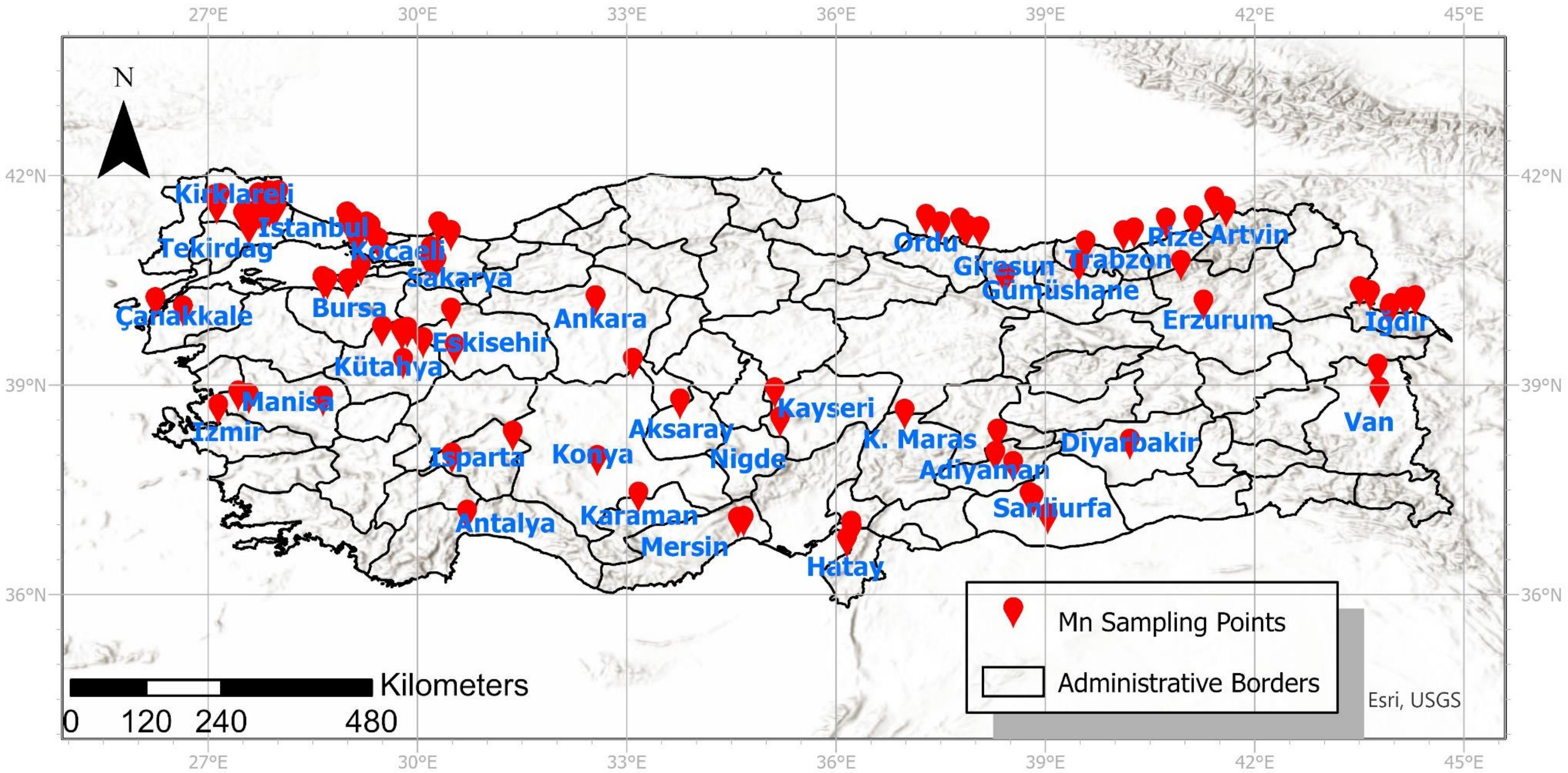

Figure S1.9. Sampling points of Mn.

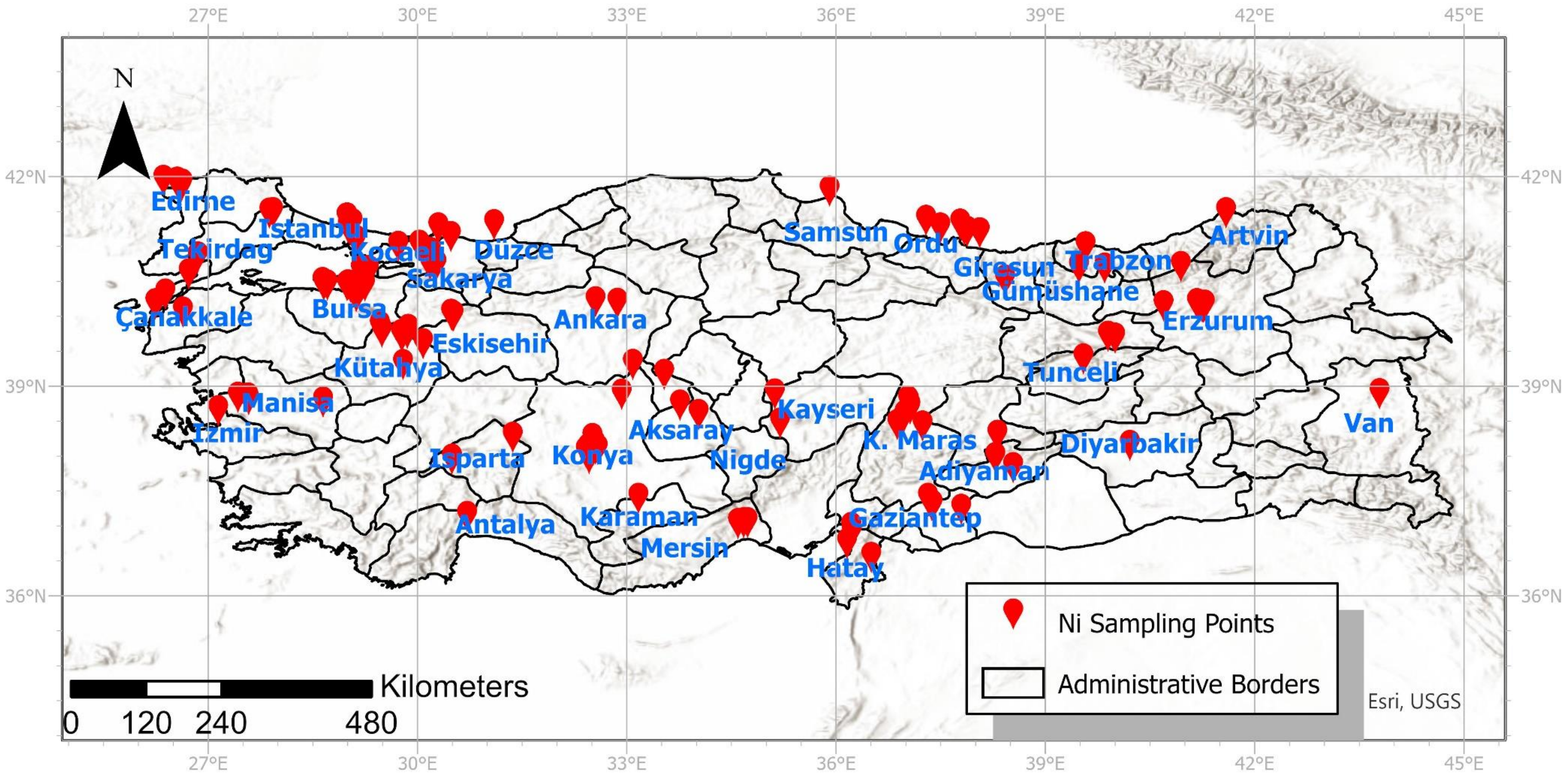

Figure S1.10. Sampling points of Ni.

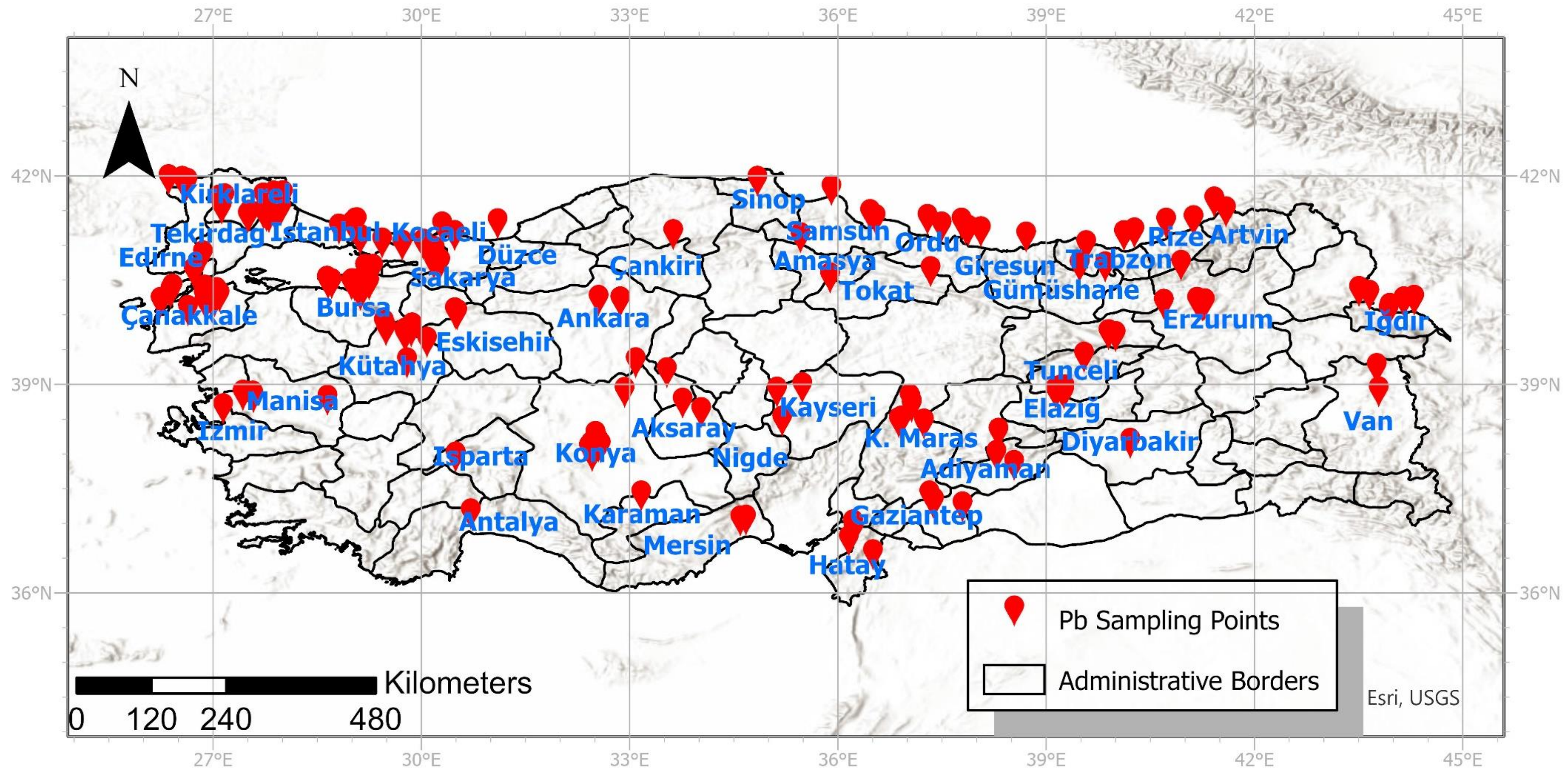

Figure S1.11. Sampling points of Pb.

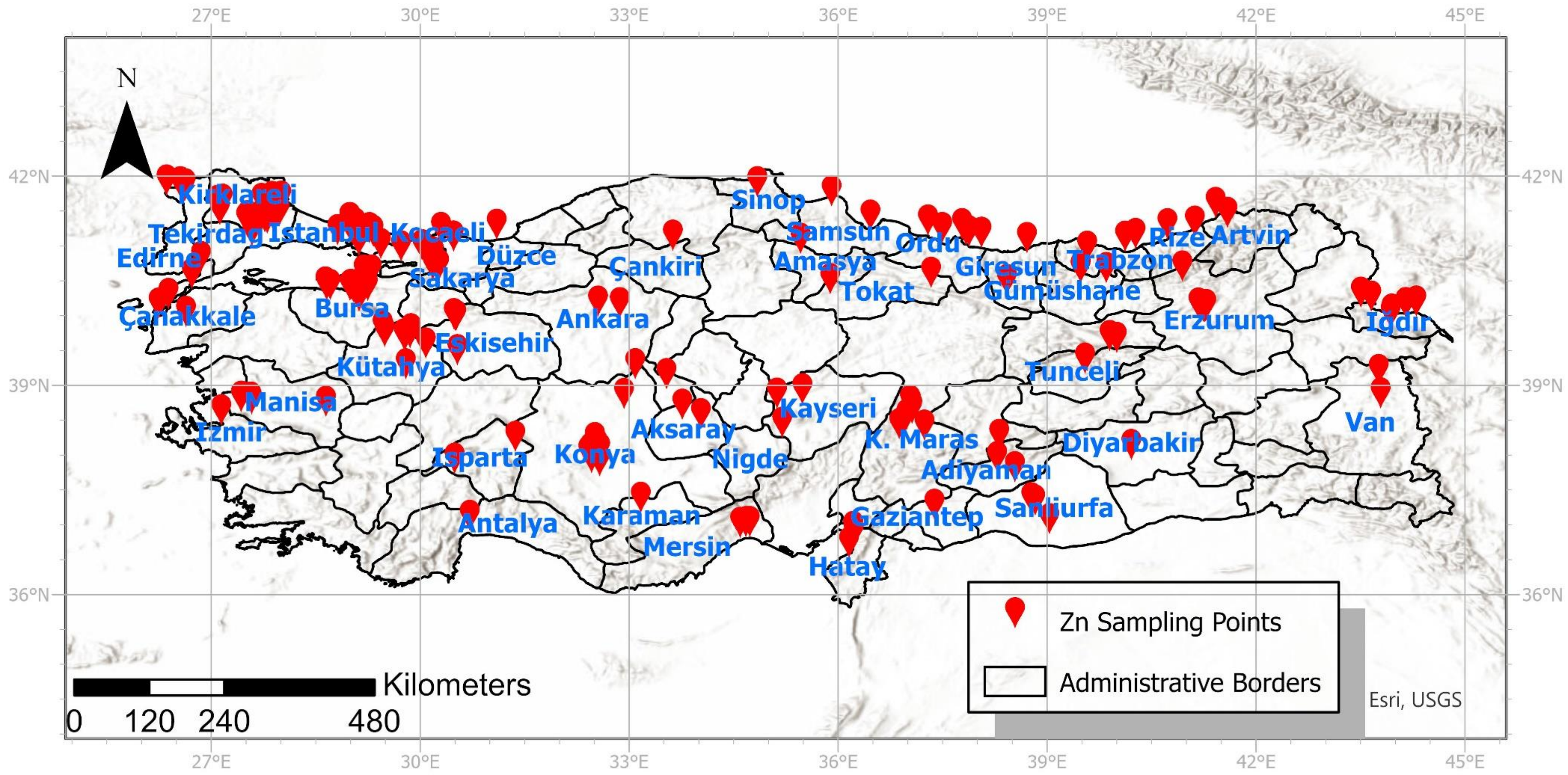

Figure S1.12. Sampling points of Zn.

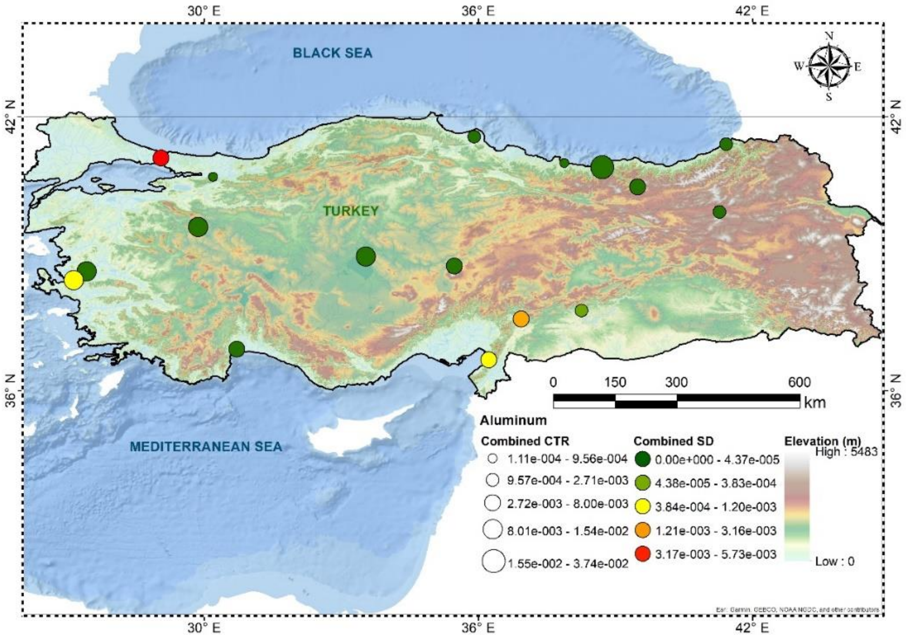

Figure S1.13. Overall CTR levels of Al.

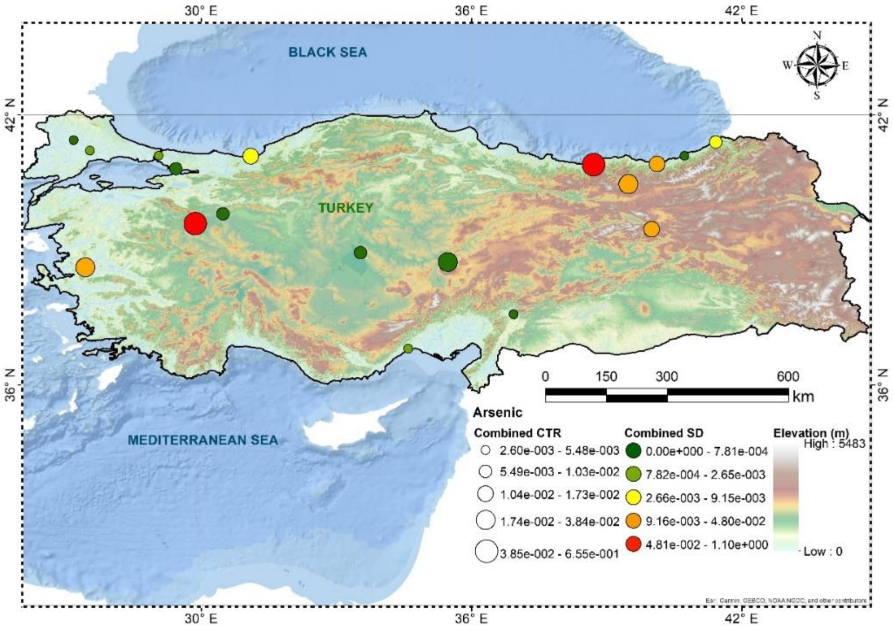

Figure S1.14. Overall CTR levels of As.

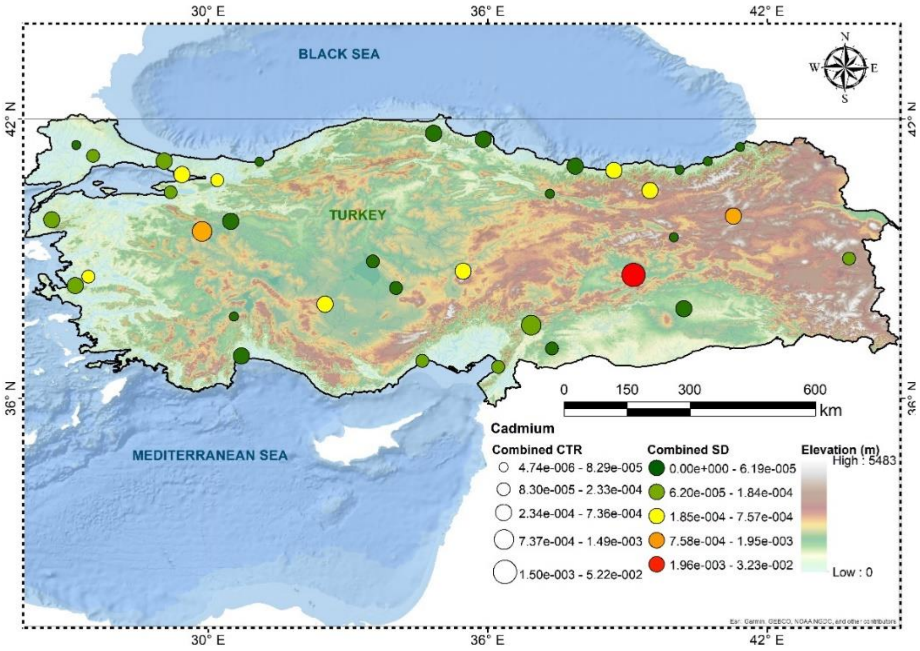

Figure S1.15. Overall CTR levels of Cd.

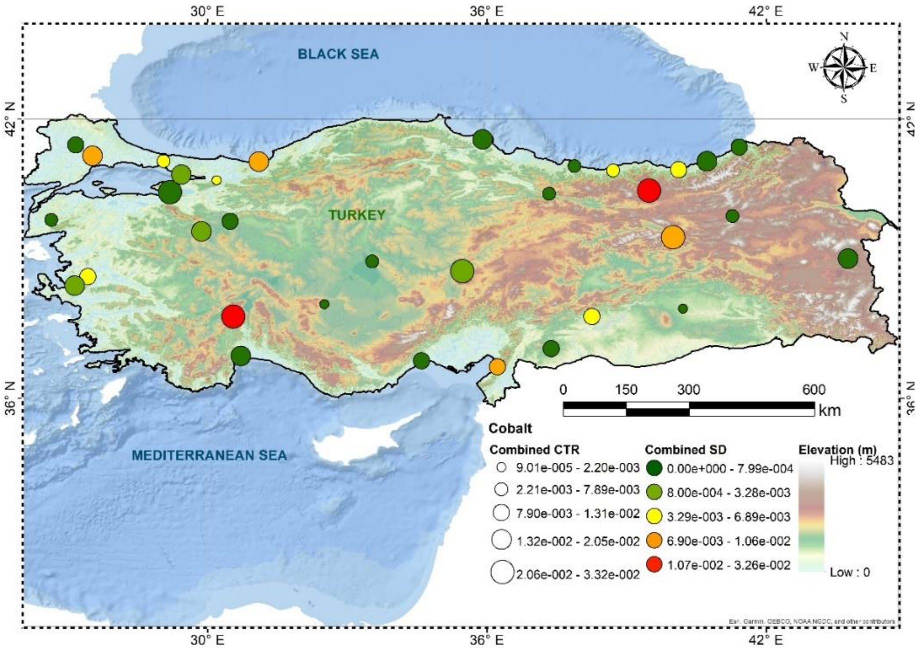

Figure S1.16. Overall CTR levels of Co.

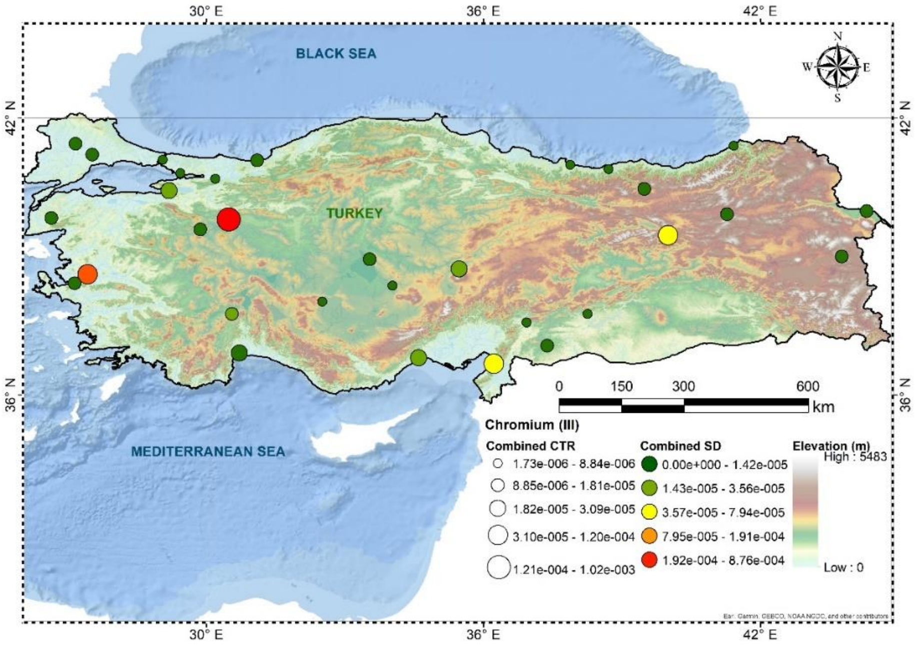

Figure S1.17. Overall CTR levels of Cr(III).

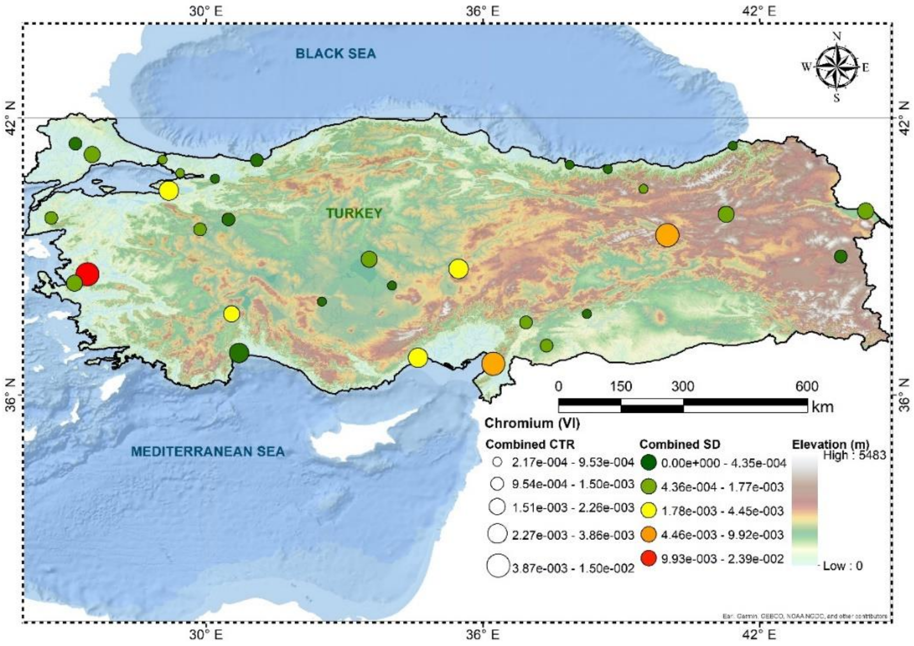

Figure S1.18. Overall CTR levels of Cr(VI).

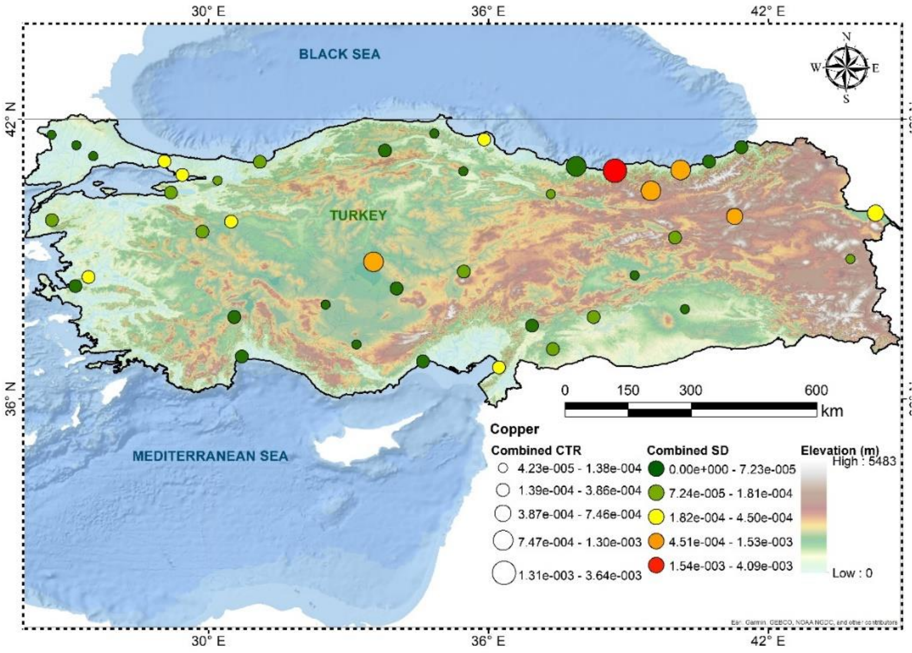

Figure S1.19. Overall CTR levels of Cu.

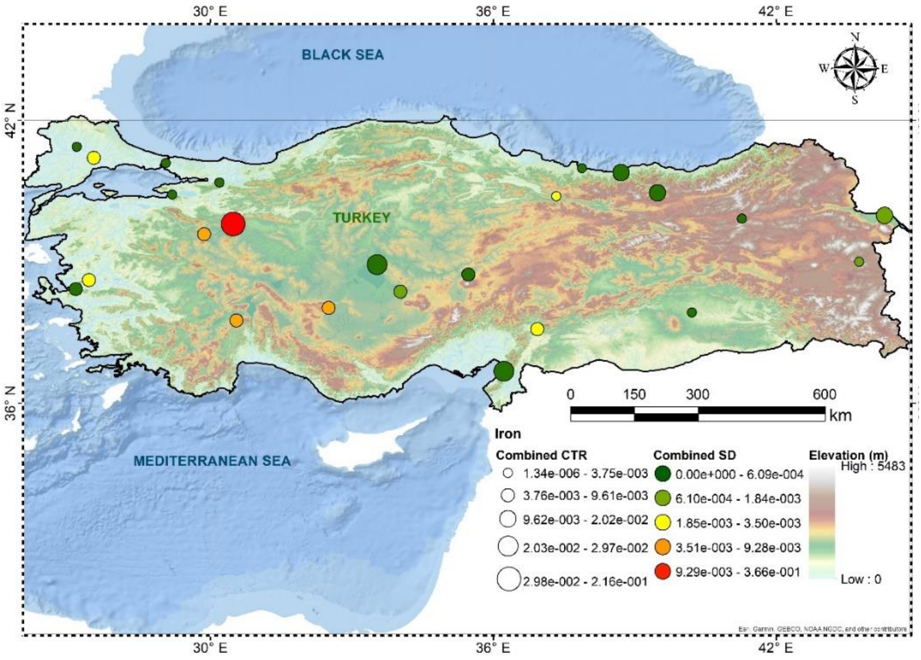

Figure S1.20. Overall CTR levels of Fe.

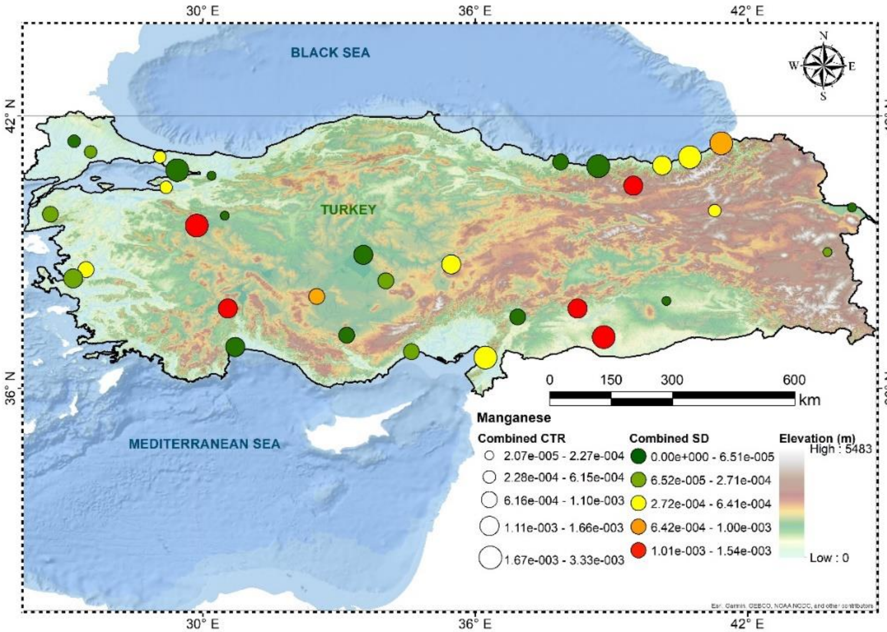

Figure S1.21. Overall CTR levels of Mn.

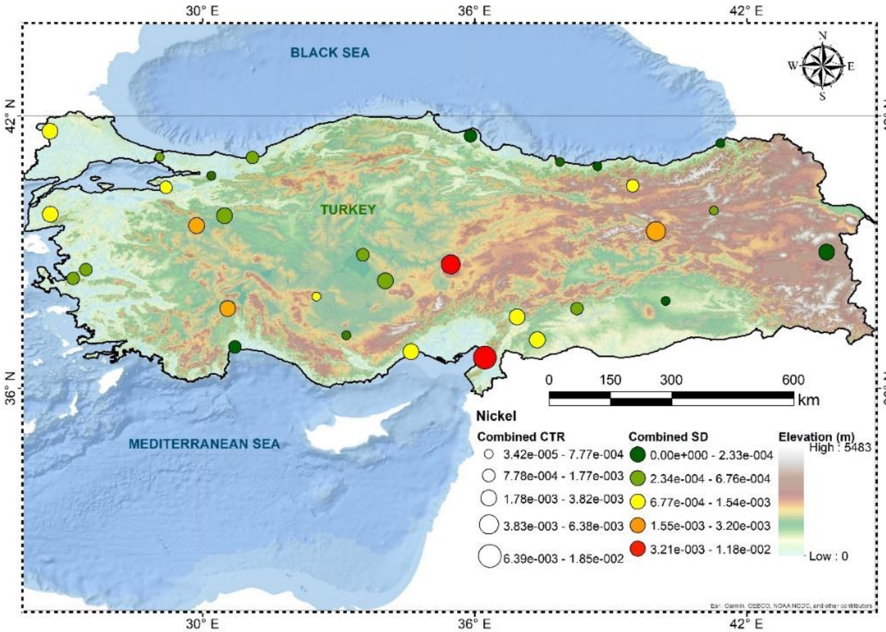

Figure S1.22. Overall CTR levels of Ni.

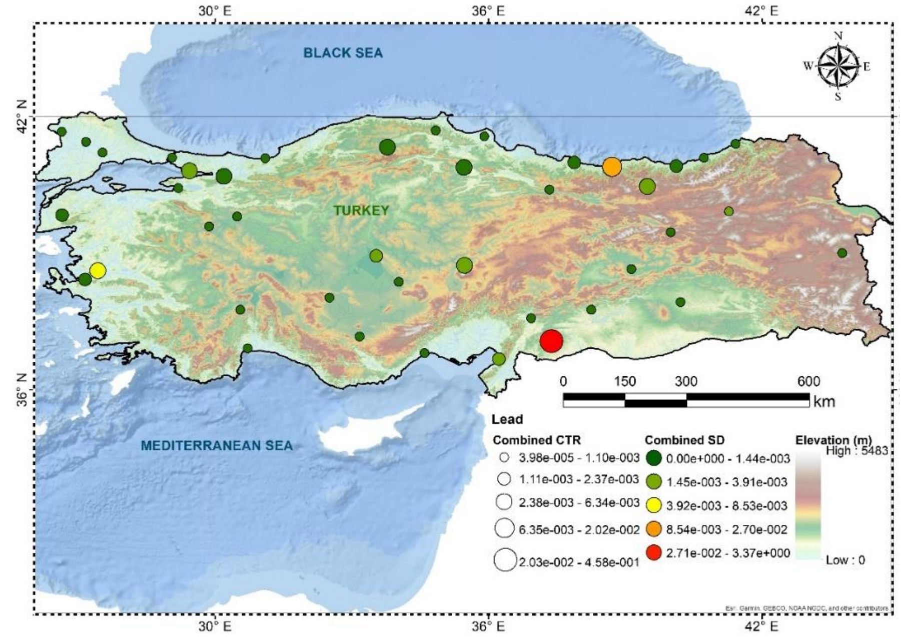

Figure S1.23. Overall CTR levels of Pb.

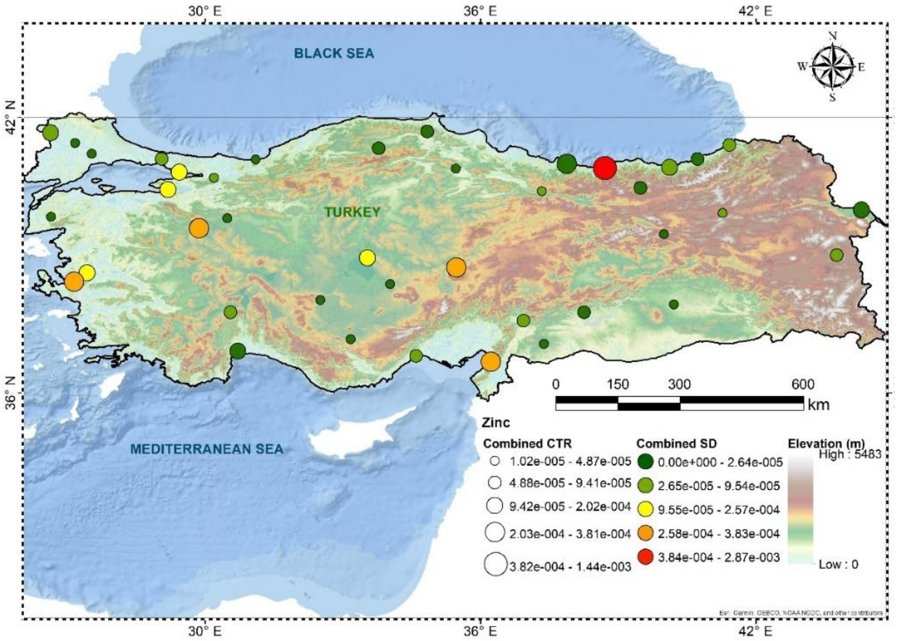

Figure S1.24. Overall CTR levels of Zn.

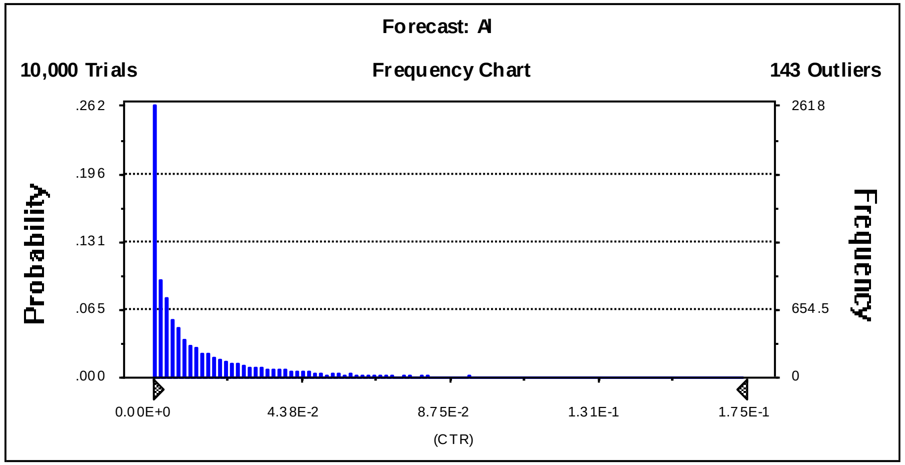

Figure S1.25. Al Associated CTR levels of Turkish Population.

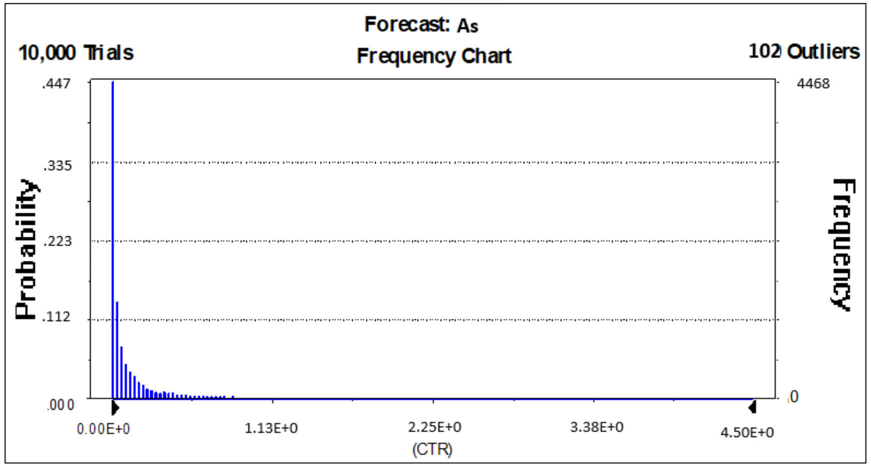

Figure S1.26. Overall CTR levels of As.

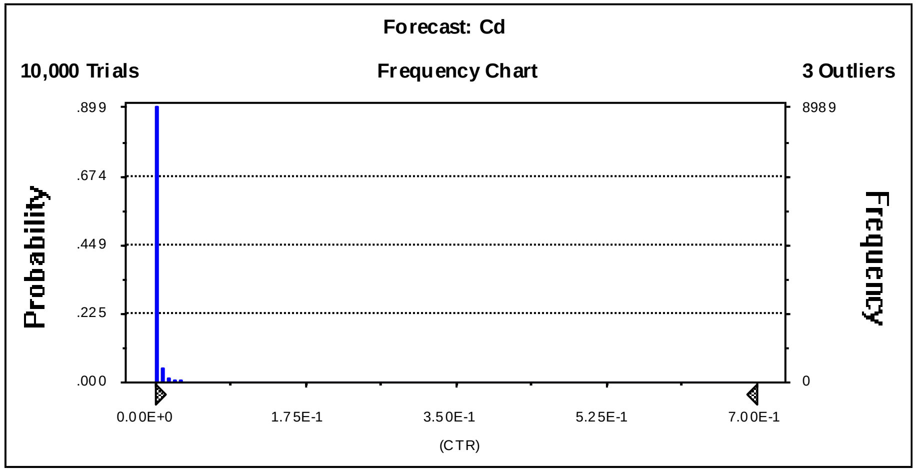

Figure S1.27. Cd Associated CTR levels of Turkish Population.

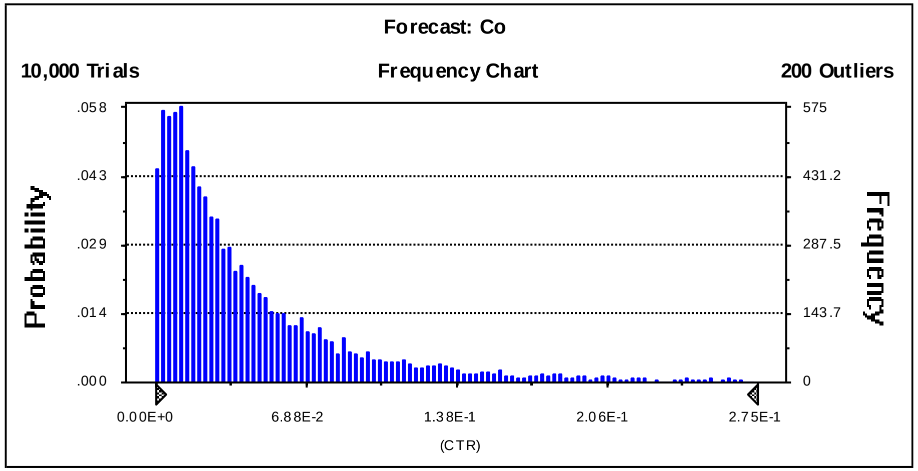

Figure S1.28. Co Associated CTR levels of Turkish Population.

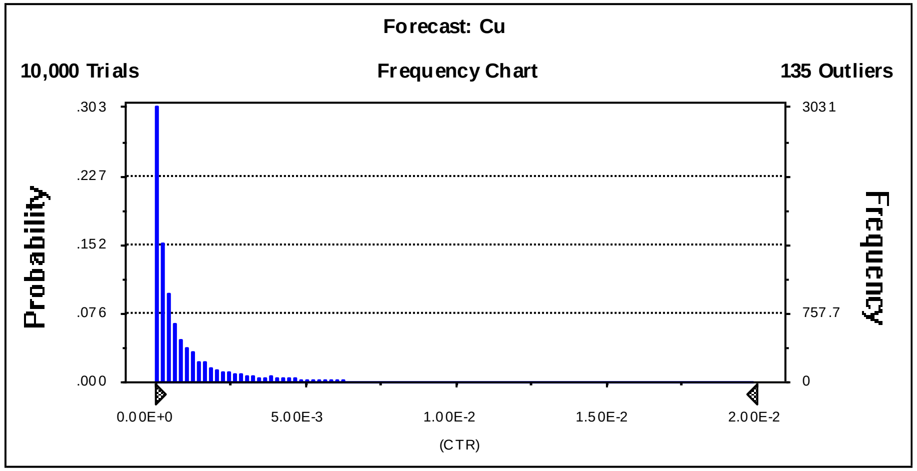

Figure S1.29. Cu Associated CTR levels of Turkish Population.

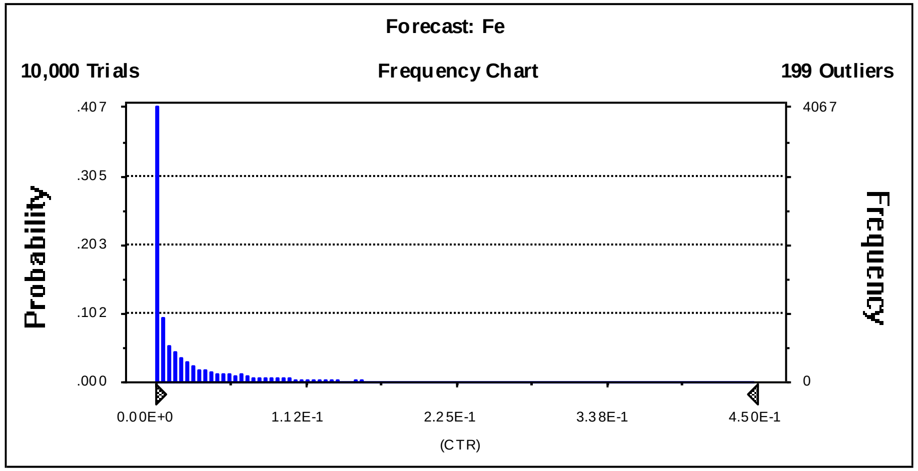

Figure S1.30. Fe Associated CTR levels of Turkish Population.

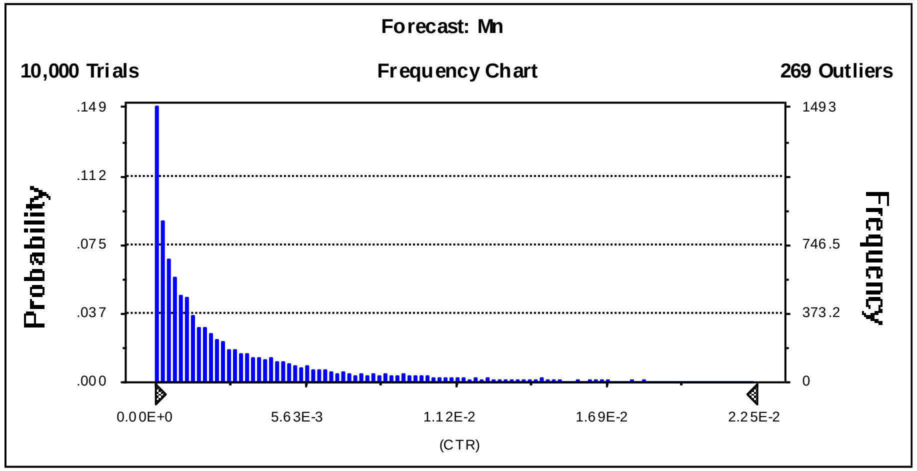

Figure S1.31. Mn Associated CTR levels of Turkish Population.

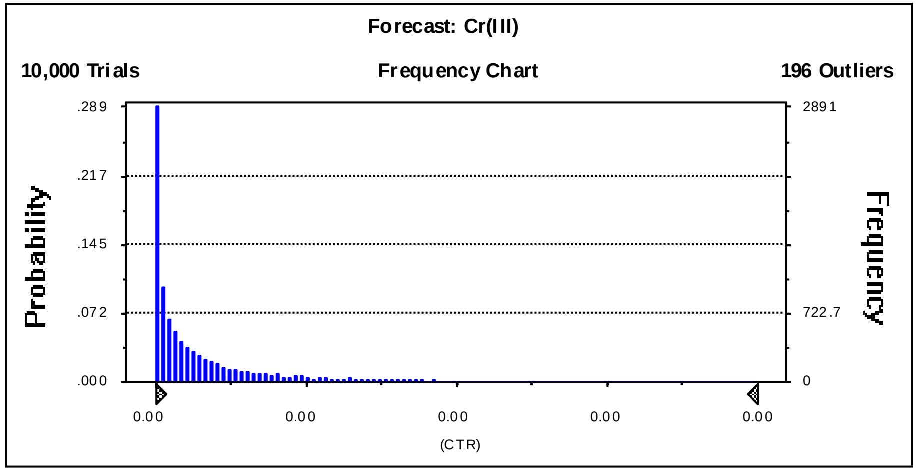

Figure S1.32. Cr(III) Associated CTR levels of Turkish Population.

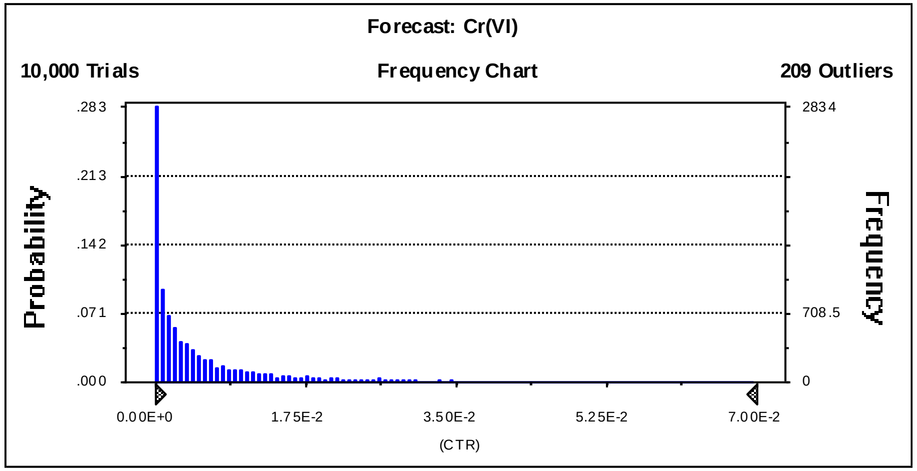

Figure S1.33. Cr(VI) Associated CTR levels of Turkish Population.

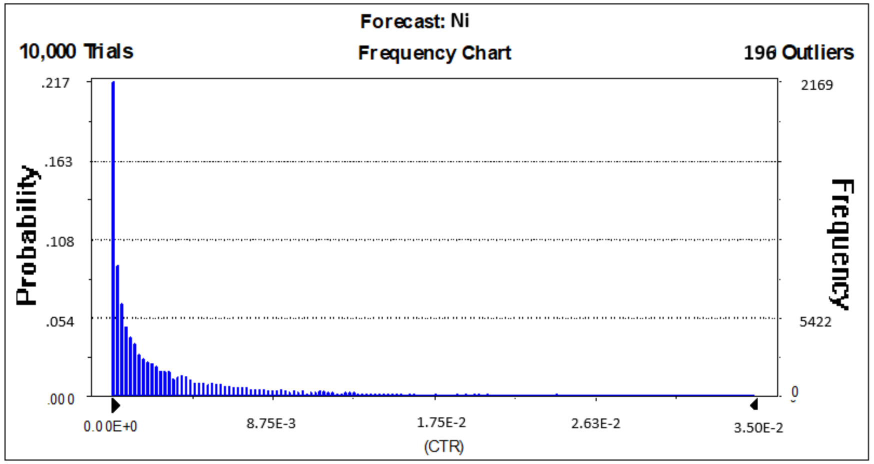

Figure S1.34. Ni Associated CTR levels of Turkish Population.

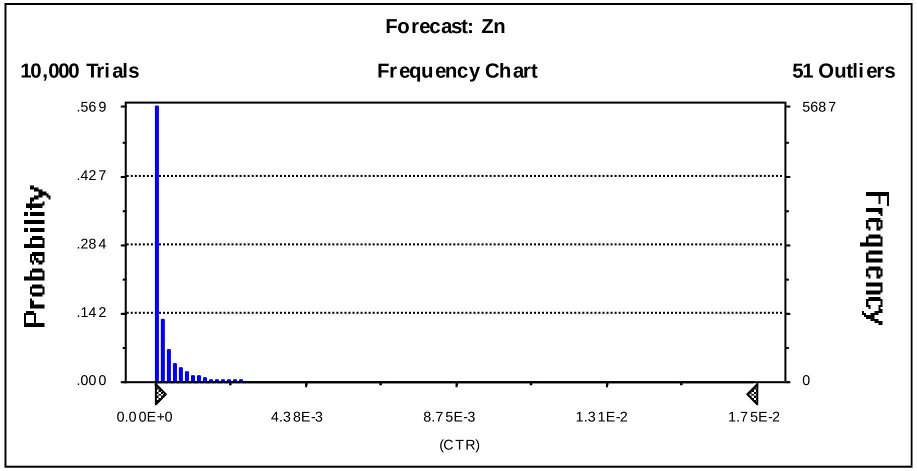

Figure S1.35. Zn Associated CTR levels of Turkish Population.

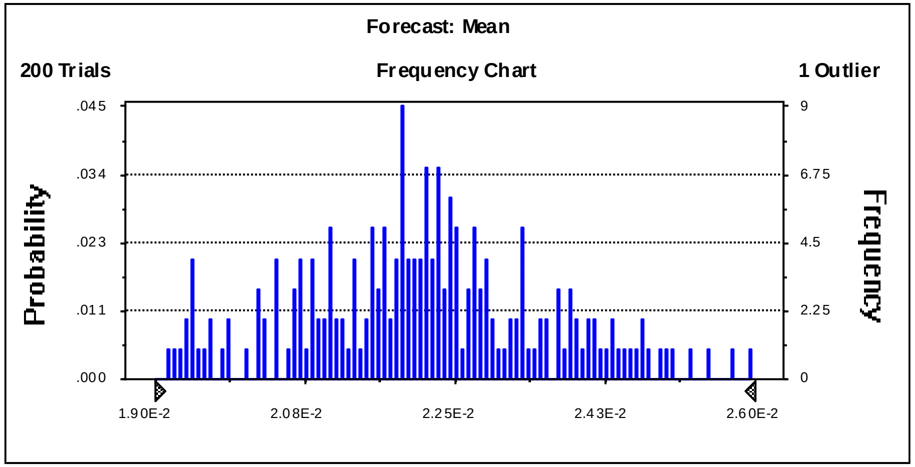

Figure S1.36. Distribution of the bootstrapping mean CTR levels of Al.

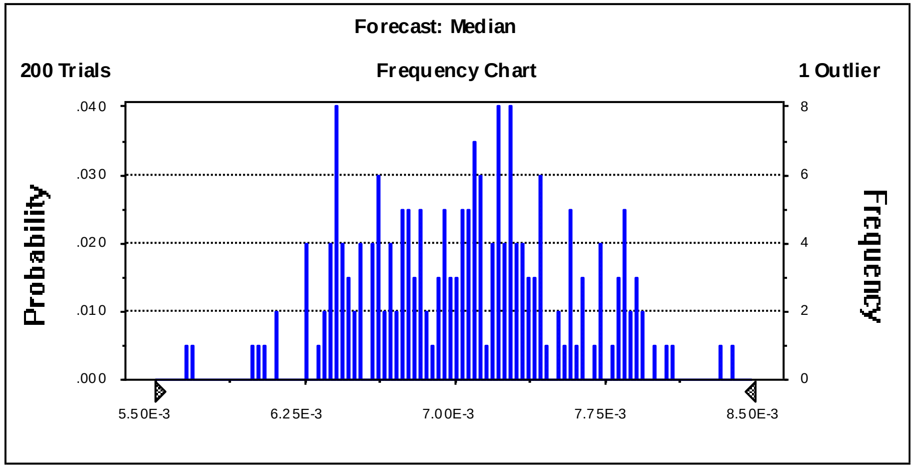

Figure S1.37. Distribution of the bootstrapping median CTR levels of Al.

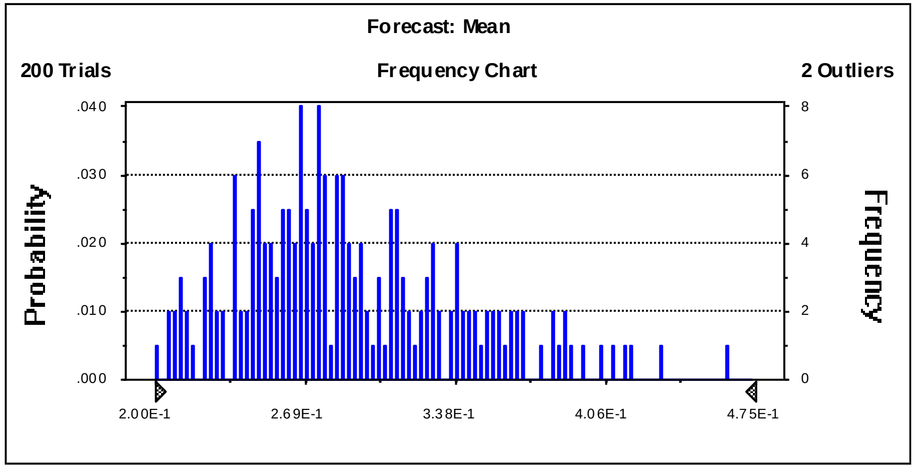

Figure S1.38. Distribution of the bootstrapping mean CTR levels of As.

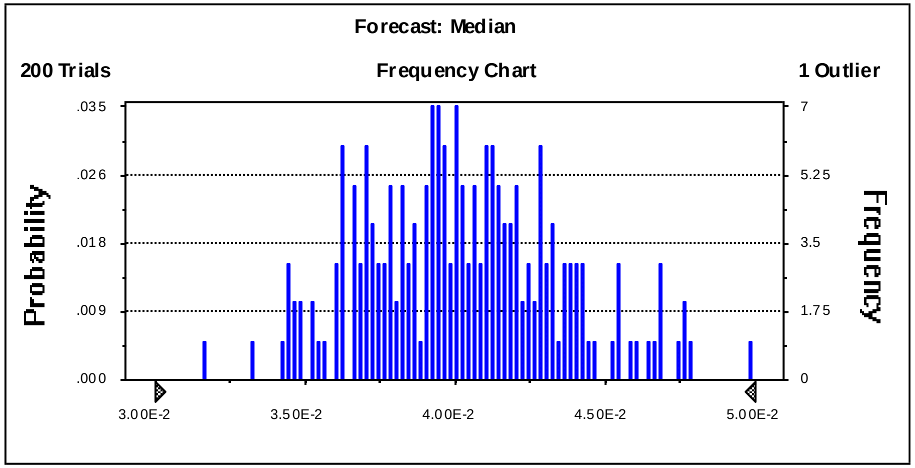

Figure S1.39. Distribution of the bootstrapping median CTR levels of As.

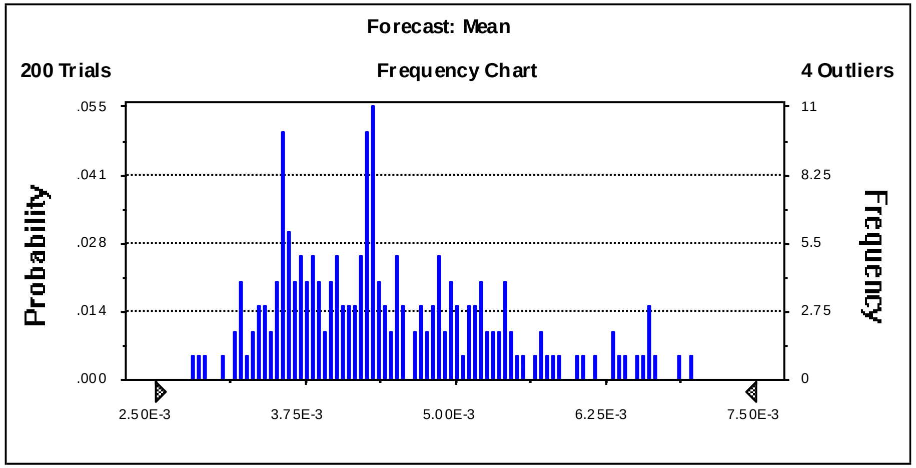

Figure S1.40. Distribution of the bootstrapping mean CTR levels of Cd.

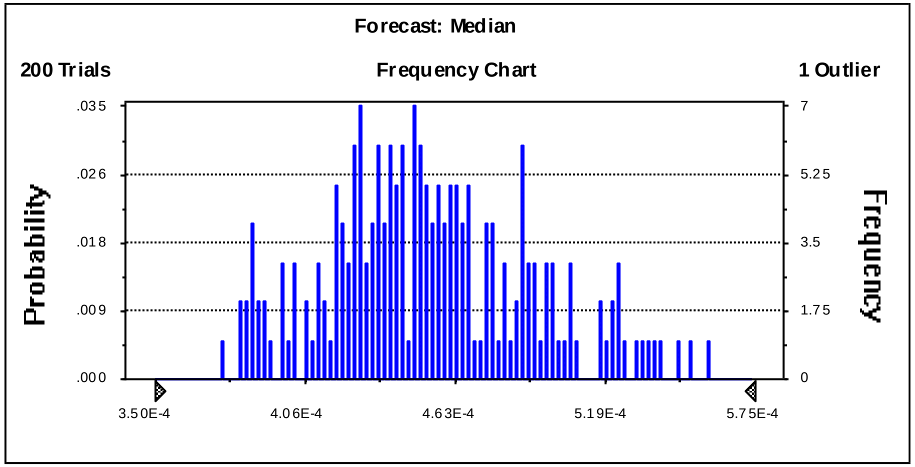

Figure S1.41. Distribution of the bootstrapping median CTR levels of Cd.

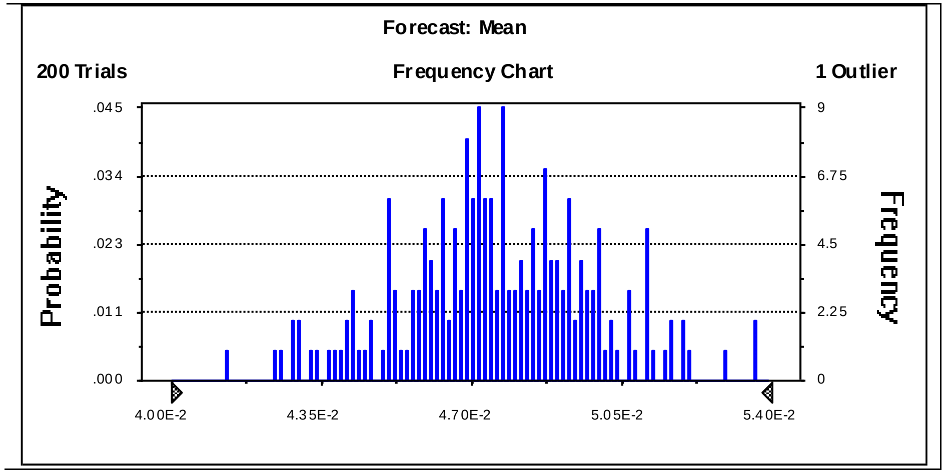

Figure S1.42. Distribution of the bootstrapping mean CTR levels of Co.

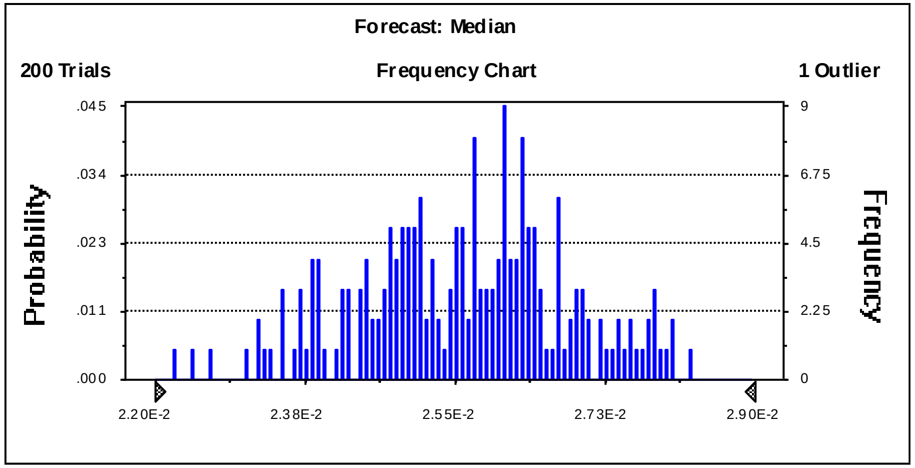

Figure S1.43. Distribution of the bootstrapping median CTR levels of Co.

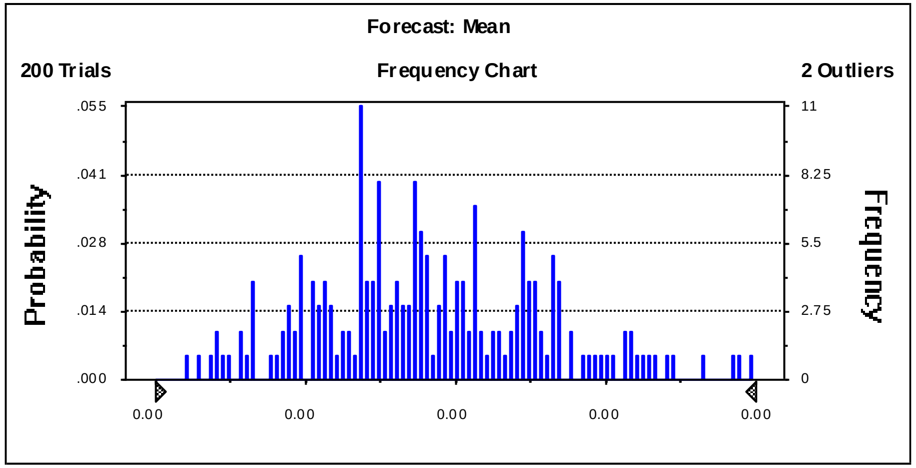

Figure S1.44. Distribution of the bootstrapping mean CTR levels of Cr(III).

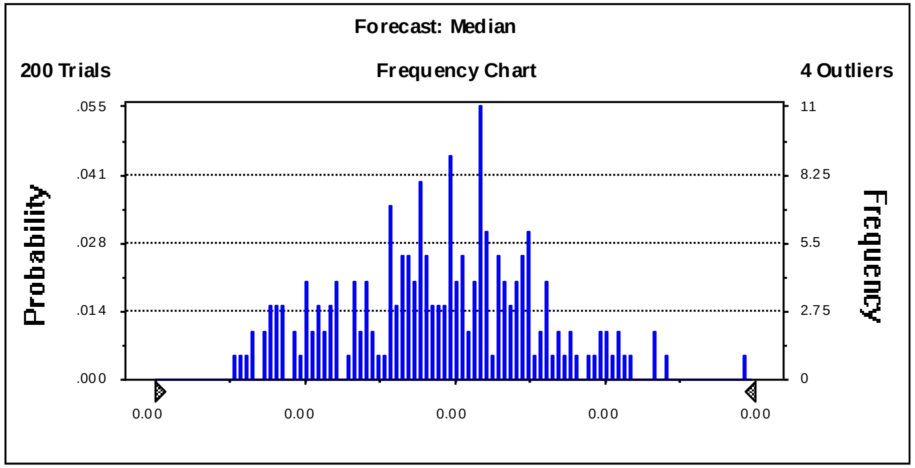

Figure S1.45. Distribution of the bootstrapping median CTRs of Cr(III).

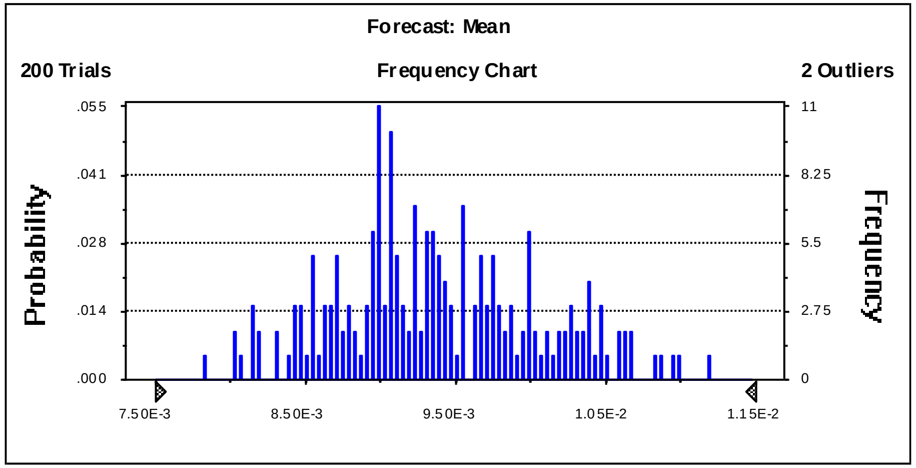

Figure S1.46. Distribution of the bootstrapping mean CTRs of Cr(VI).

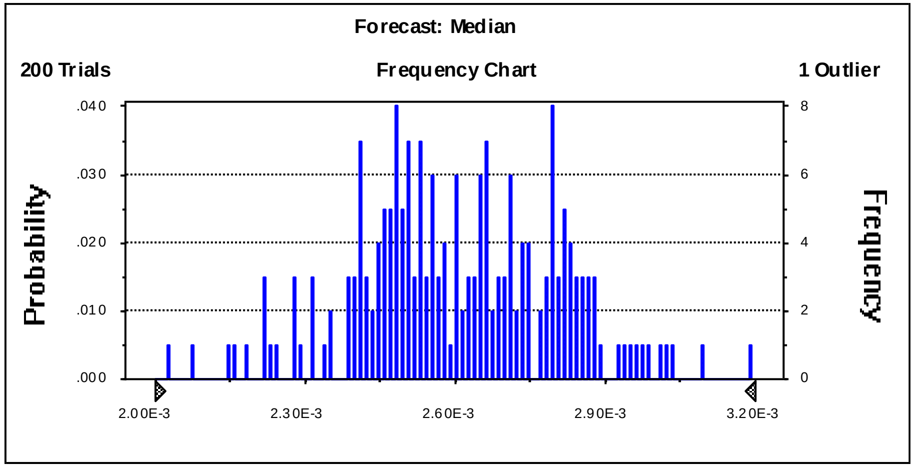

Figure S1.47. Distribution of the bootstrapping median CTRs of Cr(VI).

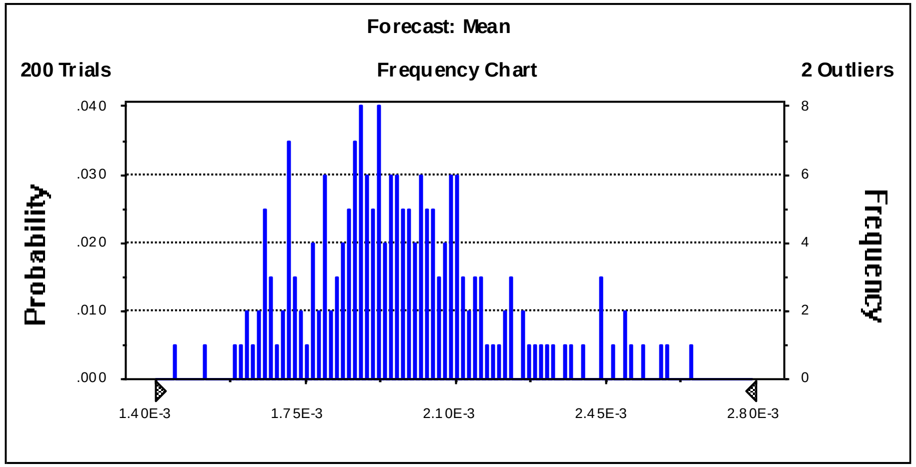

Figure S1.48. Distribution of the bootstrapping mean CTRs of Cu.

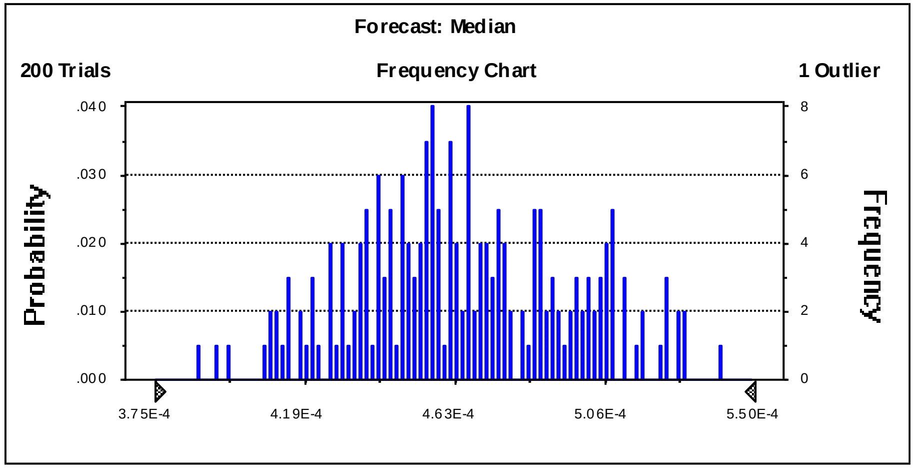

Figure S1.49. Distribution of the bootstrapping mean CTRs of Cu.

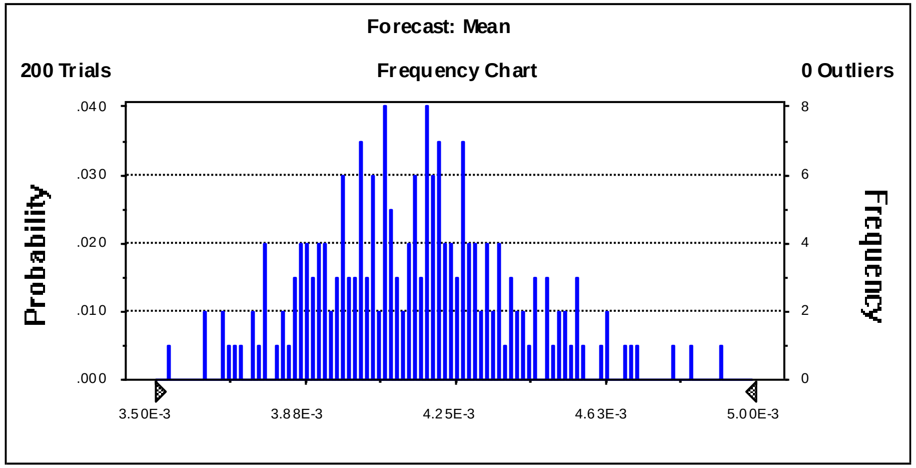

Figure S1.50. Distribution of the bootstrapping mean CTRs of Mn.

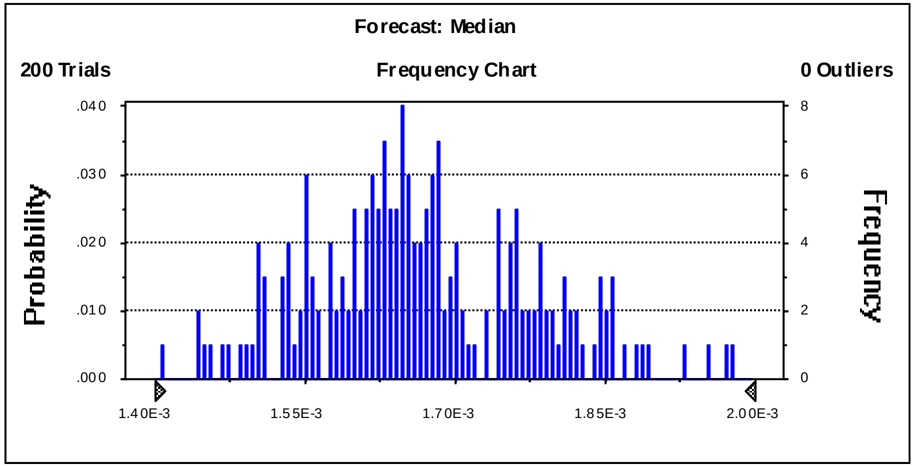

Figure S1.51. Distribution of the bootstrapping median CTRs of Mn.

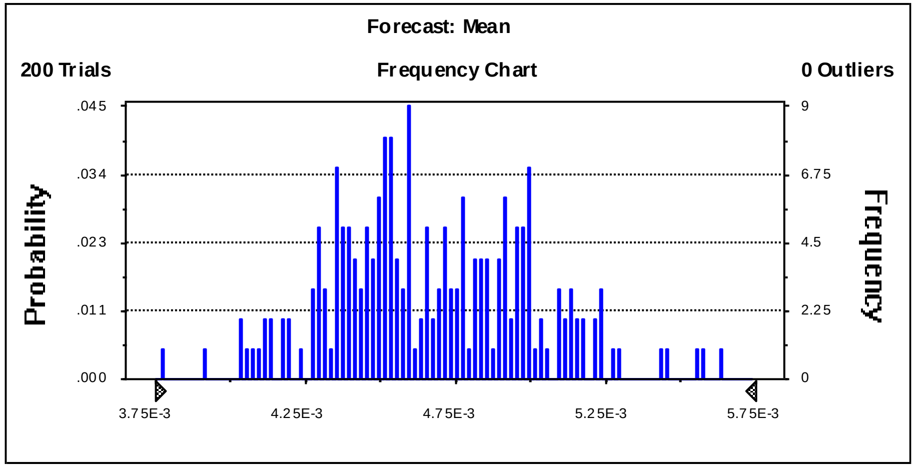

Figure S1.52. Distribution of the bootstrapping mean CTRs of Ni.

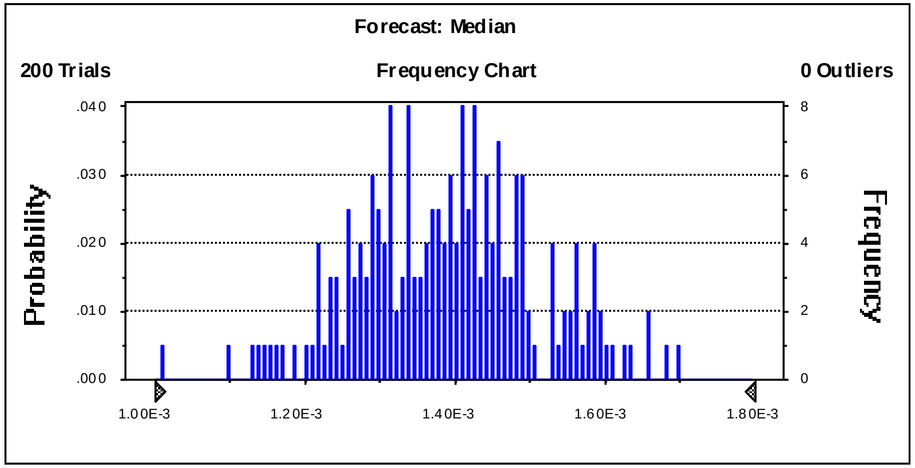

Figure S1.53. Distribution of the bootstrapping median CTRs of Ni.

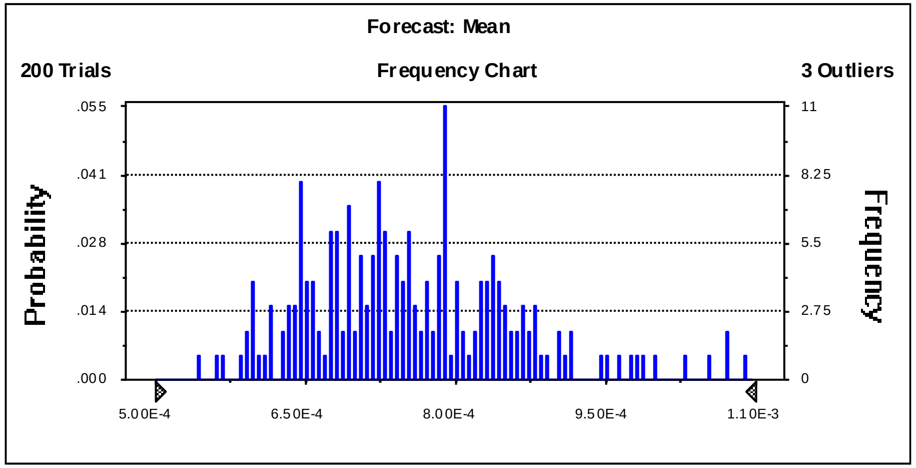

Figure S1.54. Distribution of the bootstrapping mean CTRs of Zn.

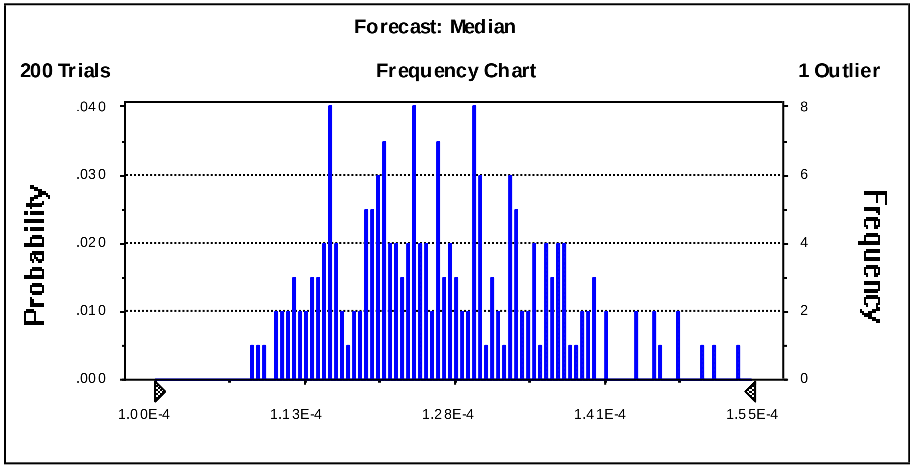

Figure S1.55. Distribution of the bootstrapping median CTRs of Zn.

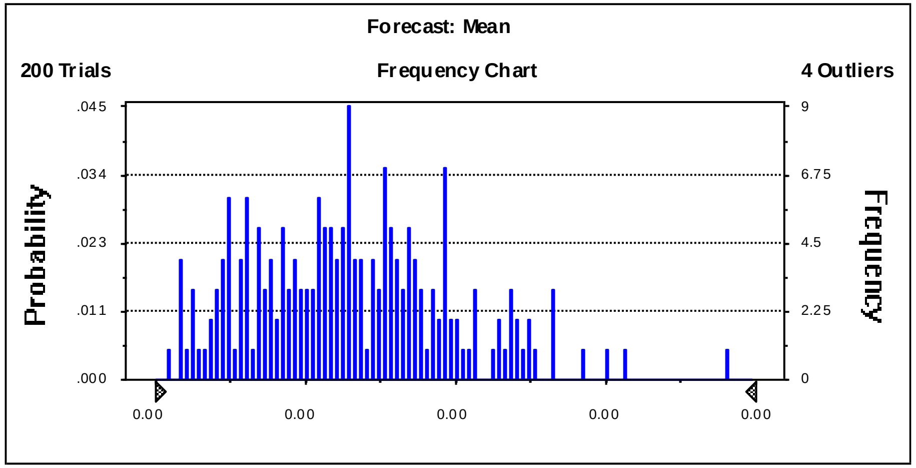

Figure S1.56. Distribution of the bootstrapping mean CRs of As.

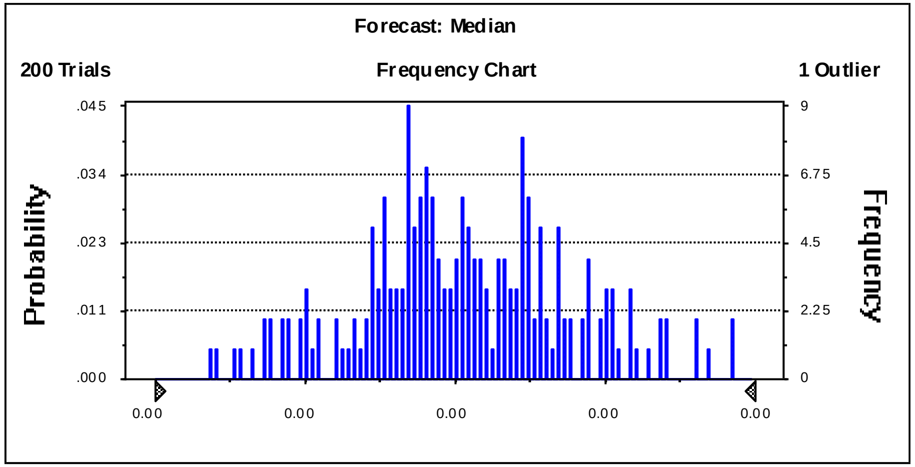

Figure S1.57. Distribution of the bootstrapping median CRs of As.

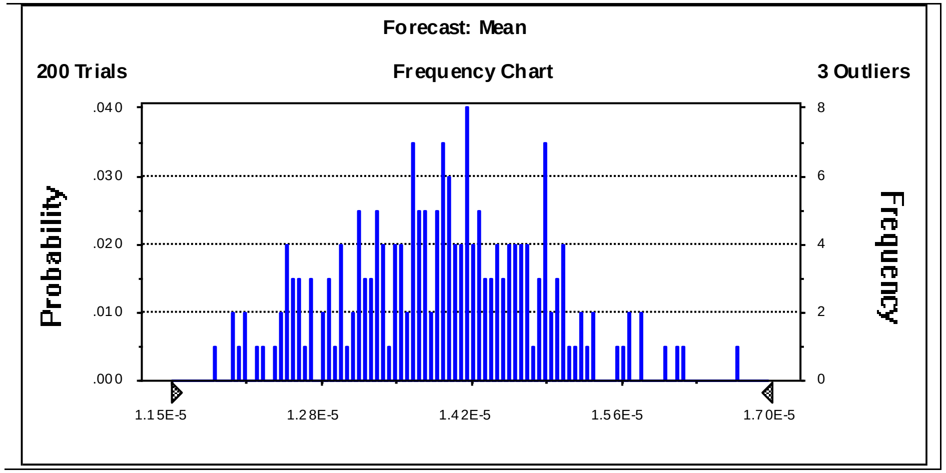

Figure S1.58. Distribution of the bootstrapping mean CRs of Cr(VI).

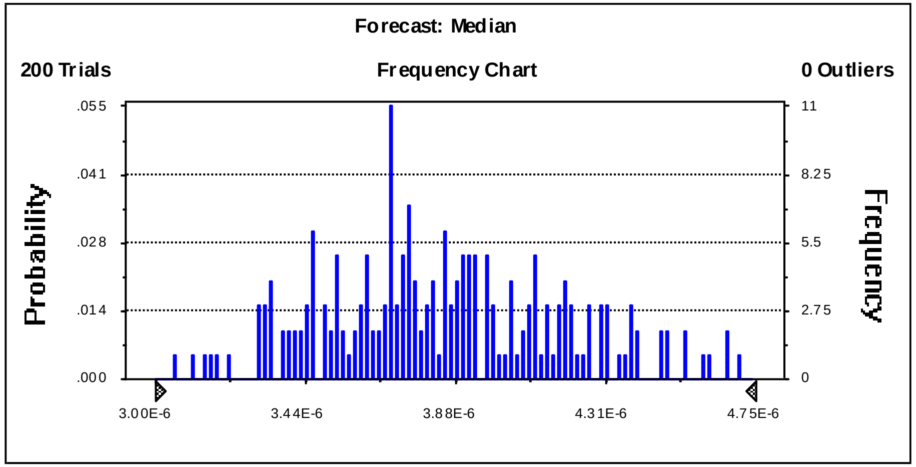

Figure S1.59. Distribution of the bootstrapping median CR levels of Cr(VI).

References

(1) Polat, F.; Dogan, H. M.; Buhan, E.; Kilic, O. M.; Yilmaz, D. S.; Buhan, S. D. ACCUMULATION AND BEHAVIOR OF SOME HEAVY METALS IN THE MAIN COMPONENTS OF ZINAV LAKE BASIN ECOSYSTEM. *Fresenius Environ. Bull.* **2015**, *24*, 180–187.

(2) Gurel, S.; Sahan, Y.; Basar, H. Antioxidative Properties of Olive Fruits (Olea Europaea L.) from 'Gemlik'Variety and Relationship with Soil Properties and Mineral Composition. *Oxid. Commun.* **2014**, *37* (4), 985–1004.

(3) Susam, T. Application of Geostatistical Techniques in Mapping Topsoil and Subsoil Chemical Composition. *J. Eng. Res.* **2015**, *3* (2), 1–16.

(4) Hanedar, A. Assessment of Airborne Heavy Metal Pollution in Soil and Lichen in the Meric-Ergene Basin, Turkey. *Environ. Technol.* **2015**, *36* (20), 2588–2602.

(5) Çiçek, A.; Koparal, A. S. Assessment of Environmental Effects of the Coal Used in the Seyitömer Thermal Power Plant (Turkey) on White Willow. *Commun. Soil Sci. Plant Anal.* **2006**, *37* (13–14), 1795–1804.

(6) Ekmekyapar, F.; Sabudak, T.; Seren, G. Assessment of Heavy Metal Contamination in Soil and Wheat (Triticum Aestivum L.) Plant around the Çorlu–Çerkezkoy Highway in Thrace Region. *Glob. NEST J.* **2012**, *14* (4), 496–504.

(7) Vural, A. Assessment of Metal Pollution Associated with an Alteration Area: Old Gümüşhane, NE Black Sea. *Environ. Sci. Pollut. Res.* **2015**, *22* (5), 3219–3228.

(8) Çimen, O.; Öztüfekçi Önal, A.; Akyol, E. A. Assessment of Pollution Potential of the Hasangazi Chromite Pit (Tunceli, Turkey): Implications for the Natural Environment. *Environ. earth Sci.* **2018**, *77* (5), 1–17.

(9) Ağca, N.; Özdel, E. Assessment of Spatial Distribution and Possible Sources of Heavy Metals in the Soils of Sariseki-Dörtyol District in Hatay Province (Turkey). *Environ. Earth Sci.* **2014**, *71* (3), 1033–1047.

(10) Malkoc, S.; Yazıcı, B.; Savas Koparal, A. Assessment of the Levels of Heavy Metal Pollution in Roadside Soils of Eskisehir, Turkey. *Environ. Toxicol. Chem.* **2010**, *29* (12), 2720–2725.

(11) Arslan, Ş.; Çelik, M. Assessment of the Pollutants in Soils and Surface Waters around Gümüşköy Silver Mine (Kütahya, Turkey). *Bull. Environ. Contam. Toxicol.* **2015**, *95* (4), 499–506.

(12) Tarcan, G.; Akıncı, G.; Danışman, M. A. Assessment of the Pollution from Tannery Effluents upon Waters and Soils in and around Kula Vicinity, Turkey. *Water, Air, Soil Pollut.* **2010**, *213* (1), 199–210.

(13) Avci, H.; Deveci, T. Assessment of Trace Element Concentrations in Soil and Plants from Cropland Irrigated with Wastewater. *Ecotoxicol. Environ. Saf.* **2013**, *98*, 283–291.

(14) Özkutlu, F.; Turan, M.; Korkmaz, K.; Huang, Y. M. Assessment of Heavy Metal Accumulation in the Soils and Hazelnut Plant (Corylus Avellena L.) from Black Sea Coastal Region of Turkey. *Asian J. Chem.* **2009**, *21* (6), 4371–4388.

(15) Akbulut, S.; Çevik, U. Accumulation of Metals in Roadside Soil, Dust and Pine

Needles in Different Characteristic Traffic Areas. **2014**.

(16) Celik, S.; Yucel, E.; Celik, S.; Gucel, S.; Ozturk, M. Carolina Poplar (Populus x Canadensis Moench) as a Biomonitor of Trace Elements in Black Sea Region of Turkey. *J. Environ. Biol.* **2010**, *31* (1), 225.

(17) Banar, M.; Özkan, A.; Vardar, Ç. Characterization of an Urban Landfill Soil by Using Physicochemical Analysis and Solid Phase Microextraction (SPME)—GC/MS. *Environ. Monit. Assess.* **2007**, *127* (1), 337–351.

(18) Sungur, A.; Soylak, M.; Yilmaz, E.; Yilmaz, S.; Ozcan, H. Characterization of Heavy Metal Fractions in Agricultural Soils by Sequential Extraction Procedure: The Relationship between Soil Properties and Heavy Metal Fractions. *Soil Sediment Contam. An Int. J.* **2015**, *24* (1), 1–15.

(19) Sungur, A.; Soylak, M.; Özcan, H. Chemical Fractionation, Mobility and Environmental Impacts of Heavy Metals in Greenhouse Soils from Çanakkale, Turkey. *Environ. Earth Sci.* **2016**, *75* (4), 1–11.

(20) Sungur, A.; Özcan, H. Chemometric and Geochemical Study of the Heavy Metal Accumulation in the Soils of a Salt Marsh Area (Kavak Delta, NW Turkey). *J. Soils Sediments* **2015**, *15* (2), 323–331.

(21) Aksoy, A. Chicory (Cichorium Intybus L.): A Possible Biomonitor of Metal Pollution. *Pak. J. Bot* **2008**, *40* (2), 791–797.

(22) Adiloğlu, S.; Sağlam, M. T. PHYTOREMEDIATION OF CHROME (Cr) POLLUTION IN AGRICULTURAL AREAS WITH CANOLA (Brassica Napus L.) PLANT GROWING. In *Proceedings of the 13th International Conference on Environmental Science and Technology*; Global Nest, Secretariat, 2013.

(23) Kurt, M. A. Comparison of Trace Element and Heavy Metal Concentrations of Top and Bottom Soils in a Complex Land Use Area. *Carpathian J. Earth Environ. Sci.* **2018**, *13* (1), 47–56.

(24) Aydinalp, C. Concentration and Speciation of Cu, Ni, Pb and Zn in Cultivated and Uncultivated Soils. *Bulg. J. Agric. Sci.* **2009**, *15* (2), 129–134.

(25) Vural, A. Contamination Assessment of Heavy Metals Associated with an Alteration Area: Demirören Gumushane, NE Turkey. *J. Geol. Soc. India* **2015**, *86* (2), 215–222.

(26) Güler, C.; Alpaslan, M.; Kurt, M. A.; Temel, A. Deciphering Factors Controlling Trace Element Distribution in the Soils of Karaduvar Industrial-Agricultural Area (Mersin, SE Turkey). *Environ. Earth Sci.* **2010**, *60* (1), 203–218.

(27) Tumuklu, A.; Yalcin, M. G.; Sonmez, M. Detection of Heavy Metal Concentrations in Soil Caused by Nigde City Garbage Dump. *Polish J. Environ. Stud.* **2007**, *16* (4).

(28) Isen, H.; Altundag, H.; Keskin, C. S. Determination of Heavy Metal Contamination in Roadside Surface Soil by Sequential Extraction. *Polish J. Environ. Stud.* **2013**, *22* (5), 1381–1385.

(29) Onder, S.; Dursun, S.; Gezgin, S.; Demirbas, A. Determination of Heavy Metal Pollution in Grass and Soil of City Centre Green Areas (Konya, Turkey). *Polish J. Environ. Stud.* **2007**, *16* (1), 145.

(30) Selçuk Zorer, Ö.; Ceylan, H.; Doğru, M. Determination of Heavy Metals and

Comparison to Gross Radioactivity Concentration in Soil and Sediment Samples of the Bendimahi River Basin (Van, Turkey). *Water. Air. Soil Pollut.* **2009**, *196* (1), 75–87.

(31) Tumuklu, A.; Ciflikli, M.; Ozgur, F. Z. Determination of Heavy Metals in Soils around Afsin-Elbistan Thermal Power Plant (Kahramanmaras, Turkey). **2008**.

(32) Bakirdere, S.; Yaman, M. Determination of Lead, Cadmium and Copper in Roadside Soil and Plants in Elazig, Turkey. *Environ. Monit. Assess.* **2008**, *136* (1), 401–410.

(33) Yalçın, İ. E.; Demir, G.; Özyiğit, İ. İ.; Doğan, İ.; Yarcı, C. Determination of Metal Deposition and Its Effects on Mineral Nutrient Uptake Status of Pinus Brutia Ten. For Assessment of Pollution Impact in Its Habitat-Istanbul. In *13th International Conference on Environmental Science and Technology*; Global Nest, 2013.

(34) Aydin, M. E.; Aydin, S.; Beduk, F.; Tor, A.; Tekinay, A.; Kolb, M.; Bahadir, M. Effects of Long-Term Irrigation with Untreated Municipal Wastewater on Soil Properties and Crop Quality. *Environ. Sci. Pollut. Res.* **2015**, *22* (23), 19203–19212.

(35) Irmak, S.; Kasap, Y.; Surucu, A. Effects of Town Waste on the Heavy Metal Contents and Plant Nutrient Element Contents of Soils in Harran Plain, Turkey. *Fresenius Environ. Bull.* **2007**, *16* (3), 285–289.

(36) Kiziloglu, F. M.; Turan, M.; Sahin, U.; Kuslu, Y.; Dursun, A. Effects of Untreated and Treated Wastewater Irrigation on Some Chemical Properties of Cauliflower (Brassica Olerecea L. Var. Botrytis) and Red Cabbage (Brassica Olerecea L. Var. Rubra) Grown on Calcareous Soil in Turkey. *Agric. water Manag.* **2008**, *95* (6), 716–724.

(37) Karataş, D.; Aydin, I.; Karataş, H. Elemental Composition of Red Wines in Southeast Turkey. *Czech J. Food Sci.* **2015**, *33* (3), 228–236.

(38) Koz, B. Energy-Dispersive X-Ray Fluorescence Analysis of Moss and Soil from Abandoned Mining of Pb-Zn Ores. *Environ. Monit. Assess.* **2014**, *186* (9), 5315–5326.

(39) Çimen, O.; Köksal, F. T.; Önal, A. Ö.; Tutay, Y. Ö. Enviromental Contamination of Heavy Metals and Chrysotile Asbestos in the Munzur and Pulumur Streams (Tunceli, Turkey). *Ofioliti* **2015**, *40* (1).

(40) Vural, A.; Gundogdu, A.; Akpinar, I.; Baltaci, C. Environmental Impact of Gümüşhane City, Turkey, Waste Area in Terms of Heavy Metal Pollution. *Nat. Hazards* **2017**, *88* (2), 867–890.

(41) Aslan, A.; Gurbuz, H.; Yazici, K.; Cicek, A.; Turan, M.; Ercisli, S. Evaluation of Lichens as Bio-Indicators of Metal Pollution. *J. Elem.* **2013**, *18* (3).

(42) Ogut, M.; Er, F.; Brohi, A. Excessive Phosphorus Fertilization Does Not Increase Cadmium Concentrations in Soil or Carrots (Daucus Carota L.) Grown in Konya (Turkey). *Acta Agric. Scand. Sect. B–Soil Plant Sci.* **2010**, *60* (5), 420–426.

(43) Guney, M.; Onay, T. T.; Copty, N. K. Impact of Overland Traffic on Heavy Metal Levels in Highway Dust and Soils of Istanbul, Turkey. *Environ. Monit. Assess.* **2010**, *164* (1), 101–110.

(44) Tug, G. N.; Duman, F. Heavy Metal Accumulation in Soils around a Salt Lake in Turkey. *Pak. J. Bot* **2010**, *42* (4), 2327–2333.

(45) Saglam, C. Heavy Metal Accumulation in the Edible Parts of Some Cultivated Plants and Media Samples from a Volcanic Region in Southern Turkey. *Ekoloji* **2013**, *22* (86),

1–8.

(46) Cevik, U.; Koz, B.; Makarovska, Y. Heavy Metal Analysis around Iskenderun Bay in Turkey. *X-Ray Spectrom. An Int. J.* **2010**, *39* (3), 202–207.

(47) Koz, B.; Cevik, U.; Akbulut, S. Heavy Metal Analysis around Murgul (Artvin) Copper Mining Area of Turkey Using Moss and Soil. *Ecol. Indic.* **2012**, *20*, 17–23.

(48) Çayır, A.; Belivermiş, M.; Kılıç, Ö.; Coşkun, M.; Coşkun, M. Heavy Metal and Radionuclide Levels in Soil around Afsin-Elbistan Coal-Fired Thermal Power Plants, Turkey. *Environ. Earth Sci.* **2012**, *67* (4), 1183–1190.

(49) Çolak, M. Heavy Metal Concentrations in Sultana-Cultivation Soils and Sultana Raisins from Manisa (Turkey). *Environ. earth Sci.* **2012**, *67* (3), 695–712.

(50) Özkul, C. Heavy Metal Contamination in Soils around the Tunçbilek Thermal Power Plant (Kütahya, Turkey). *Environ. Monit. Assess.* **2016**, *188* (5), 1–12.

(51) Yaylalı-Abanuz, G.; Tüysüz, N. Heavy Metal Contamination of Soils and Tea Plants in the Eastern Black Sea Region, NE Turkey. *Environ. Earth Sci.* **2009**, *59* (1), 131–144.

(52) Yaylalı-Abanuz, G. Heavy Metal Contamination of Surface Soil around Gebze Industrial Area, Turkey. *Microchem. J.* **2011**, *99* (1), 82–92.

(53) Güleryüz, G.; Arslan, H.; Leblebici, Z.; Kırmızı, S.; Aksoy, A.; Sakar, F. S. Heavy Metal Content of Asphodelus Aestivus Brot. from Degraded Areas in the Mediterranean Environment (Bursa, Turkey). **2014**.

(54) Arık, F.; Yaldız, T. Heavy Metal Determination and Pollution of the Soil and Plants of Southeast Tavşanlı (Kütahya, Turkey). *Clean–Soil, Air, Water* **2010**, *38* (11), 1017–1030.

(55) Pehluvan, M.; Karlidag, H.; Turan, M. Heavy Metal Levels of Mulberry (Morus Alba L.) Grown at Different Distances from the Roadsides. *J. Anim. Plant Sci.* **2012**, *22* (3), 665–670.

(56) Yalcin, M. G.; Battaloglu, R.; Ilhan, S. Heavy Metal Sources in Sultan Marsh and Its Neighborhood, Kayseri, Turkey. *Environ. Geol.* **2007**, *53* (2), 399–415.

(57) Sungur, A. Heavy Metals Mobility, Sources, and Risk Assessment in Soils and Uptake by Apple (Malus Domestica Borkh.) Leaves in Urban Apple Orchards. *Arch. Agron. Soil Sci.* **2016**, *62* (8), 1051–1065.

(58) Saglam, C. Heavy Metal Concentrations in Serpentine Soils and Plants from Kizildag National Park (Isparta) in Turkey. *Fresenius Environ. Bull.* **2017**, *26* (6), 3995–4003.

(59) Guney, M.; Zagury, G. J.; Dogan, N.; Onay, T. T. Exposure Assessment and Risk Characterization from Trace Elements Following Soil Ingestion by Children Exposed to Playgrounds, Parks and Picnic Areas. *J. Hazard. Mater.* **2010**, *182* (1–3), 656–664.

(60) Esen, A. N.; Kubešová, M.; Haciyakupoglu, S.; Kučera, J. Instrumental Neutron Activation Analysis of Plant Tissues and Soils for Biomonitoring in Urban Areas in Istanbul. *J. Radioanal. Nucl. Chem.* **2016**, *309* (1), 373–382.

(61) Yatkin, S.; Bayram, A. Investigation of Chemical Compositions of Urban, Industrial, Agricultural, and Rural Top-soils in Izmir, Turkey. *CLEAN–Soil, Air, Water* **2011**, *39* (6), 522–529.

(62) Sungur, A.; Soylak, M.; Ozcan, H. Investigation of Heavy Metal Mobility and Availability by the BCR Sequential Extraction Procedure: Relationship between Soil Properties and Heavy Metals Availability. *Chem. Speciat. Bioavailab.* **2014**, *26* (4), 219–230.

(63) Sevgi, E.; Coral, G.; Gizir, A. M.; Sangün, M. K. Investigation of Heavy Metal Resistance in Some Bacterial Strains Isolated from Industrial Soils. *Turkish J. Biol.* **2010**, *34* (4), 423–431.

(64) Altundag, H.; Albayrak, S.; Dundar, M. S.; Tuzen, M.; Soylak, M. Investigation of the Influence of Selected Soil and Plant Properties from Sakarya, Turkey, on the Bioavailability of Trace Elements by Applying an in Vitro Digestion Model. *Biol. Trace Elem. Res.* **2015**, *168* (1), 276–285.

(65) Tufekcioglu, A.; Ozbayram, A. K.; Kucuk, M. Soil Respiration in Apple Orchards, Poplar Plantations and Adjacent Grasslands in Artvin, Turkey. *J. Environ. Biol.* **2009**, *30* (5), 815.

(66) Canbay, M.; Aydin, A.; Kurtulus, C. Magnetic Susceptibility and Heavy-Metal Contamination in Topsoils along the Izmit Gulf Coastal Area and IZAYTAS (Turkey). *J. Appl. Geophys.* **2010**, *70* (1), 46–57.

(67) Gulmezoglu, N.; Aytac, Z.; Kutlu, I.; Kulan, E. G.; Gozukara, G. Mapping Boron and Beneficial Heavy Metal Ions for Wheat-Cultivating Soils in Turkey’s Boron-Mining Zone. *Appl. Ecol. Environ. Res.* **2017**, *15* (3), 1119–1130.

(68) Kaya, A.; Bağ, H. Mineral Contents of Some Wild Ascomycetous Mushrooms. **2013**.

(69) Malkoç, S.; Yazici, B. Multivariate Analyses of Heavy Metals in Surface Soil around an Organized Industrial Area in Eskisehir, Turkey. *Bull. Environ. Contam. Toxicol.* **2017**, *98* (2), 244–250.

(70) Yalcin, M. G.; Unal, B. Multivariate Statistical Approach to Identify Heavy Metal Sources in Urban Roadside Soils of Manisa, Turkey. *Asian J. Chem.* **2008**, *20* (5), 3978.

(71) Yay, O. D.; Alagha, O.; Tuncel, G. Multivariate Statistics to Investigate Metal Contamination in Surface Soil. *J. Environ. Manage.* **2008**, *86* (4), 581–594.

(72) Yildirim, D.; Sasmaz, A. Phytoremediation of As, Ag, and Pb in Contaminated Soils Using Terrestrial Plants Grown on Gumuskoy Mining Area (Kutahya Turkey). *J. Geochemical Explor.* **2017**, *182*, 228–234.

(73) Esringu, A.; Kulekci, E. A.; Turan, M.; Ercisli, S. Phytoremediation of Some Heavy Metals by Different Tissues of Roses Grown in the Main Intersections in Erzurum City, Turkey. *Fresenius Environ. Bull.* **2015**, *24* (9), 2787–2791.

(74) Pehlivan, R.; Emre, H. Potability and Hydrogeochemisty of the Sarma Stream Water, Duzce, Turkey. *Water Resour.* **2017**, *44* (2), 315–330.

(75) Karakaya, M. Ç.; Doğru, M.; Karakaya, N.; Vural, H. C.; Kuluöztürk, F.; Bal, S. Ş. Radioactivity Concentrations and Dose Assessments of Therapeutic Peloids from Some Turkish Spas. *Clay Miner.* **2015**, *50* (2), 221–232.

(76) Kilic, K.; Dogan, H. M.; Yalcin, H.; Bilim, M.; Karahan, G. Potentially Toxic Elements of Volcanic Ash Soils in the Cappadocia Region of Central Turkey. **2015**.

(77) Ak, N.; Eroğlu, E.; Güney, İ. Statistical Analysis of Soil Heavy Metals of Istanbul Children Playgrounds. *Energy Educ. Sci. Technol. Part A Energy Sci. Res.* **2012**.

(78) Kaçmaz, H.; Eran Nakoman, M. Shallow Groundwater and Cultivated Soil Suitability Assessments with Respect to Heavy Metal Content in the Köprübaşı U Mineralization Area (Manisa, Turkey). *Bull. Environ. Contam. Toxicol.* **2010**, *85* (1), 37–41.

(79) Ozdemir, T.; Batan, N.; Mendil, D.; Apaydin, G.; Cengiz, E. Some Element Levels in Moss Samples Collected from the Igdir-Nahhicevan International Highway, Turkey. **2013**.

(80) Ağca, N. Spatial Distribution of Heavy Metal Content in Soils around an Industrial Area in Southern Turkey. *Arab. J. Geosci.* **2015**, *8* (2), 1111–1123.

(81) Karanlık, S.; Ağca, N.; Yalçın, M. Spatial Distribution of Heavy Metals Content in Soils of Amik Plain (Hatay, Turkey). *Environ. Monit. Assess.* **2011**, *173* (1), 181–191.

(82) Kizilkaya, R.; Dengiz, O.; Ozyazici, M. A.; Askin, T.; Mikayilov, F.; Shein, E. V. Spatial Distribution of Heavy Metals in Soils of the Bafra Plain in Turkey. *Eurasian Soil Sci.* **2011**, *44* (12), 1343–1351.

(83) Cemek, B.; Kizilkaya, R. Spatial Variability and Monitoring of Pb Contamination of Farming Soils Affected by Industry. *Environ. Monit. Assess.* **2006**, *117* (1), 357–375.

(84) Dengiz, O.; Ozcan, H.; Koksal, E. S.; Baskan, O.; Kosker, Y. Sustainable Natural Resource Management and Environmental Assessment in the Salt Lake (Tuz Golu) Specially Protected Area. *Environ. Monit. Assess.* **2010**, *161* (1), 327–342.

(85) Kızıldağ, N.; Sağlıker, H. A.; Darıcı, C. The Comparative Evaluation of Carbon Mineralization in Soils Contaminated and Uncontaminated with Chromium. *Sains Malaysiana* **2017**, *46* (10), 1757–1762.

(86) Gülser, F.; Erdoğan, E. The Effects of Heavy Metal Pollution on Enzyme Activities and Basal Soil Respiration of Roadside Soils. *Environ. Monit. Assess.* **2008**, *145* (1), 127–133.

(87) Avci, H. Trace Metals in Vegetables Grown with Municipal and Industrial Wastewaters. *Toxicol. Environ. Chem.* **2012**, *94* (6), 1125–1143.

(88) Arslan, H.; Güleryüz, G.; Leblebici, Z.; Kırmızı, S.; Aksoy, A. Verbascum Bombyciferum Boiss.(Scrophulariaceae) as Possible Bio-Indicator for the Assessment of Heavy Metals in the Environment of Bursa, Turkey. *Environ. Monit. Assess.* **2010**, *163* (1), 105–113.

(89) USEPA. *Exposure Factors Handbook: 2011 Edition*; 2011.

(90) Cetin, B.; Yurdakul, S.; Gungormus, E.; Ozturk, F.; Sofuoglu, S. C. Source Apportionment and Carcinogenic Risk Assessment of Passive Air Sampler-Derived PAHs and PCBs in a Heavily Industrialized Region. *Sci. Total Environ.* **2018**, *633*, 30–41.

(91) USEPA. EPA IRIS https://www.epa.gov/iris (accessed Nov 20, 2021).

(92) RAIS. The Risk Assessment Information System https://rais.ornl.gov (accessed Nov 20, 2021).
